# Supplementary material for: Protocol for the Tessa Jowell BRAIN MATRIX Platform Study
Source: BMJ Open. 2022 Sep 8;12(9):e067123. doi: 10.1136/bmjopen-2022-067123 (PMC9462095; doi:10.1136/bmjopen-2022-067123)
Supplement: Supplementary data [file bmjopen-2022-067123supp003.pdf]

Supplementary Appendix 3: The Tessa Jowell BRAIN MATRIX Platform study World Health Organization Trial Registration Data Set

Data category	Information
Primary registry and trial identifying number	ClinicalTrials.gov<br>NCT04274283
Date of registration in primary registry	18-Feb-2020
Secondary identifying numbers	ISRCTN 14218060
Source(s) of monetary or material support	The Brain Tumour Charity<br>Genomics England<br>INNOVATE UK
Primary sponsor	University of Birmingham
Secondary sponsor(s)	n/a
Contact for public queries	Dr Joshua Savage: brainmatrix@trials.bham.ac.uk
Contact for scientific queries	Prof. Colin Watts: c.watts.2@bham.ac.uk
Public title	The Tessa Jowell BRAIN MATRIX Platform Study
Scientific title	A British Feasibility Study of Molecular Stratification and Targeted Therapy to Optimize the Clinical Management of Patients With Glioma by Enhancing Clinical Outcomes, Reducing Avoidable Toxicity, Improving

Data category	Information
	Management of Post-operative Residual & Recurrent Disease and Improving Survivorship
Countries of recruitment	UK
Health condition(s) or problem(s) studied	Newly diagnosed suspected, or progressive with known, WHO Grade 2-4 glioma
Intervention(s)	None
Key inclusion and exclusion criteria:	Ages eligible for study: ≥16 years<br>Sexes eligible for study: both<br>Accepts healthy volunteers: no
	Inclusion criteria: Newly diagnosed suspected, or progressive with known, WHO Grade 2-4 glioma
	Exclusion criteria: Primary spinal cord tumours, active treatment of other malignancy
Study type	Observational
	Primary purpose: Feasibility of establishing an integrated histological-molecular diagnosis of glioblastoma
Date of first enrolment	14-Dec-2020
Target sample size	1000
Recruitment status	Open
Primary outcome(s)	• Time (from biopsy) to integrated histological–molecular diagnosis (TTMD) using whole genome sequencing and epigenomic classification

**Data category**	**Information**
Key secondary outcome(s)	• Time to completion of each node of tissue and imaging pathway.<br>• Tumour and biological sample(s) quality control (QC) status.<br>• Imaging QC status.<br>• Inter-rater agreement of RANO assessments.

Watts C, *et al. BMJ Open* 2023; 12:e067123. doi: 10.1136/bmjopen-2022-067123
