# Supplementary material for: Protocol for the Tessa Jowell BRAIN MATRIX Platform Study
Source: BMJ Open. 2022 Sep 8;12(9):e067123. doi: 10.1136/bmjopen-2022-067123 (PMC9462095; doi:10.1136/bmjopen-2022-067123)
Supplement: Author's manuscript [file bmjopen-2022-067123.draft_revisions.pdf]

BMJ Open is committed to open peer review. As part of this commitment we make the peer review history of every article we publish publicly available.

When an article is published we post the peer reviewers' comments and the authors' responses online. We also post the versions of the paper that were used during peer review. These are the versions that the peer review comments apply to.

The versions of the paper that follow are the versions that were submitted during the peer review process. They are not the versions of record or the final published versions. They should not be cited or distributed as the published version of this manuscript.

BMJ Open is an open access journal and the full, final, typeset and author-corrected version of record of the manuscript is available on our site with no access controls, subscription charges or pay-per-view fees (http://bmjopen.bmj.com).

If you have any questions on BMJ Open's open peer review process please email info.bmjopen@bmj.com

BMJ Open

# Protocol for the Tessa Jowell BRAIN MATRIX Platform Study



| | |
|---|---|
| Journal: | *BMJ Open* |
| Manuscript ID | bmjopen-2022-067123 |
| Article Type: | Protocol |
| Date Submitted by the Author: | 07-Aug-2022 |





| | |
|---|---|
| Complete List of Authors: | Watts, Colin; University of Birmingham College of Medical and Dental Sciences; University Hospitals Birmingham NHS Foundation Trust<br>Savage, Joshua; University of Birmingham College of Medical and Dental Sciences<br>Patel, Amit; University of Birmingham College of Medical and Dental Sciences<br>Mant, Rhys; University of Birmingham College of Medical and Dental Sciences<br>Wykes, Victoria; University of Birmingham College of Medical and Dental Sciences; University Hospitals Birmingham NHS Foundation Trust<br>Pohl, Ute; University Hospitals Birmingham NHS Foundation Trust<br>Bulbeck, Helen; Brainstrust<br>Apps, John; University of Birmingham College of Medical and Dental Sciences<br>Sharpe, Rowena; University of Birmingham College of Medical and Dental Sciences<br>Thompson, Gerard; University of Edinburgh Brain Research Imaging Centre<br>Waldman, Adam; University of Edinburgh Brain Research Imaging Centre<br>Ansorge, Olaf; Oxford University<br>Billingham, Lucinda; University of Birmingham College of Medical and Dental Sciences |





| | |
|---|---|
| Keywords: | NEUROSURGERY, Neurological oncology < ONCOLOGY, Clinical trials < THERAPEUTICS |
| | |



SCHOLARONE™ Manuscripts

# Protocol for the Tessa Jowell BRAIN MATRIX Platform Study

Colin Watts[1†,2], Joshua Savage[3], Amit Patel[3], Rhys Mant[3], Victoria Wykes[1,2], Ute Pohl[2], Helen Bulbeck[4], John Apps[3], Rowena Sharpe[3], Gerard Thompson[5], Adam Waldman[5], Olaf Ansorge[6], Lucinda Billingham[3] and the TJBM Investigators*

[†] Corresponding author contact details:

Professor Colin Watts. Institute of Cancer and Genomic Sciences, University of Birmingham, Edgbaston, Birmingham. UK. Email: C.Watts.2@bham.ac.uk

*Additional Investigators of the BRAIN MATRIX study are listed in Supplementary Appendix 1.

[1] Institute of Cancer and Genomic Sciences, University of Birmingham, Edgbaston, Birmingham. UK

[2] University Hospitals Birmingham NHS Foundation Trust, Birmingham. UK

[3] Cancer Research UK Clinical Trials Unit (CRCTU), University of Birmingham, Edgbaston, Birmingham. UK

[4] brainstrust, Cowes, Isle of Wight. UK

[5] Centre for Clinical Brain Sciences, University of Edinburgh, Edinburgh. UK

[6] Nuffield Department of Clinical Neurosciences, University of Oxford, Oxford. UK

For peer review only

## Abstract

### Introduction

Gliomas are the most common primary tumour of the central nervous system (CNS), with an estimated annual incidence of 6.6 per 100,000 individuals in the USA and around 14 deaths per day from brain tumours in the UK. The genomic and biological landscape of brain tumours has been increasingly defined and, since 2016, the WHO classification of tumours of the CNS incorporates molecular data, along with morphology, to define tumour subtypes more accurately. The Tessa Jowell BRAIN MATRIX Platform (TJBM) study aims to create a transformative clinical research infrastructure that leverages UK NHS resources to support research that is patient centric and attractive to both academic and commercial investors.

### Methods and analysis

The TJBM study is a programme of work with the principal purpose to improve the knowledge of glioma and treatment for glioma patients. The programme includes a platform study and subsequent interventional clinical trials (as separate protocols). The platform study described here is the backbone data-repository of disease, treatment and outcome data from clinical, imaging and pathology data being collected in glioma patients from secondary care hospitals. The primary outcome measure of the platform is time from biopsy to integrated histological-molecular diagnosis using whole genome sequencing and epigenomic classification. Secondary outcome measures include those that are process-centred, patient-centred and framework-based. Target recruitment for the study is 1000 patients with interim analyses at 100 and 500 patients.

### Ethics and dissemination

The protocol was approved by West Midlands - Edgbaston Research Ethics Committee. Participants will be required to provide written informed consent. The results of this study will be disseminated through national and international presentations and peer-reviewed publications.

### Trial registration number

ClinicalTrials.gov Identifier: NCT04274283; 18-Feb-2020

ISRCTN 14218060; 03-Feb-2020


For peer review only

## Strengths and limitations of this study

- The TJBM study is a programme of work, which will improve the knowledge of glioma, and treatment for glioma patients.
- This platform study is a backbone data-repository of disease, treatment and outcome data from clinical, imaging and pathology data
- The TJBM study will aim to provide rapid and accurate molecular diagnosis, a network of clinical hubs with robust protocols for the collection, processing, analysis and storage of tissue, images, and clinical and quality of life data
- Gliomas occur at all ages and their specific subtype is difficult to predict preoperatively, therefore, the patient population eligible for the study is broad but currently excludes <16-year-old patients

## Introduction

Gliomas are the most common primary tumour of the central nervous system (CNS), with an estimated annual incidence of 6.6 per 100,000 individuals in the USA (1), which is predicted to rise to 22/100,000 by 2035 (2). In 2016, there were 5,250 deaths from brain tumours in the UK i.e., 14 deaths per day (2). Malignant CNS tumours hold the poorest prognosis, and are responsible for the highest estimated number of years of potential life lost (mean 20 years) amongst all cancers (3) and survival trends have remained generally static in comparison to other cancers (4).

Gliomas have traditionally been divided into low grade glioma (LGG; WHO I-II) and high-grade glioma (HGG; WHO III-IV) based on integrated classic histological features and molecular biomarkers (5). LGG have an indolent course with patients commonly surviving a decade after diagnosis (6). However, the natural history of many WHO Grade II LGG is progression to HGG. Approximately half of all newly diagnosed gliomas are classified as glioblastoma (GB; WHO IV), the most malignant type of brain cancer. Currently, the GB annual incidence is 3.2 per 100,000 population in the USA. This tumour occurs more frequently with advancing age; ranging from 0.4 per 100,000 population aged 20–34 years, to over 15 per 100,000 population aged 75-84 years (1). It is widely recognized that elderly populations are rapidly increasing globally and this will have a significant impact on the burden of GB disease. Despite this, most studies still focus on patients younger than 65 years.

The current gold standard treatment for newly diagnosed GB is surgical gross total resection (GTR), followed by radiotherapy with concomitant and adjuvant temozolomide. The aim of treatment is to delay tumour progression and extend overall survival (7). Despite decades of refinement, this approach results in a median survival time of only 12-14 months.

Over the last 20 years the genomic and biological landscape of brain tumours has been increasingly defined, refining previous classification systems, unravelling intra- and inter-tumoural heterogeneity and progression, identifying drug targets and potential therapeutic strategies, and better characterising challenges to therapies, such as the blood brain barrier (BBB) and mutational escape. In 2016, the WHO published its classification of tumours of the CNS (5), which for the first time used molecular data, along with morphology, to define tumour subtypes more accurately. This stratification integrated a combination of specific genetic and epigenetic biological characteristics that are changing rapidly as our knowledge evolves (8). In recognition of this rapid evolution of knowledge, 'cIMPACT-NOW' (Consortium to Inform Molecular and Practical Approaches to CNS Tumor Taxonomy – Not Official WHO) (9) was created, with the new WHO 2021 classification representing a consensus opinion based on new insights into the molecular definition of brain tumours from this collaboration (10). As analytical technologies evolve and become more affordable, genome-wide analyses combined with other so-called 'omics' data will lead to further refinement of tumour classification with meaningful impacts on prognosis and choice of therapy. This is the motivation for the TJBM study, which will adopt whole-genome sequencing (WGS) and epigenomic analysis of tumour samples.

The TJBM study aims to create a transformative clinical research infrastructure that leverages NHS resources to support research that is patient centric and attractive to both academic and commercial investors. Our approach will be holistic and flexible so that it can rapidly adapt to scientific advances and rigorously evaluate new drugs and technologies. To achieve this, it will establish a research-active network of clinicians and scientists who are well supported by an innovative trial infrastructure to deliver the following:

For peer review only - http://bmjopen.bmj.com/site/about/guidelines.xhtml

- Rapid and accurate molecular diagnosis ensuring precise classification of tumours and identifying subsets of patients suitable for targeted therapy, by building on the legacy of the 100,000 Genomes Project (11).
- A network of clinical hubs resourced to maximise patient recruitment and collect tissue and data.
- Robust protocols for the collection, processing, analysis and storage of tissue, images and clinical and quality of life (QoL) data, building on existing infrastructure such as UK Biobank (12), Medical Research Council (MRC) Brain Banks Network (13), and the CRUK PEACE study (14).
- High-quality biological samples that are fully clinically and radiologically annotated facilitating further biological and radiological research.
- Links to national and international clinical and scientific infrastructure and networks (15); e.g., Genomics England (GEL), European Network for Rare adult solid Cancer (EURACAN) (16), SIOP-E Brain Tumour Group, and National Cancer Research Institute (NCRI) Groups.
- Access to novel and repurposed drugs and technologies through collaborative partnerships with industry and early phase trials hubs and the structural genomics consortium (www.thesgc.org), to develop novel trials, within and outside, the TJBM infrastructure for testing of therapeutic strategies, including novel agents. As such therapeutic clinical trials will be developed using this infrastructure to maximise efficiency in introducing and rigorously evaluating novel interventions. These will be separate protocols to the TJBM study.
- Long-term sustainability through delivery of clinically and scientifically meaningful outcomes, leveraged investigator-led research grants and an established cost-recovery model for biobanking (17).

# Methods and analysis

## Study design

The TJBM study is a programme of work the principal purpose of which is to improve the knowledge of glioma and treatment for glioma patients. The programme will include a platform study and subsequent interventional clinical trials (as separate protocols); Figure 1. This platform study, is the backbone data-repository of disease, treatment and outcome data from clinical, imaging and pathology data to be collected in glioma patients from secondary care hospitals. Figure 2 shows an overview of the platform's study schema.

The study aims to recruit 1000 patients within the UK over 5 years with participants followed up for up to 5 years. An initial 10 UK centres were opened to the TJBM study, as listed in Supplementary Appendix 1, although further centre expansion is planned. The Standard Protocol Items: Recommendations for Intervention Trials (SPIRIT) checklist is provided as Supplementary Appendix 2 (18). The World Health Organization (WHO) Trial Registration Data Set is provided in Supplementary Appendix 3.

## Patient and public involvement

Patient and public involvement and engagement (PPIE) has been integral to this study from its inception. Our patient and public advisors, Helen Bulbeck and Peter Buckle, co-developed the TJBM study by reviewing and refining the protocol and the participant-facing documents. They have provided input into the patient-reported outcome measures and have guided messaging about the study for the community. As members of the Study Management Group (SMG; Supplementary Appendix 1) they continue to assess study conduct, and will contribute to the interpretation and dissemination of study findings through the

For peer review only

PPIE dissemination strategy. This will include presentation of the study findings to identified audiences, the best channel for reach, when to communicate, messaging and sensitivities.

## Patient selection

Patients aged ≥16 years with newly diagnosed suspected WHO Grade II-IV glioma, (as evidenced radiologically) suitable for a diagnostic or therapeutic surgical procedure resulting in a tumour sample matched to a blood sample or with progression with known WHO Grade II-IV glioma will be eligible.

Patients with primary spinal cord tumours, who are receiving active treatment of other malignancy, have contraindications to magnetic resonance imaging (MRI) and/or without standard of care imaging available, will be excluded.

Newly diagnosed patients with suspected WHO Grade II-IV glioma who are subsequently found to have a WHO Grade I tumour or non-brain tumour are expected to be a rare occurrence. If this event occurs:

- If it is confirmed as a Grade I tumour, then the patient will remain eligible for the study and will continue to be followed up in accordance with the protocol.
- If it is confirmed as a non-brain tumour, then the patient will not remain on the study and no further follow-up data will be collected.

All patients must be able to provide written informed consent for the study.

## Consent

Supplementary Appendix 4 contains exemplar informed consent forms (ICF), with Supplementary Appendix 5 the patient information sheets (PIS) and lay summary for the study. The investigator or an appropriately trained delegate must obtain written informed consent for each patient prior to performing any study related procedure. Remote Consent for platform entry is permitted by telephone or video consultation instead of face-to-face consultations.

In addition, to consenting to join TJBM, patients will be required to provide agreement for:

- The collection and analysis of biological samples (e.g., tumour, blood), including access to existing and future samples.
- The collection of relevant clinical information, including imaging and pathology.
- The return of clinically relevant results back to the referring clinician.
- The use of, and sharing of data, for research, teaching, commercial and scientific purposes, including data sharing through The Brain Tumour Charity's BRIAN (the Brain tumouR Information and Analysis Network) database (19).
- The collection of different aspects of health data from the NHS and other Department of Health organisations (in addition to medical records) for longitudinal analysis and follow-up.
- The use of clinical data to identify potential clinical trials or other research that they may benefit from.
- The sharing of samples for other ethically approved research projects.

In addition, and in line with GEL processes and studies such as the 100,000 Genomes Project, optional consent will be taken as to whether the patient would like to receive feedback about the evidence of inherited diseases (both underlying the cause of the cancer and non-cancer causes).

For peer review only

For centres submitting tumour tissue for WGS through the standard of care (SoC) NHS Genomic Medicine Service (GMS) pathway in England, an additional GEL consent step is currently required to confirm consent at the point of referral for the patient's clinical indication. This additional GEL consent step is performed electronically or on paper and can be completed over the phone.

## Platform assessments and schedule of events

Platform entry requires baseline clinical data, NIH Stroke Scale (20), weight, and WHO Performance Status to be recorded, and then performed again at start and end of concomitant therapy, start and end of adjuvant therapy, further surgery and during the five-year follow-up period.

In addition, QoL questionnaires will be completed at the initiation of concomitant therapy, initiation of adjuvant therapy, further surgery and during follow-up. The QoL booklet includes EQ-5D-5L (21), EORTC-QLQ-c30 (22), Patient Concerns Inventory (PCI) (23), Patient Global Impression of Change (PGIC) (24), and Clinician Global Impression of Change (25).

The schedule of events in included in Supplementary Appendix 6.

## Platform study outcome measures

The primary outcome measure of the platform is time from biopsy to integrated histological–molecular diagnosis (TTMD) as defined as the difference (in days) between date of biopsy and date of WGS and epigenomic classification.

Secondary outcome measures include those that are process-centred, patient-centred, and framework-based.

Process-centred secondaries include: time to completion of each node of tissue and imaging pathway; tumour and biological sample(s) quality control (QC) status; imaging QC status, and; inter-rater agreement of response assessment in neuro-oncology (RANO) (26).

Patient-centred secondaries include: extent of surgical resection; overall survival time (OS); intracranial progression-free survival time (PFS); QoL scores; type of interventions received; type of complications from treatments (standard of care) received, and; concordance between initial local radiological diagnosis, local pathological diagnosis, and integrated histological-molecular diagnosis.

Research framework-based secondaries include: samples and images centrally stored; targetable mutation(s) identified; post-mortem sampling consent status and sample collection confirmation, and; number of applications to, and outputs resulting from data repository (including trial proposals both within and outside of the TJBM network).

## Statistical analysis plan

The target sample size for the study is 1000 patients. The primary remit of TJBM study is to establish a central data repository that will support the development and delivery of precision medicine for all glioma patients in the UK. As such, there is no statistical basis behind the choice of target sample size, but this number will allow robust assessment of feasibility and subgroup analyses. Sample size for any clinical trials that are subsequently linked to the platform will be based on statistical justification.

A formal interim analysis of the primary outcome measure (TTMD) and any relevant secondaries will be performed after registration and diagnosis of the first 100 patients and after 500 patients. There are no

For peer review only - http://bmjopen.bmj.com/site/about/guidelines.xhtml

formal stopping rules. Formal analyses of all study outcome measures will be performed once the study has completed recruitment (target 1000 patients) and completed the follow up for all registered patients.

The analysis of TTMD will essentially be descriptive. The median TTMD will be reported overall and for each centre, together with the proportion of patients achieving TTMD within 28 days. Graphs of the change in both these summary statistics over time will be used to explore if TTMD changes during the course of the study. All estimates will be accompanied by 95% confidence intervals (CI). The time to completion of each node of tissue and imaging pathway will be reported as medians together with 95% CI, both overall and for each centre. Swimmer plots will be used to depict overall and node level timings for each patient.

For each type of tumour and biological sample, the proportion of sample passing QC and successfully undergoing WGS and EC will be reported with 95% CI and similarly for the imaging QC outcome measures. Inter-rater agreement of RANO will be assessed through Kendall's coefficient of concordance.

Extent of surgical resection is evaluated from the post-operative MRI scan and is categorised as either; closed biopsy, open biopsy, debulking <50%, subtotal resection 50-90%, near total resection 90-<100%, gross total resection 100%. The extent of surgical resection will be reported as the proportion falling into each category together with 95% CI.

OS is defined as the time from date of diagnosis to the date of death with patients who are alive at the time of analysis censored at the date last seen in clinic. Intracranial PFS time is defined as the time from date of registration to the earliest of date of intracranial progressive disease or death from disease. The date of an event is defined as the earliest confirmation of progression by radiological assessment, clinical symptoms or multidisciplinary team (MDT). Patients without progression at the time of analysis will be censored at the date last seen in clinic.

OS and intracranial PFS will be analysed and plotted using the Kaplan-Meier method. Median times with corresponding 95% CI will be reported together with rates at 1, 2 and 5 years. Multivariable survival regression modelling will be used to explore prognostic factors, including, but not limited to, WHO 2021 classification, age, tumour volume, stage, methylation and mutation status.

Longitudinal measures of QoL will be generated from the QoL questionnaire according to the questionnaire-specific algorithms for scoring. The analysis of longitudinal QoL scores will essentially be descriptive. For each of the multiple QoL scores generated from the different questionnaires, the means, medians or proportions (as appropriate) will be plotted over time together with 95% CI. These repeated measures over time may be modelled, if appropriate, with a linear or more flexible mixed model that takes account of the within-subject correlation and will allow exploration of factors associated with the outcome.

Details of the type of intervention received and complications (e.g., surgical wound infection) relating to standard of care treatments received will be monitored and recorded throughout the follow-up period and reported descriptively as frequencies and associated percentages. In relation to initial local logical diagnosis, local pathological diagnosis and integrated histological-molecular diagnosis, any difference between the tiers of diagnoses will be highlighted and categorised as: discordant; agreed; refined and reported descriptively as frequencies and associated percentages.

Confirmation of central storage of images and material, relevant targetable mutations identified by WGS and epigenomic classification, and receipt of post-mortem consent forms with confirmed central storage of samples will be recorded and reported descriptively.

For peer review only

Planned subgroup analyses of outcome measures include: IDH mutated and wildtype tumours; residual enhancing disease (RED; none, operable, inoperable); methylated and un-methylated tumours; age groups; types of diagnosis (WHO 2021 criteria only, epigenetic classification only, WGS analysis only, integrated diagnosis comprising all three); performance status; sex, and; biomarkers that emerge during the study (either discovered in the TJBM study or reported in the literature) that are deemed relevant after review by the Scientific Advisory Board (SAB).

## Biological samples

### Sample collection

Biological samples will be collected at each participating site following agreed protocols and guidance from the central biospecimen coordination centre (Oxford BRAIN MATRIX Laboratory). The aim is to build as much as possible on existing GEL infrastructure and pathways. The funded pathway is represented in Figure 3.

Fresh tissue for TJBM must be frozen in liquid nitrogen and stored until shipment to the Oxford BRAIN MATRIX Laboratory. Matched 'germline' DNA from white blood cells is required for the detection of somatic variants for paired blood/tumour WGS. This should be collected as per standard GEL protocols. Ideally, it should be collected prior to or at the time of first surgery. The blood sample(s) must be shipped together with the frozen tissue of the patient to the Oxford BRAIN MATRIX Laboratory.

For patients who are not undergoing surgery and have available tumour samples from previous tumour surgery, blood should be collected and sent to the Oxford BRAIN MATRIX Laboratory along with their tumour samples.

For participants unable to donate a blood sample, a saliva sample is an acceptable alternative.

Where possible, it is intended to collect blood samples for liquid biopsies of the tumour (e.g., tracking circulating tumour cell free (cft)DNA within the blood). Samples should ideally be collected at the time of operation, before the neurosurgeon performs any incision, ideally in theatres or the anaesthetics room just before any biopsy. Where feasible further blood samples should be collected at the following key treatment milestones:

1. At first post-operative MRI
2. Initiation of concomitant therapy (if applicable).
3. The end of concomitant therapy (if applicable).
4. Initiation of adjuvant therapy (if applicable).
5. The end of adjuvant therapy (if applicable).
6. The time of objectively measured progression.
7. During the palliative phase if a post-mortem has been agreed.

Where cerebrospinal fluid (CSF) is available for the patient, this should also be submitted.

Participating sites should follow the CRUK PEACE study protocol for post-mortem neurological tissue donation. The aim of post-mortem tissue donation is to enable research into:

1. The clonal evolution of the glioma after emergence of therapy resistance.
2. Tumour host-interaction at the whole brain level.
3. The effect of radio-chemotherapy on normal brain.

For peer review only

4. The interaction between the glioma and non-CNS organs (e.g., immune system in the cervical lymph nodes).

**Sample analyses**

Matched tumour/blood samples collected at first surgery are the most important samples of the TJBM study as these determine the initial integrated histological-molecular diagnosis. Following histological QC, the Oxford BRAIN MATRIX Laboratory will perform DNA extraction and QC; an aliquot will be sent to the Illumina Centre in Cambridge for WGS, and one used for EPIC methylation array at the Wellcome Trust Centre for Human Genetics in Oxford. Remaining DNA and unused tissues will be stored at the BRAIN MATRIX Biorepository.

Raw WGS data will be maintained in the GEL Data Research Environment, where it can be accessed and re-analysed by data scientists at the submitting NHS Genomic Laboratory Hub or any qualifying TJBM approved researcher. Sample data not generated by GEL will be consolidated in established bioinformatics hubs of the University of Oxford (Big Data Institute/Weatherall Institute for Molecular Medicine).

Data files on the Illumina 850k EPIC BeadChip analysis will be uploaded to the 'Heidelberg Classifier' hub at the German Cancer Centre in Heidelberg (27). This will generate an automated classifier report which will be stored at the Oxford BRAIN MATRIX Lab. Raw array data will be made available via the Oxford BRAIN MATRIX Laboratory.

Matched histological sections will be digitised at the Oxford BRAIN MATRIX Laboratory resulting in linked genomic data, which will initially be stored in Oxford. As the study evolves, we will aim to capture digital histological data from the material kept at the local neuropathology centre. The Oxford BRAIN MATRIX hub is working with CRUK-established data visualisation platforms, such as those developed for S:CORT (Colorectal Cancer) (28) and based on international open access platforms (such as cBioPortal (29)).

The BRAIN MATRIX neuropathology and genomics team will generate an integrated report (histology, WGS, Heidelberg Classifier) for each case in consultation with the local neuropathology team. The primary BRAIN MATRIX report will comprise the formal routine GEL WGS report (germline and tumour) and Heidelberg Classifier report and integrate this with the histological and molecular report issued by the recruiting site. It is anticipated that a local histological and molecular diagnosis using immunohistochemical surrogate markers and targeted genetic analyses will be available before WGS and Illumina EPIC BeadChip data are returned to the Oxford BRAIN MATRIX Lab. When available, the BRAIN MATRIX neuropathology and molecular genetics team will conduct a virtual MDT with the referring site to ensure all relevant information will be incorporated in the final BRAIN MATRIX diagnostic report. Variant calling and classifier outputs will be determined by the practice at the GEL/Illumina Centre and the version of the Heidelberg Classifier algorithm active at the time of sample analysis.

Where relevant germline data is identified, local sites should facilitate local genetic referral as per other GEL study protocols.

## Imaging data

Pseudo-anonymised longitudinal clinical imaging (MRI) for each patient will be collected and stored at a central imaging hub overseen by the Edinburgh Imaging Hub. Disease response assessment will be performed by practicing UK neuro-radiologists with a neuro-oncology interest via the Edinburgh Imaging Platform and additional analyses undertaken through the University of Edinburgh image analysis laboratory with permitted partners.

For peer review only

As per patient standard of care, it is expected that imaging will be performed pre- and post-operatively, for radiotherapy planning, following any chemoradiation, and as per follow-up determined by the managing MDT and/or if clinical concerns are raised regarding disease progression.

Pragmatic MRI protocols will be conducted as per standard of care MRI protocols and timing following diagnosis and as such will be informed by the National Institute for health and Care Excellence (NICE) guidelines from 2018 (30), in line with the recent British Society of Neuroradiologists (BSNR) imaging guidance (31), which is itself an implementation of the Brain Tumour Imaging Protocol proposed by Ellingson *et al.* (32). Where additional advanced imaging is performed this is also encouraged to be submitted to the Edinburgh Imaging Hub and will be catalogued to permit any relevant subsequent analysis. Biopsy location imaging should also be submitted, and standard operating protocols for the major neuro-navigation systems followed. Radiotherapy planning imaging will also be submitted.

The RANO assessments will be performed through a secure web portal provided by QMENTA Inc. There will be an ongoing 10% re-read to assess inter-rater agreement. It is anticipated that RANO reports will be provided within 2 weeks (10 working days) of successful transfer of imaging requiring assessment. RANO reports will categorise response where possible into Complete Response (CR), Partial Response (PR), Stable Disease (SD) or Progressive Disease (PD). In cases of diagnostic uncertainty, potential or provisional outcomes may be recorded, allowing progression events to be backdated to the correct time point should subsequent imaging be confirmatory.

## Adverse events reporting

There are no study treatments within the TJBM study. Blood sampling and completion of QoL questionnaires are the only procedures that patients undergo additional to usual care. Therefore, it is not anticipated that there will be adverse events related to participation in this study. Only severe adverse events (SAEs) relating to those additional procedures will be reported as per Common Terminology Criteria for Adverse Events (CTCAE) version 4 (33) and as defined in Supplementary Appendix 7. The reporting period is from the date of informed consent to death.

## Data management

Case report forms will be entered online via a secure web-based portal. Authorised staff at sites will require an individual secure login username and password to access this online data entry system. Paper CRFs will be available for backup only and must be completed, signed/dated and returned to the BRAIN MATRIX Study Office by the investigator or an authorised member of the site research team. Data reported on each CRF should be consistent with the source data or the discrepancies should be explained. If information is not known, this must be clearly indicated on the CRF. All missing and ambiguous data will be queried. All sections are to be completed.

All study records must be archived and securely retained for at least 25 years. No documents will be destroyed without prior approval from the sponsor, via the central Study Office. On-site monitoring will be carried out as required following a risk assessment and as documented in the Quality Management Plan. Any monitoring activities will be reported to the central BRAIN MATRIX Study Office and any issues noted will be followed up to resolution. BRAIN MATRIX will also be centrally monitored, which may trigger additional on-site monitoring. Further information regarding data management is provided in the study protocol.

For peer review only

The CRCTU will hold the final study dataset and will be responsible for the controlled sharing of anonymised data with the wider research community to maximise potential patient benefit while protecting the privacy and confidentiality of study participants. Data anonymised in compliance with the Information Commissioners Office requirements, using a procedure based on guidelines from the Medical Research Council (MRC) Methodology Hubs, will be available for sharing with researchers outside of the trials team within 12 months of the primary publication.

## Trial organisation structure

The University of Birmingham will act as single sponsor to this multi-centre study: Support Group, Aston Webb Building, Room 119, Birmingham, B15 2TT. Email: researchgovernance@contacts.bham.ac.uk). The study is being conducted under the auspices of the CRCTU, University of Birmingham according to their local procedures.

The Chief Investigator, Co-investigators, Trial Management Team Leader, Senior Trial Coordinator, Trial Coordinator, Lead and Trial Statistician, Trial Monitor and patient representatives will form the Study Management Group (SMG; current membership is listed in Supplementary Appendix 1). The SMG will be responsible for the day-to-day conduct of the TJBM study, meeting at regular intervals (e.g., at least every three months), or as required, usually by teleconference. They will be responsible for the set-up, promotion, on-going management of the study, the interpretation of the results and preparation and presentation of relevant publications.

Selected findings of clinical significance will be presented to the Scientific Advisory Board (SAB), which as a minimum will include the Chief Investigator, an oncologist, a pathologist, and a molecular biologist for a combined review of the molecular findings in context. The SAB may suggest amendments that will be incorporated into a SAB Report, which will be sent to the Executive Oversight Committee (EOC); current SAB and EOC membership is listed in Supplementary Appendix 1. Possible re-testing/further testing may be required, as a result of SAB feedback. Guided decision-making tools that review results in the context of the literature and clinical experience will be piloted within the SAB. The SAB will also evaluate research and study proposals, manage data/tissue requests, and will report back to the EOC regarding any proposals or changes which they may suggest.

The overarching remit of the EOC is to mandate, including timeframe and deliverables, the responsibility of the SAB and to take responsibility of horizon scanning to enable the incorporation of new interventional arms. They will oversee the overall study management of both the platform and any standalone interventional trials. They will also liaise with other trial units and pharma stakeholders as well as funders, charities, study sponsors, and policymakers and liaise with the BRIAN team. A quarterly EOC Report will be disseminated to all stakeholders that will demonstrate the performance metrics of each clinical site. In addition, the use of all samples given to external researchers via the SAB will be included in the report.

## Confidentiality

Confidential information collected during the study will be stored in accordance with the General Data Protection Regulation (GDPR) 2018. As specified in the patient information sheet (PIS) and with the patients' consent, patients will be identified using only their date of birth and unique study ID number. Authorised staff may have access to the records for quality assurance and audit purposes. The BRAIN MATRIX Study Office maintains the confidentiality of all patients' data and will not disclose information by which patients may be identified to any third party other than those directly involved in the treatment of

For peer review only

the patient and organisations for which the patient has given explicit consent for data transfer (e.g., laboratory staff).

### Trial status

Recruitment for the study opened in Nov-2020 and recruitment is expected to last for 5 years.

## Ethics and dissemination

The study will be performed in accordance with the recommendations guiding physicians in biomedical research involving human subjects, adopted by the 18th World Medical Association General Assembly, Helsinki, Finland and stated in the respective participating countries laws governing human research, and Good Clinical Practice. The protocol was initially approved on 18-Feb-2020 by West Midlands - Edgbaston Research Ethics Committee; the current protocol (v3.0) was approved on 15-Jun-2022.

A meeting will be held after the end of the study to allow discussion of the main results among the collaborators prior to publication. The results of this study will be disseminated through national and international presentations and peer-reviewed publications.

Manuscripts will be prepared by the SMG and authorship will be determined by mutual agreement.

## Discussion

### Justification for patient population

The main aim of the TJBM study is to test the hypothesis that comprehensive genomic and epigenomic profiling of gliomas is feasible in a timely manner in the UK, and that the results improve stratification of patients for next generation (targeted) therapies, ultimately improving outcomes and QoL.

Gliomas occur at all ages and their specific subtype is difficult to predict preoperatively. Therefore, the patient population eligible for the TJBM study is broad. It includes any patient who on preoperative assessment is suspected to have a diffuse glioma, or where a diffuse glioma remains a credible differential diagnosis, as established by the multidisciplinary team at the recruiting site.

Recruitment is also open to patients with diffuse or atypical gliomas who had a biopsy before the launch of the TJBM study. This approach ensures that:

1. Patients with slow-growing diffuse gliomas that evolve over many years (oligodendrogliomas) may benefit from one or more of the clinical trials developed as part of the TJBM study and sample collection at tumour progression.
2. Patients who potentially benefit most from comprehensive molecular diagnostics are not missed, which often are those with rare or atypical variants of glioma based on current diagnostics (34).

The infrastructure for children with brain tumours in the UK is different to that of adults with epigenomic classification regularly used and with WGS shortly to be made available for all children with cancer. Thus, the challenges and opportunities are different for paediatric patients. Nevertheless, it is intended that children will be included within the TJBM study wherever appropriate. The recruitment of children and adolescents into the TJBM study will be coordinated with the UK's paediatric oncology expert group(s).

For peer review only

## Justification for methodology

Neuroimaging with MRI plays a central role in the initial diagnosis and treatment stratification, surgical and radiotherapy planning and assessment of disease response and progression in glioma.

Potential participants will have undergone MRI as part of a diagnostic work-up and an intracranial mass identified. As per current clinical practice, expert neuroradiological review will indicate glioma as the likely diagnosis, and a plan for treatment will be made by the relevant regional MDT meeting. Those with suspected glioma on MRI who are to undergo surgery and are otherwise eligible for the TJBM study will be approached. A proportion of these lesions that are subsequently diagnosed as types of brain tumours other than glioma will inevitably be included. They will not be included in the main analysis; however, data from these will be stored for additional research into those less common tumours which can be challenging to diagnose from imaging alone.

At the time of surgery, neuro-navigation will be used to capture the location of each biopsy taken for diagnosis and further molecular characterisation within the TJBM study. This will aid with subsequent analysis of tumour molecular markers and heterogeneity in the context of radio-genomics – a recent development in cancer imaging for assessment of disease in the personalised medicine era employing machine learning and artificial intelligence approaches (32).

Early post-operative imaging for suspected HGG, including pre- and post-contrast T1-weighted MRI, is currently recommended within 72 hours of surgery to minimise the presence of non-neoplastic enhancement. This imaging will be captured to permit eventual assessment of extent of resection, or the volume of residual enhancing disease, important prognostic factors for glioma. Subsequent to this, any additional imaging for radiotherapy planning, including computerised tomography (CT), will be captured. Under the most recent response assessment in neuro-oncology (34) recommendations for HGG from 2017, this is recommended as the baseline for assessing treatment response (32).

All imaging will receive primary radiological reports at the local site as per current standard clinical practice. To deliver the imaging outcome requirements for clinical trials, centralised RANO reads will be delivered by a core group of UK Consultant neuro-radiologists with a subspecialty interest in neuro-oncology imaging from TJBM study sites. Radiotherapy planning data in the form of dose distribution maps and associated data will be integrated into the imaging database to permit subsequent analysis of spatiotemporal response patterns of different glioma-treatment combinations in light of received radiation dose.

Local radiological reporting will inform clinical decision making for individual patients. Central image analysis will provide resilient standardised assessment to meet internationally-accepted standards which are suitable for peer-reviewed publication and are accepted by major regulatory approval bodies. All relevant pre-operative and subsequent imaging will be identified for pseudonymisation and uploaded to the BRAIN MATRIX Imaging Platform in Edinburgh. This will be achieved through a dedicated online secure portal, with alternative secure online and physical transmission pathways available for redundancy. The accrued imaging data will form a core resource that will be leveraged for future imaging and clinical radiological research. The platform can also provide the basis for additional imaging studies within non-standard of care imaging and advanced techniques, which would be separately funded and detailed in their respective project documentation.

For peer review only

## Justification for tissue collection

Historical approaches to brain tumour tissue collection in formaldehyde and paraffin are not currently fully compatible with modern genomic technologies, which require frozen tissue. This is why the collection of fresh frozen (FF) material is essential for the TJBM study. Pairing with non-neoplastic, so-called germline DNA, is also essential for confident calling of somatic variants in the tumour. Germline DNA analysis will also provide novel data on genetic risk for glioma predisposition.

Blood plasma, where possible, will be collected to facilitate future analysis of cftDNA. This technology is still in its infancy; however, it is clear that non-invasive, real-time monitoring of tumour evolution will become feasible in the next 5-10 years (35). Similarly, where available for the patient, CSF will be submitted.

Post-mortem tissue banking via the CRUK PEACE and MRC Brain BioLink projects will allow us to study the glioma-brain interface, extent of spatial tumour heterogeneity, genomic signature of the treatment resistant tumour clones, and effect of treatments on normal brain. Systematic post-mortem brain banking for research into adult gliomas does not currently exist in the UK.

## Justification for molecular diagnostics

All TJBM study baseline diagnoses will follow the new WHO classification of tumours of the CNS from 2021 to achieve an integrated histological-molecular diagnosis for diffuse gliomas (10). This is achieved with a combination of immunohistochemical surrogate markers and – in most instances – targeted or panel sequencing for relevant hotspot mutations, or cytogenetics (7). Only in exceptional circumstances unbiased 'omics' approaches are used, such as the epigenomic 'Heidelberg Classifier' (34). Further, any diagnostic approach may differ between centres in the UK, making analyses of cohorts pooled from different sites difficult.

The new WHO 2021 classification represents a consensus opinion based on new insights into the molecular definition of brain tumours from cIMPACT-NOW. cIMPACT-NOW updates are not intended to supplant the existing WHO classification, but to provide possible guidelines for practicing diagnosticians and future WHO classification updates. It is clear from the first iterations of cIMPACT-NOW that any progress is driven by next generation ('omics') molecular analysis, not by standard or targeted analyses. This insight underpins the selection of molecular analytical tools for the TJBM platform, namely, combined paired (blood-tumour) WGS DNA analysis integrated with the epigenomic 'Heidelberg Classifier' (34). To achieve this, the TJBM study will build on the experience and UK infrastructure of the 100,000 genomes project led by GEL, which introduced WGS pathways into clinical practice. Early results from WGS in non-brain cancer patients suggest that virtually all patients could be mapped to existing or potential targeted therapies (36). Prospective large-scale paired WGS sequencing studies in diffuse glioma patients have not been done; however, experience from other brain tumours such as medulloblastoma, suggest that analysis of WGS data will provide new insights into glioma subtype diversity, including alterations in specific non-coding regulatory elements not evident from non-WGS genomic approaches (37). WGS data will capture all currently known relevant variants and uncover novel variants relevant for a better understanding of tumour evolution and response to treatment. WGS and epigenomic analyses are highly complementary genomic approaches (37): WGS will establish all potentially actionable mutations and epigenomic classification will establish an unbiased score for the precise classification of the glioma (34). Neither can be achieved with conventional targeted sequencing approaches. Importantly, the epigenomic classification by novel DNA methylation-based 'Heidelberg Classifier' has been shown to fundamentally alter clinical diagnoses and histological grades in >10% of biopsies, leading to changes in therapy (34). Moreover, the epigenomic array technology provides genomic copy number variant data, which in theory can also be

For peer review only - http://bmjopen.bmj.com/site/about/guidelines.xhtml

inferred from WGS data. However, pipelines for this type of analysis from WGS data are just evolving and it is predicted that comparative analysis of WGS and Illumina's EPIC BeadChip data will be bioinformatically highly valuable. Finally, WGS and the EPIC array raw data will form a unique source for researchers who will be able to access this data together with all clinical, imaging and histological data. Creating a relatively future-proof, quality-controlled research infrastructure is one of the main aims of the TJBM study, in addition to establishing feasibility of timely genomic diagnosis in the NHS setting.

the TJBM study is more than paired tumour/blood WGS and EPIC array analysis. As the former is being rolled out in the NHS in England, the BRAIN MATRIX molecular neuropathology team will work with GEL and other stakeholders (including industry) to explore the next-generation of tissue analytics (such as long-read sequencing and mass spectrometry). This will result in a unique, prospectively acquired dataset that will enable researchers to integrate data analysis across modalities and with outcomes and treatment response.

For peer review only

### Justification for patient outcome and quality of life

Clinical trials require prospective collection of clinical data to Good Clinical Practice (GCP) standards. This platform will develop the infrastructure for the collection of this, enabling streamlined patient recruitment into clinical trials. Evaluation of treatments and associated complications across patients will give an accurate measure of adverse events associated with current standard of care treatment in practice in the UK.

Maximising QoL for patients with diffuse glioma is important particularly given its poor prognosis; therefore, standardised measures of QoL will be collected. In adults these have been selected for their standardisation and ease of use and have been reviewed with input from Patient Public Involvement (PPI) representatives. It is intended that in the future integration with The Brain Tumour Charity's BRIAN project (REC Reference: 18/SC/0283) (19) will allow further understanding of patient reported outcome measures.

## Contributors

CW (Chief Investigator and first author): conception and design of the study, drafting and review of paper.

JS: trial management team leader, study design, drafting and review of paper.

AP: Study Biostatistician, study design, drafting and review of paper.

RM: senior trial coordinator, review of paper.

VW: principal investigator (Birmingham), review of paper.

UP: neuropathologist (Birmingham), study design, review of paper.

HB: patient and public representative, study design, review of paper.

JA: study design, review of paper.

RS: design of the study, review of paper.

GT: conception and design of the study, review of paper.

AM: conception and design of the study, review of paper.

OA: conception and design of the study, review of paper.

LB: Lead Biostatistician, design of study, drafting and review of paper.

## Funding

This is an Investigator-initiated and Investigator-led study funded by The Brain Tumour Charity (GN-000580) and Genomics England with funding from INNOVATE UK. This study has been adopted into the NIHR CRN Portfolio.

Staff at the CRCTU are also supported by core funding grants from Cancer Research UK (C22436/A25354). JA is funded by an NIHR Academic Clinical Lectureship, with previous funded from a Cancer Research UK Clinical Trials Fellowship.

This study was supported by Experimental Cancer Medicine Centres (ECMC) funding (Ref. 25127) and by the ECMC Network.

## Role of funders and sponsor

Neither the sponsor nor funders had any role in study design, data collection, data analysis, data interpretation or writing of the report. The corresponding author had full access to all the data in the study and had final responsibility for the decision to submit for publication.

## Competing interests

All other authors declare no competing interests.

## Acknowledgements

We would like to thank our PPI representative, Peter Buckle who, with Helen Bulbeck, has been instrumental in making sure the patients' voice has been heard and incorporated into this study protocol. In addition, we thank staff past and present from the CRCTU, University of Birmingham including Clive Stubbs, Richard Fox, Dr Sarah Bowden, Prof. Christina Yap, Dr Siân Lax and Dr Louisa Jeffery, and Hannah Brooks at the University of Oxford for contributions to the protocol and paper.

## References

1. Ostrom QT, Gittleman H, Fulop J, Liu M, Blanda R, Kromer C, et al. CBTRUS Statistical Report: Primary Brain and Central Nervous System Tumors Diagnosed in the United States in 2008-2012. Neuro Oncol. 2015;17 Suppl 4:iv1-iv62.

2. Cancer Research UK. Brain, other CNS and intracranial tumour statistics [Available from: https://www.cancerresearchuk.org/health-professional/cancer-statistics/statistics-by-cancer-type/brain-other-cns-and-intracranial-tumours.

3. Rouse C, Gittleman H, Ostrom QT, Kruchko C, Barnholtz-Sloan JS. Years of potential life lost for brain and CNS tumors relative to other cancers in adults in the United States, 2010. Neuro Oncol. 2016;18(1):70-7.

4. Rachet B, Mitry E, Quinn MJ, Cooper N, Coleman MP. Survival from brain tumours in England and Wales up to 2001. Br J Cancer. 2008;99 Suppl 1:S98-101.

For peer review only

5. Louis DN, Perry A, Reifenberger G, von Deimling A, Figarella-Branger D, Cavenee WK, et al. The 2016 World Health Organization Classification of Tumors of the Central Nervous System: a summary. Acta Neuropathol. 2016;131(6):803-20.

6. Sanai N, Chang S, Berger MS. Low-grade gliomas in adults. J Neurosurg. 2011;115(5):948-65.

7. Stupp R, Mason WP, van den Bent MJ, Weller M, Fisher B, Taphoorn MJB, et al. Radiotherapy plus Concomitant and Adjuvant Temozolomide for Glioblastoma. New England Journal of Medicine. 2005;352(10):987-96.

8. Weller M, Wick W, Aldape K, Brada M, Berger M, Pfister SM, et al. Glioma. Nature Reviews Disease Primers. 2015;1(1):15017.

9. The International Society of Neuropathology. cIMPACT-NOW 2019 [Available from: http://www.intsocneuropathol.com/2019/03/cimpact-now/.

10. Louis DN, Perry A, Wesseling P, Brat DJ, Cree IA, Figarella-Branger D, et al. The 2021 WHO Classification of Tumors of the Central Nervous System: a summary. Neuro-Oncology. 2021;23(8):1231-51.

11. 100,000 Genomes Pilot on Rare-Disease Diagnosis in Health Care — Preliminary Report. New England Journal of Medicine. 2021;385(20):1868-80.

12. UK Biobank 2022 [Available from: https://www.ukbiobank.ac.uk/.

13. MRC Brain Banks Network [Available from: https://directory.biobankinguk.org/Profile/Biobank/GBR-1-34.

14. The PEACE (Posthumous Evaluation of Advanced Cancer Environment) Study 2016 [Available from: https://clinicaltrials.gov/ct2/show/NCT03004755.

15. Aldape K, Amin SB, Ashley DM, Barnholtz-Sloan JS, Bates AJ, Beroukhim R, et al. Glioma through the looking GLASS : Molecular evolution of diffuse gliomas and the Glioma Longitudinal Analysis Consortium. Neuro-oncology. 2018;20(7):11.

16. EURACAN. European Network for Rare adult solid Cancer [Available from: http://euracan.ern-net.eu/.

17. Medical Research Council. Tariffs for Brain Tissue 2021 [Available from: https://webarchive.nationalarchives.gov.uk/ukgwa/20210903230131/https://mrc.ukri.org/research/facilities-and-resources-for-researchers/brain-banks/tariffs-for-brain-tissue/.

18. Chan A-W, Tetzlaff JM, Gøtzsche PC, Altman DG, Mann H, Berlin JA, et al. SPIRIT 2013 explanation and elaboration: guidance for protocols of clinical trials. BMJ : British Medical Journal. 2013;346:e7586.

19. The Brain Tumour Charity. BRIAN [Available from: https://www.thebraintumourcharity.org/living-with-a-brain-tumour/brian/.

20. Lyden P, Brott T, Tilley B, Welch KM, Mascha EJ, Levine S, et al. Improved reliability of the NIH Stroke Scale using video training. NINDS TPA Stroke Study Group. Stroke. 1994;25(11):2220-6.

21. Group TE. EuroQol - a new facility for the measurement of health-related quality of life. Health Policy. 1990;16(3):199-208.

22. Aaronson NK, Ahmedzai S, Bergman B, Bullinger M, Cull A, Duez NJ, et al. The European Organization for Research and Treatment of Cancer QLQ-C30: A Quality-of-Life Instrument for Use in International Clinical Trials in Oncology. Journal of the National Cancer Institute. 1993;85(5):365-76.

23. Rogers SN, El-Sheikha J, Lowe D. The development of a Patients Concerns Inventory (PCI) to help reveal patients concerns in the head and neck clinic. Oral Oncol. 2009;45(7):555-61.

24. Guy W, National Institute of Mental H, Psychopharmacology Research B, Early Clinical Drug Evaluation P. ECDEU assessment manual for psychopharmacology. Rockville, Md.: U.S. Dept. of Health, Education, and Welfare, Public Health Service, Alcohol, Drug Abuse, and Mental Health Administration, National Institute of Mental Health, Psychopharmacology Research Branch, Division of Extramural Research Programs; 1976.

25. Busner J, Targum SD. The clinical global impressions scale: applying a research tool in clinical practice. Psychiatry (Edgmont). 2007;4(7):28-37.

For peer review only - http://bmjopen.bmj.com/site/about/guidelines.xhtml

26. Chukwueke UN, Wen PY. Use of the Response Assessment in Neuro-Oncology (RANO) criteria in clinical trials and clinical practice. CNS Oncol. 2019;8(1):CNS28-CNS.

27. Heidelberg Classifier. The platform for next generation neuropathology [Available from: www.molecularneuropathology.org/mnp.

28. S:CORT: Colorectal Cancer Detection & Diganosis Novel Treatments 2022 [Available from: https://www.cancer.ox.ac.uk/research/networks/scort.

29. cBioPortal for Cancer Genomics [Available from: https://www.cbioportal.org/.

30. National Institute for Health and Care Excellence. Brain tumours (primary) and brain metastases in over 16s 2018 [updated 29-Apr-2022. Available from: https://www.nice.org.uk/guidance/ng99.

31. British Society of Neuroradiologists. Core imaging protocol for brain tumours 2018 [Available from: https://bsnr.org.uk/wp-content/uploads/2021/11/bsnrstandardsbraintumour.pdf.

32. Ellingson BM. Radiogenomics and Imaging Phenotypes in Glioblastoma: Novel Observations and Correlation with Molecular Characteristics. Current Neurology and Neuroscience Reports. 2014;15(1):506.

33. Cancer Therapy Evaluation Program. Common Terminology Criteria for Adverse Events (CTCAE), Version 4.03 2010 [Available from: https://ctep.cancer.gov/protocoldevelopment/electronic_applications/ctc.htm#ctc_40.

34. Capper D, Jones DTW, Sill M, Hovestadt V, Schrimpf D, Sturm D, et al. DNA methylation-based classification of central nervous system tumours. Nature. 2018;555:469.

35. De Rubis G, Rajeev Krishnan S, Bebawy M. Liquid Biopsies in Cancer Diagnosis, Monitoring, and Prognosis. Trends in Pharmacological Sciences. 2019;40(3):172-86.

36. Schuh A, Dreau H, Knight SJL, Ridout K, Mizani T, Vavoulis D, et al. Clinically actionable mutation profiles in patients with cancer identified by whole-genome sequencing. Cold Spring Harb Mol Case Stud. 2018;4(2):a002279.

37. Northcott PA, Buchhalter I, Morrissy AS, Hovestadt V, Weischenfeldt J, Ehrenberger T, et al. The whole-genome landscape of medulloblastoma subtypes. Nature. 2017;547:311.

# Figure Legends

## Figure 1: Overview of the Tessa Jowell BRAIN MATRIX Programme

The Tessa Jowell BRAIN MATRIX Platform (TJBM) study will collect and integrate clinical, pathological, advanced molecular, imaging, quality of life, treatment and outcome data. The platform may provide data directly or support identification of eligible patients to clinical trials, within and outside the TJBM study programme. If eligible, patients may be enrolled in multiple add-on studies. Through consent and with strong governance processes, anonymised or pseudonymised data may be shared with other relevant organisations or studies within and outside the programme.

## Figure 2: The Tessa Jowell BRAIN MATRIX Platform study schema

The study schema for the Tessa Jowell BRAIN MATRIX Platform study.

For peer review only

## Figure 3: The Tessa Jowell BRAIN MATRIX Platform study sample and data flow pathways

The sample and data flow pathways within the Tessa Jowell BRAIN MATRIX Platform (TJBM) study.

*TJBM sites in England are encouraged to route all samples through their local NHS GMS GLH, however, if this pathway is not yet activated or GEL consent cannot be obtained, the TJBM Study Office can facilitate the processing of samples through an alternative NHS GMS GLH or via the GEL Research pathway.

GEL, Genomics England; GLH, Genomic Laboratory Hub; GMS, Genomic Medicine Service; GTAB, Genomics Tumour Advisory Board; NHS, National Health Service; WGS, Whole Genome Sequencing.

## Additional Files

### Supplementary Appendix 1: Tessa Jowell BRAIN MATRIX Platform Study Investigators & Committee Membership

Principal Investigators, Study Management Group (SMG), Executive Oversight Committee (EOC) and Scientific Advisory Board (SAB).

### Supplementary Appendix 2: The Tessa Jowell BRAIN MATRIX Platform study SPIRIT Checklist

A completed Standard Protocol Items: Recommendations for Intervention Trials (SPIRIT) checklist for the Tessa Jowell BRAIN MATRIX Platform study protocol.

### Supplementary Appendix 3: The Tessa Jowell BRAIN MATRIX Platform study World Health Organization Trial Registration Data Set

The World Health Organization (WHO) trial registration data set for the Tessa Jowell BRAIN MATRIX Platform study.

### Supplementary Appendix 4: The Tessa Jowell BRAIN MATRIX Platform study informed consent form

The informed consent form for the Tessa Jowell BRAIN MATRIX Platform study.

For peer review only

## Supplementary Appendix 5: The Tessa Jowell BRAIN MATRIX Platform study lay summary and patient information sheet

The lay summary and patient information sheet for the Tessa Jowell BRAIN MATRIX Platform study.

## Supplementary Appendix 6: The Tessa Jowell BRAIN MATRIX Platform study schedule of events

Patient schedule of events for the Tessa Jowell BRAIN MATRIX Platform study.

## Supplementary Appendix 7: Definition of adverse events

Definitions of adverse events used for the Tessa Jowell BRAIN MATRIX Platform study.

For peer review only

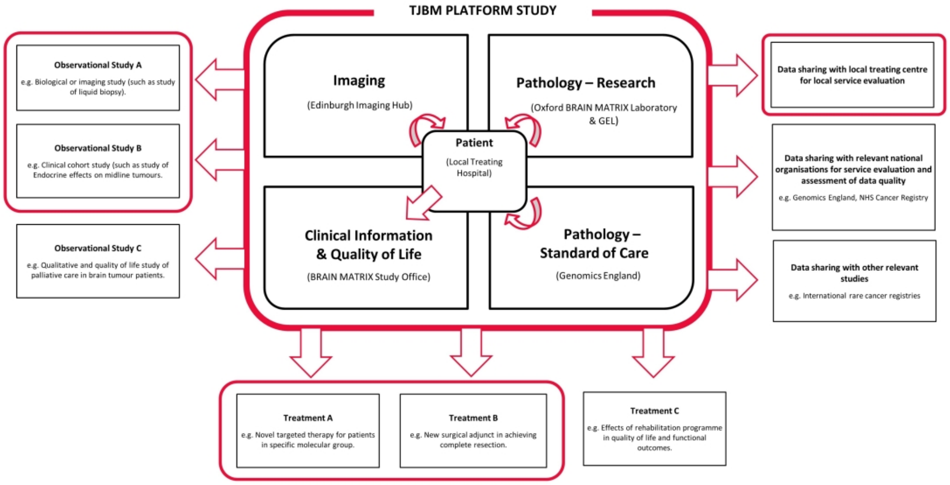


338x190mm (300 x 300 DPI)

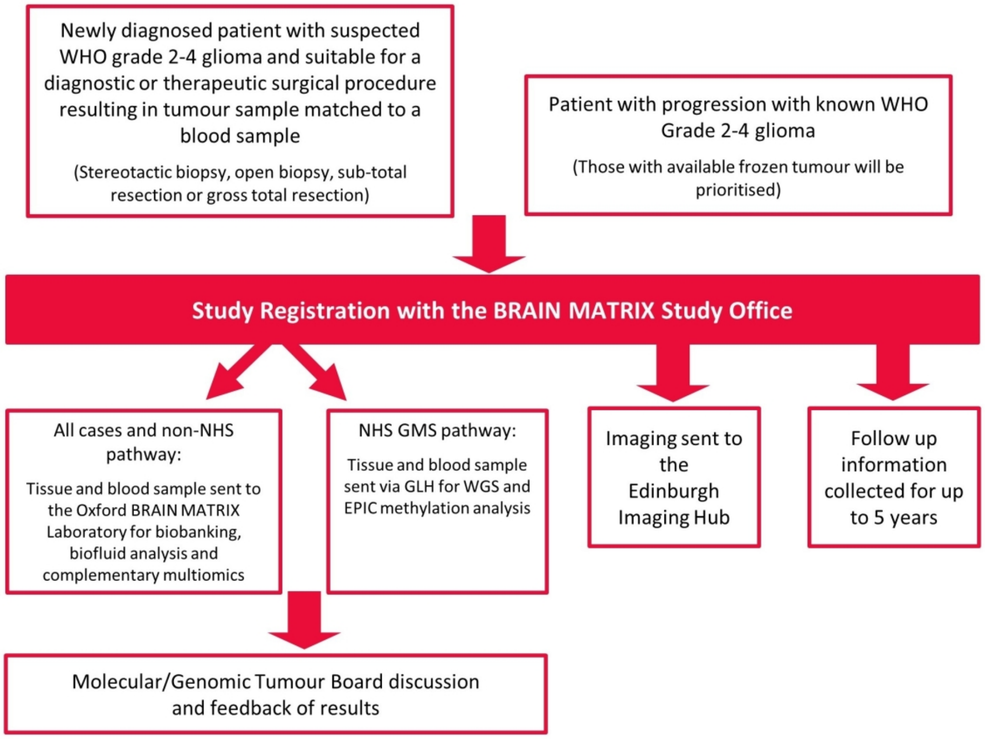


176x133mm (300 x 300 DPI)

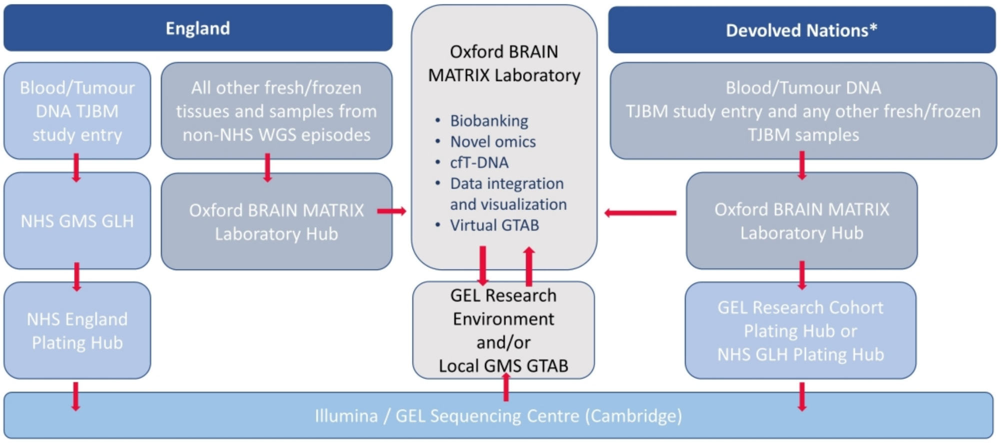


323x146mm (300 x 300 DPI)

# Supplementary Appendix 1: Tessa Jowell BRAIN MATRIX Platform Study Investigators & Committee Membership

**Principal Investigators**

- Dr Victoria Wykes - Queen Elizabeth Hospital, Birmingham
- Prof. Keyoumars Ashkan - King's College Hospital, London
- Prof. Michael Jenkinson - The Walton Centre, Liverpool
- Prof. Susan Short - St James's University Hospital. Leeds
- Dr Paul Brennan - NHS Lothian
- Dr Richard Mair - Addenbrooke's Hospital, Cambridge
- Dr Stuart Smith - Queens Medical Centre, Nottingham
- Prof. Anthony Chalmers - Greater Glasgow and Clyde
- Dr David Coope - Salford Royal Hospital, Manchester
- Dr Catherine McBain - The Christie, Manchester
- Dr Clare Hobbs - John Radcliffe Hospital, Oxford

**Study Management Group (SMG)**

- Professor Colin Watts (Chief Investigator) - Institute of Cancer and Genomic Sciences (ICGS), University of Birmingham
- Professor Olaf Ansorge - University of Oxford
- Dr Ute Pohl - University Hospitals Birmingham
- Professor Adam Waldman - University of Edinburgh
- Dr Gerard Thompson - University of Edinburgh
- Dr John Apps - University of Birmingham
- Dr Victoria Wykes - University of Birmingham
- Professor Lucinda Billingham – CRCTU, University of Birmingham
- Dr Rowena Sharpe – CRCTU, University of Birmingham
- Amit Patel - CRCTU, University of Birmingham
- Dr Joshua Savage - CRCTU, University of Birmingham
- Rhys Mant - CRCTU, University of Birmingham
- Dr Louisa Jeffery - CRCTU, University of Birmingham
- Hannah Brooks – University of Oxford
- Peter Buckle (PPI Representative)
- Dr Helen Bulbeck (PPI Representative) - brainstrust

**Executive Oversight Committee (EOC)**

- Professor Tim Maughan (Chair)– Professor of Clinical Oncology, CRUK/MRC Institute for Radiation Oncology, University of Oxford



For peer review only - http://bmjopen.bmj.com/site/about/guidelines.xhtml

- Professor David Cameron – Clinical Director of the Cancer Research UK Edinburgh Centre, University of Edinburgh
- Dr Helen Campbell – Portfolio Manager for Department of Health and Social Care Research Networks, Clinical Research Facilities, and Cancer Research, University of Exeter
- Dr Mark Gilbert – Chair of the Neuro-Oncology Branch, Centre for Cancer Research, National Cancer Institute
- Professor Richard Gilbertson – Director of CRUK Cambridge Centre
- Professor Max Parmar – Director of the MRC Clinical Trials Unit, UCL
- Lord James O'Shaughnessy – Member of House of Lords
- Professor Steven Pollard – Group Leader, MRC Centre for Regenerative Medicine and Edinburgh Cancer Research Centre, University of Edinburgh
- Jess Mills – Co-Founder and Special Advisor, Tessa Jowell Brain Cancer Mission

**Scientific Advisory Board (SAB)**

- Professor Ruth Plummer (Chair) - Professor of Experimental Cancer Medicine, Newcastle University
- Professor Neil Carragher - Professor of Drug Discovery, University of Edinburgh
- Professor Anthony Chalmers - Chair of Clinical Oncology, University of Glasgow
- Will Jones - Chief Executive, brainstrust
- Dr Juanita Lopez - Consultant Medical Oncologist, The Royal Marsden NHSFT
- Professor Whitney Pope – Director, Brain Tumour Imaging, UCLA
- Professor Thomas Wurdinger - Professor of Neurosurgery, Amsterdam UMC
- Professor Christina Yap - Professor of Clinical Trials Biostatistics, ICR
- Professor Patrick Wen - Professor of Neurology, Harvard Medical School
- Professor Roel Verhaak - Professor and Associate Director for Computational Biology, The Jackson Laboratory for Genomic Medicine



For peer review only - http://bmjopen.bmj.com/site/about/guidelines.xhtml

# Reporting checklist for protocol of a clinical trial.

Based on the SPIRIT guidelines.

## Instructions to authors

Complete this checklist by entering the page numbers from your manuscript where readers will find each of the items listed below.

Your article may not currently address all the items on the checklist. Please modify your text to include the missing information. If you are certain that an item does not apply, please write "n/a" and provide a short explanation.

Upload your completed checklist as an extra file when you submit to a journal.

In your methods section, say that you used the SPIRITreporting guidelines, and cite them as:

Chan A-W, Tetzlaff JM, Gøtzsche PC, Altman DG, Mann H, Berlin J, Dickersin K, Hróbjartsson A, Schulz KF, Parulekar WR, Krleža-Jerić K, Laupacis A, Moher D. SPIRIT 2013 Explanation and Elaboration: Guidance for protocols of clinical trials. BMJ. 2013;346:e7586

For peer review only

| | | Reporting Item | Page Number |
|---|---|---|---|
| **Administrative information** | | | |
| Title | #1 | Descriptive title identifying the study design, population, interventions, and, if applicable, trial acronym | 1 |
| Trial registration | #2a | Trial identifier and registry name. If not yet registered, name of intended registry | 2 |
| Trial registration: data set | #2b | All items from the World Health Organization Trial Registration Data Set | Appendix 3 |
| Protocol version | #3 | Date and version identifier | 12 |
| Funding | #4 | Sources and types of financial, material, and other support | 16 |

| | | | |
|---|---|---|---|
| Roles and responsibilities: contributorship | #5a | Names, affiliations, and roles of protocol contributors | 1 + 15-16 |
| Roles and responsibilities: sponsor contact information | #5b | Name and contact information for the trial sponsor | 11 |
| Roles and responsibilities: sponsor and funder | #5c | Role of study sponsor and funders, if any, in study design; collection, management, analysis, and interpretation of data; writing of the report; and the decision to submit the report for publication, including whether they will have ultimate authority over any of these activities | 16 |
| Roles and responsibilities: committees | #5d | Composition, roles, and responsibilities of the coordinating centre, steering committee, endpoint adjudication committee, data management team, and other individuals or groups overseeing the trial, if applicable (see Item 21a for data monitoring committee) | 11 |
| **Introduction** | | | |
| Background and rationale | #6a | Description of research question and justification for undertaking the trial, including summary of relevant studies (published and unpublished) examining benefits and harms for each intervention | 3-4 |
| Background and rationale: choice of comparators | #6b | Explanation for choice of comparators | n/a |
| Objectives | #7 | Specific objectives or hypotheses | 3-4 |
| Trial design | #8 | Description of trial design including type of trial (eg, parallel group, crossover, factorial, single group), allocation ratio, and framework (eg, superiority, equivalence, non-inferiority, exploratory) | 4 |
| **Methods: Participants,** | | | |

For peer review only

| | | | |
|---|---|---|---|
| **interventions, and outcomes** | | | |
| Study setting | #9 | Description of study settings (eg, community clinic, academic hospital) and list of countries where data will be collected. Reference to where list of study sites can be obtained | 4 |
| Eligibility criteria | #10 | Inclusion and exclusion criteria for participants. If applicable, eligibility criteria for study centres and individuals who will perform the interventions (eg, surgeons, psychotherapists) | 5 |
| Interventions: description | #11a | Interventions for each group with sufficient detail to allow replication, including how and when they will be administered | n/a |
| Interventions: modifications | #11b | Criteria for discontinuing or modifying allocated interventions for a given trial participant (eg, drug dose change in response to harms, participant request, or improving / worsening disease) | n/a |
| Interventions: adherance | #11c | Strategies to improve adherence to intervention protocols, and any procedures for monitoring adherence (eg, drug tablet return; laboratory tests) | n/a |
| Interventions: concomitant care | #11d | Relevant concomitant care and interventions that are permitted or prohibited during the trial | n/a |
| Outcomes | #12 | Primary, secondary, and other outcomes, including the specific measurement variable (eg, systolic blood pressure), analysis metric (eg, change from baseline, final value, time to event), method of aggregation (eg, median, proportion), and time point for each outcome. Explanation of the clinical relevance of chosen efficacy and harm outcomes is strongly recommended | 6 |
| Participant timeline | #13 | Time schedule of enrolment, interventions (including any run-ins and washouts), assessments, and visits for participants. A schematic diagram is highly recommended (see Figure) | Appendix 6 |

For peer review only - http://bmjopen.bmj.com/site/about/guidelines.xhtml

| | | | |
|---|---|---|---|
| Sample size | #14 | Estimated number of participants needed to achieve study objectives and how it was determined, including clinical and statistical assumptions supporting any sample size calculations | 6-7 |
| Recruitment | #15 | Strategies for achieving adequate participant enrolment to reach target sample size | n/a |
| **Methods: Assignment of interventions (for controlled trials)** | | | |
| Allocation: sequence generation | #16a | Method of generating the allocation sequence (eg, computer-generated random numbers), and list of any factors for stratification. To reduce predictability of a random sequence, details of any planned restriction (eg, blocking) should be provided in a separate document that is unavailable to those who enrol participants or assign interventions | n/a |
| Allocation concealment mechanism | #16b | Mechanism of implementing the allocation sequence (eg, central telephone; sequentially numbered, opaque, sealed envelopes), describing any steps to conceal the sequence until interventions are assigned | n/a |
| Allocation: implementation | #16c | Who will generate the allocation sequence, who will enrol participants, and who will assign participants to interventions | 5 |
| Blinding (masking) | #17a | Who will be blinded after assignment to interventions (eg, trial participants, care providers, outcome assessors, data analysts), and how | n/a |
| Blinding (masking): emergency unblinding | #17b | If blinded, circumstances under which unblinding is permissible, and procedure for revealing a participant's allocated intervention during the trial | n/a |
| **Methods: Data collection, management, and analysis** | | | |

For peer review only

| | | | |
|---|---|---|---|
| Data collection plan | #18a | Plans for assessment and collection of outcome, baseline, and other trial data, including any related processes to promote data quality (eg, duplicate measurements, training of assessors) and a description of study instruments (eg, questionnaires, laboratory tests) along with their reliability and validity, if known. Reference to where data collection forms can be found, if not in the protocol | 10 |
| Data collection plan: retention | #18b | Plans to promote participant retention and complete follow-up, including list of any outcome data to be collected for participants who discontinue or deviate from intervention protocols | n/a |
| Data management | #19 | Plans for data entry, coding, security, and storage, including any related processes to promote data quality (eg, double data entry; range checks for data values). Reference to where details of data management procedures can be found, if not in the protocol | 10 |
| Statistics: outcomes | #20a | Statistical methods for analysing primary and secondary outcomes. Reference to where other details of the statistical analysis plan can be found, if not in the protocol | 6-8 |
| Statistics: additional analyses | #20b | Methods for any additional analyses (eg, subgroup and adjusted analyses) | 8 |
| Statistics: analysis population and missing data | #20c | Definition of analysis population relating to protocol non-adherence (eg, as randomised analysis), and any statistical methods to handle missing data (eg, multiple imputation) | n/a |
| **Methods: Monitoring** | | | |
| Data monitoring: formal committee | #21a | Composition of data monitoring committee (DMC); summary of its role and reporting structure; statement of whether it is independent from the sponsor and competing interests; and reference to where further details about its charter can be found, if not in the | n/a |

For peer review only

| | | | |
|---|---|---|---|
| | | protocol. Alternatively, an explanation of why a DMC is not needed | |
| Data monitoring: interim analysis | #21b | Description of any interim analyses and stopping guidelines, including who will have access to these interim results and make the final decision to terminate the trial | 6-7 |
| Harms | #22 | Plans for collecting, assessing, reporting, and managing solicited and spontaneously reported adverse events and other unintended effects of trial interventions or trial conduct | 10 |
| Auditing | #23 | Frequency and procedures for auditing trial conduct, if any, and whether the process will be independent from investigators and the sponsor | n/a |
| **Ethics and dissemination** | | | |
| Research ethics approval | #24 | Plans for seeking research ethics committee / institutional review board (REC / IRB) approval | 12 |
| Protocol amendments | #25 | Plans for communicating important protocol modifications (eg, changes to eligibility criteria, outcomes, analyses) to relevant parties (eg, investigators, REC / IRBs, trial participants, trial registries, journals, regulators) | 12 |
| Consent or assent | #26a | Who will obtain informed consent or assent from potential trial participants or authorised surrogates, and how (see Item 32) | 5 |
| Consent or assent: ancillary studies | #26b | Additional consent provisions for collection and use of participant data and biological specimens in ancillary studies, if applicable | 5-6 |
| Confidentiality | #27 | How personal information about potential and enrolled participants will be collected, shared, and maintained in order to protect confidentiality before, during, and after the trial | 11-12 |
| Declaration of interests | #28 | Financial and other competing interests for principal investigators for the overall trial and each study site | 16 |

| | | | |
|---|---|---|---|
| Data access | #29 | Statement of who will have access to the final trial dataset, and disclosure of contractual agreements that limit such access for investigators | 11 |
| Ancillary and post trial care | #30 | Provisions, if any, for ancillary and post-trial care, and for compensation to those who suffer harm from trial participation | n/a |
| Dissemination policy: trial results | #31a | Plans for investigators and sponsor to communicate trial results to participants, healthcare professionals, the public, and other relevant groups (eg, via publication, reporting in results databases, or other data sharing arrangements), including any publication restrictions | 12 |
| Dissemination policy: authorship | #31b | Authorship eligibility guidelines and any intended use of professional writers | 12 |
| Dissemination policy: reproducible research | #31c | Plans, if any, for granting public access to the full protocol, participant-level dataset, and statistical code | 11 |
| **Appendices** | | | |
| Informed consent materials | #32 | Model consent form and other related documentation given to participants and authorised surrogates | Appendices 4 and 5 |
| Biological specimens | #33 | Plans for collection, laboratory evaluation, and storage of biological specimens for genetic or molecular analysis in the current trial and for future use in ancillary studies, if applicable | 8-9 |

None The SPIRIT Explanation and Elaboration paper is distributed under the terms of the Creative Commons Attribution License CC-BY-NC. This checklist can be completed online using https://www.goodreports.org/, a tool made by the EQUATOR Network in collaboration with Penelope.ai

For peer review only

# Supplementary Appendix 3: The Tessa Jowell BRAIN MATRIX Platform study World Health Organization Trial Registration Data Set

| Data category | Information |
|---|---|
| Primary registry and trial identifying number | ClinicalTrials.gov<br><br>NCT04274283 |
| Date of registration in primary registry | 18-Feb-2020 |
| Secondary identifying numbers | ISRCTN 14218060 |
| Source(s) of monetary or material support | The Brain Tumour Charity<br><br>Genomics England<br><br>INNOVATE UK |
| Primary sponsor | University of Birmingham |
| Secondary sponsor(s) | n/a |
| Contact for public queries | Dr Joshua Savage: brainmatrix@trials.bham.ac.uk |
| Contact for scientific queries | Prof. Colin Watts: c.watts.2@bham.ac.uk |
| Public title | The Tessa Jowell BRAIN MATRIX Platform Study |
| Scientific title | A British Feasibility Study of Molecular Stratification and Targeted Therapy to Optimize the Clinical Management of Patients With Glioma by Enhancing Clinical Outcomes, Reducing Avoidable Toxicity, Improving |



For peer review only - http://bmjopen.bmj.com/site/about/guidelines.xhtml

| Data category | Information |
|---|---|
| | Management of Post-operative Residual & Recurrent Disease and Improving Survivorship |
| Countries of recruitment | UK |
| Health condition(s) or problem(s) studied | Newly diagnosed suspected, or progressive with known, WHO Grade 2-4 glioma |
| Intervention(s) | None |
| Key inclusion and exclusion criteria: | Ages eligible for study: ≥16 years<br>Sexes eligible for study: both<br>Accepts healthy volunteers: no |
| | Inclusion criteria: Newly diagnosed suspected, or progressive with known, WHO Grade 2-4 glioma |
| | Exclusion criteria: Primary spinal cord tumours, active treatment of other malignancy |
| Study type | Observational |
| | Primary purpose: Feasibility of establishing an integrated histological-molecular diagnosis of glioblastoma |
| Date of first enrolment | 14-Dec-2020 |
| Target sample size | 1000 |
| Recruitment status | Open |
| Primary outcome(s) | • Time (from biopsy) to integrated histological–molecular diagnosis (TTMD) using whole genome sequencing and epigenomic classification |



For peer review only - http://bmjopen.bmj.com/site/about/guidelines.xhtml

| Data category | Information |
|---|---|
| Key secondary outcome(s) | • Time to completion of each node of tissue and imaging pathway.<br>• Tumour and biological sample(s) quality control (QC) status.<br>• Imaging QC status.<br>• Inter-rater agreement of RANO assessments. |

For peer review only



For peer review only - http://bmjopen.bmj.com/site/about/guidelines.xhtml

*To be printed on hospital headed paper*

TESSA JOWELL BRAIN MATRIX

**A BRitish feasibility study of molecular stratification and targeted therapy to optimize the clinical mAnagement of patIeNts with glioMA by enhancing clinical ouTcomes, Reducing avoIdable toXicity, improving management of post-operative residual & recurrent disease and improving survivorship**

## Platform Study

# Informed Consent Form

| | | | |
|---|---|---|---|
| **Site:** | ______________________ | **Patient's Study Number:** | ☐☐☐☐ |
| **Principal Investigator:** | ______________________ | | |

**Sponsor's Protocol Number: RG_18-258**

**Please initial each box**

1. I confirm that I have read and understand the Patient Information Sheet (version .................. dated.........................) for the above study. I have had the opportunity to consider the information, ask questions and have had these answered satisfactorily. ☐

2. I understand that my participation is voluntary and that I am free to withdraw at any time without giving any reason, without my medical care or legal rights being affected. ☐

CRCTU-ICF-QCD-001, version 2.0a



For peer review only - http://bmjopen.bmj.com/site/about/guidelines.xhtml

3. I give my permission for my initials, date of birth, hospital number, NHS/CHI number, Genomics England Referral ID number, histopathology numbers, and radiology numbers to be given to the BRAIN MATRIX Study Office when I am registered to the study as well as a copy of this consent form. I understand that copies of the consent form may be forwarded to other healthcare professionals to prove that I am taking part in the study. I also give permission for my information to be shared with other hospitals involved in my care and with the National Cancer Registration Service. ☐

4. I understand that relevant sections of my medical notes and data collected during the study may be looked at by individuals from the BRAIN MATRIX Study Office, regulatory authorities, Sponsors and/or NHS bodies, where it is relevant to my taking part in this research. I understand that this information will be held in a confidential manner. I give permission for these individuals to have access to my records. ☐

5. I agree to the collection of blood samples, or any other biofluid samples, from me, and consent for these samples to be analysed (including genetic analysis) as part of research associated with this study, to be used in biobanking and for future ethically approved studies. ☐

6. I agree to the collection of tissue from any of my previous biopsies and future tumour surgeries (including "surgical access tissue") and consent for these samples to be analysed (including genetic analysis) as part of research associated with this study, to be used in biobanking and for future ethically approved studies. ☐

7. I understand that identifiable data from the study may be shared with other institutions involved in BRAIN MATRIX (University of Edinburgh, University of Oxford, Genomics England, BRIAN databank run by The Brain Tumour Charity). ☐

8. Anonymised linked data may also be provided to other 3rd parties (e.g. other academic institutions, pharmaceutical companies and QMENTA) for research and safety monitoring purposes and may be placed in recognised medical research data repositories, in accordance with best research practice. ☐

9. I agree to my GP being informed of my participation in this study and to my GP being sent a copy of this Patient Information Sheet. ☐

10. I agree to the collection of different aspects of my health data from the NHS and other Department of Health organisations, in addition to my medical records. The collection and analysis of my health data for research will continue across my entire lifetime and beyond. ☐

11. I agree to take part in the above study. ☐



For peer review only - http://bmjopen.bmj.com/site/about/guidelines.xhtml

**The following are <u>optional</u> and will not affect entry into the study, please initial in the relevant box:**

| | No | Yes |
|---|---|---|
| If in the event that an abnormality that might affect other family members is uncovered during genetic testing for the study, I would like my doctor to be informed and to be referred to a genetic counsellor if appropriate. | | |
| I would like to receive a copy of the lay summary results at the end of the study. | | |
| I agree that my doctor, in consultation with the BRAIN MATRIX Study Office, together with Genomics England and other NHS staff, can contact me if the data or samples reveal any clinical trials or other research that I might benefit from. If something is relevant to me, there is a process by which this will be shared with my NHS clinical team. | | |

| **Name of patient** | **Date** | **Signature** |
|---|---|---|
| **Name of person taking consent**<br>You must have signed the<br>Site Signature & Delegation Log | **Date** | **Signature** |

For peer review only



For peer review only - http://bmjopen.bmj.com/site/about/guidelines.xhtml

*To be printed on local hospital headed paper*

# TESSA JOWELL BRAIN MATRIX

**A BRitish feasibility study of molecular stratification and targeted therapy to optimize the clinical mAnagement of patIeNts with glioMA by enhancing clinical ouTcomes, Reducing avoIdable toXicity, improving management of post-operative residual & recurrent disease and improving survivorship**

## Platform Study

The **Tessa Jowell BRAIN MATRIX** is a programme of work, the principal purpose of which is to improve the knowledge of, and treatment for, glioma, a type of brain tumour.

Brain tumours arise due to changes in the DNA and other molecules in cells of the brain. Different types of gliomas can have different changes and these can be used to determine a precise 'molecular diagnosis'. The ultimate goal for the Tessa Jowell BRAIN MATRIX is to learn how to use these molecular changes to more precisely determine what exact type of tumour patients have, and to identify, decide and test whether specific 'targeted' treatments could improve the survival and/or quality of life of patients with brain tumours.

The Tessa Jowell BRAIN MATRIX Platform Study forms the backbone of this programme. You have been given this information because you either have an existing brain tumour, or have recently been diagnosed with a brain tumour, likely to be a glioma.

In the platform study we aim to develop the infrastructure to provide rapid and accurate molecular diagnosis and the infrastructure to deliver clinical trials of new therapies in the future.

**The diagram below shows a summary of the Tessa Jowell BRAIN MATRIX Programme:**

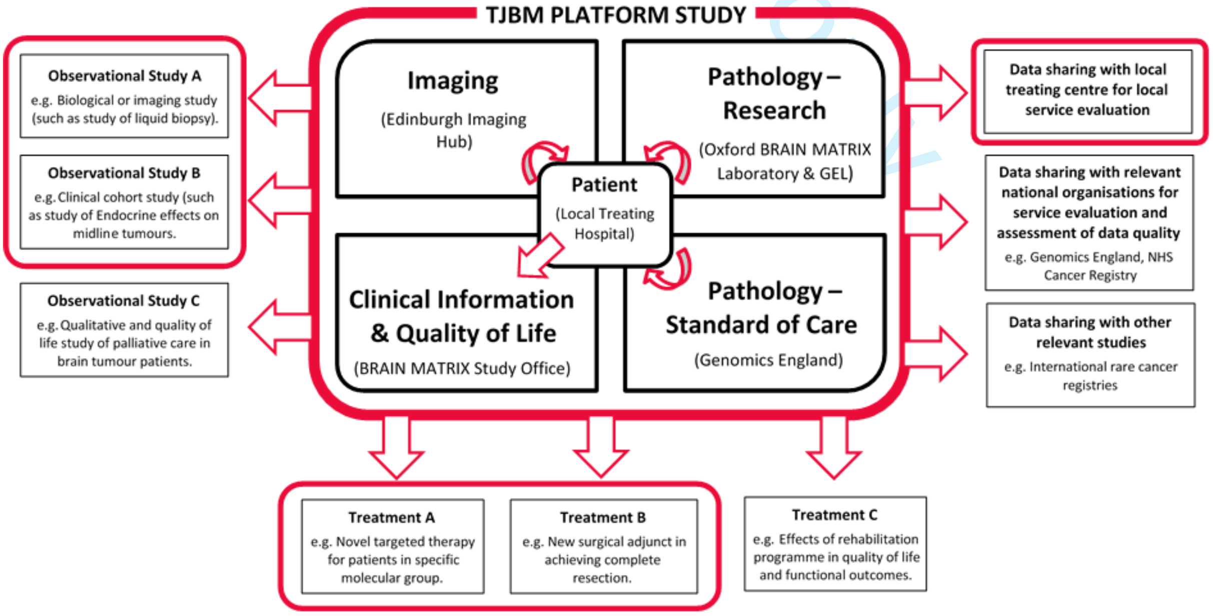


For further information about the Tessa Jowell BRAIN MATRIX Platform Study, please ask your doctor.


For peer review only - http://bmjopen.bmj.com/site/about/guidelines.xhtml

*To be printed on local hospital headed paper*

TESSA JOWELL BRAIN MATRIX

**A BRitish feasibility study of molecular stratification and targeted therapy to optimize the clinical mAnagement of patIeNts with glioMA by enhancing clinical ouTcomes, Reducing avoIdable toXicity, improving management of post-operative residual & recurrent disease and improving survivorship**

## Platform Study

# Patient Information Sheet

*To be used for patients over 16 years of age considering entering the Platform Study*

## We invite you to take part in a clinical study

We would like to invite you to take part in a non-commercial clinical study run by the University of Birmingham, supported by The Brain Tumour Charity.

Before you decide whether you would like to participate in this study, we would like you to understand:

- Why the research is being done.
- What it would involve for you.

A member of the team at your local hospital involved in this study will go through this information sheet with you and give you a copy to take away with you to discuss with friends and/or relatives, if you wish. They will answer any questions that you may have. If there is anything that is not clear within the information sheet, please ask your medical team.

**Take your time to decide whether or not you wish to take part. If you decide not to take part your doctors will continue to treat you in line with standard treatment and it will not affect the quality of your care.**

## Contents

**Part 1: What is this clinical study about?**

1. What is the purpose of this study?
2. What will happen to me if I take part?
3. What are the possible benefits and risks of taking part?
4. What happens at the end of the study?
5. Will anybody get paid if I take part?
6. What if there is a problem?
7. Will my taking part in this study be kept confidential?

**Part 2: What else do I need to know about taking part in this clinical study?**

1. More information about taking part
2. Who should I contact for further information



For peer review only - http://bmjopen.bmj.com/site/about/guidelines.xhtml

## Important things that you need to know

We want to improve the outcome for people like yourself who are living with a brain tumour. Specifically, in this study we are establishing a platform to deliver:

- Rapid and accurate detailed laboratory analysis of the molecular changes in brain tumours. This will enable accurate diagnosis in a timeframe useful to clinicians.
- To develop robust ways of collecting, processing and storing brain tumour tissue, radiology images and clinical data.
- An enhanced network of UK brain tumour clinical centres.
- The introduction of clinical trials of therapies tailored/targeted to the precise molecular and clinical features of individual patients.

## Who is Tessa Jowell?

This study is named after Baroness Tessa Jowell, the ex-politician and minister, who died from a glioma in summer 2018. As a result of her efforts to raise awareness of brain tumours, research funding is now being increased.

## Abbreviation and definitions

- Biofluids — Bodily samples that are liquid (e.g. blood, urine, saliva, tears, or cerebrospinal fluid
- DNA — Deoxyribonucleic acid
- GDPR — General Data Protection Regulation
- GEL — Genomics England
- Glioma — A type of brain tumour
- NHS — National Health Service
- REC — Research Ethics Committee
- TJBM — Tessa Jowell BRAIN MATRIX

We have sought to make this document readable and understandable by patients by involving patient representatives in writing this document. However, due to the nature of the study, and the legal and regulatory requirements to include certain information, we are aware that some of the wording can seem complex. This makes it especially important that you ask your research team about anything that you do not fully understand.

If you have any further questions, you are very welcome to contact the research team.


For peer review only - http://bmjopen.bmj.com/site/about/guidelines.xhtml

# Part 1:
# What is this clinical study about?

## 1. What is the purpose of this study?

### What is the purpose of this study?

The **Tessa Jowell BRAIN MATRIX** is a programme of work, the principal purpose of which is to improve the knowledge of, and treatment for, glioma, a type of brain tumour. The platform study forms the backbone for this.

Brain tumours are a challenging disease to treat. This is due to:

- The tumour's location within the brain, and its tendency to grow into nearby brain tissue, often make it very difficult or not possible to remove the tumour completely with surgery.
- The biology of brain tumours, including:
  - Their lack of response to traditional radiotherapy/chemotherapies.
  - Their ability to spread within the brain.
  - Their ability to change/adapt during/in response to treatments.
- Difficulties in delivering drugs in adequate amounts to the tumour, due to natural defences of the brain (the 'blood-brain barrier').
- A relative lack in research infrastructure for brain tumour patients within the NHS and academia in the UK.

Together this means that progress in treating brain tumours has not matched that of other cancers. In this study, we want to create an underlying treatment and analysis 'platform' to address these challenges of treating gliomas. This platform will bring together many different people including patients like you, brain surgeons, cancer doctors, technicians and scientists.

The standard treatment for most brain tumours is surgery. This is to remove the main bulk of the tumour and is followed by radiotherapy and/or chemotherapy. In the majority of cases, however, it is difficult for all of the tumour cells to be removed by the surgeon because of the way it invades, and the potential damage that can occur to the brain by removing too much tissue. Therefore, some tumour cells can be left behind which can regrow. Whilst radiotherapy and/or chemotherapy can delay this, their effects are often only temporary. There is, therefore, a need to develop and test new treatments.

Brain tumours arise due to changes in the DNA and other molecules in cells of the brain. Different types of gliomas can have different changes and these can be used to determine a precise 'molecular diagnosis'. The ultimate goal for the **Tessa Jowell BRAIN MATRIX** is to learn how to use these molecular changes to more precisely determine what exact type of tumour you have and to identify, decide and test whether specific 'targeted' treatments could improve the survival and/or quality of life of patients with brain tumours.

To achieve this, we need to develop the infrastructure to provide a rapid and accurate molecular diagnosis. A large network of clinical hubs across the UK, with expertise in managing patients with brain tumours, will be developed. This will enable:

- Collection of high-quality clinical data, including information about your quality of life, in a secure manner, maintaining patient confidentiality.
- Robust processes and safeguards for the collection, processing, analysis and storage of tumour tissue, including feedback to your treating doctors of a precise molecular diagnosis.
- Collection and expert review of images to assess responses of tumours to treatment in a standardised manner and in a time frame relevant to a patient's treatment.


For peer review only - http://bmjopen.bmj.com/site/about/guidelines.xhtml

Once established this infrastructure will facilitate the rapid introduction of clinical trials testing targeted therapies tailored to the genetic changes of an individual's tumour. In addition, collection of this data, images and samples, will provide an extremely valuable resource to help us to understand better the clinical behaviour, impact on patients, molecular pathology and imaging characteristics of brain tumours. This will enable clinicians and scientists to improve the quality of treatments available and the quality of lives of patients with brain tumours. This study will actively engage with partners within the NHS, universities, pharmaceutical industry and charitable sectors to translate the advances into benefits for patients.

### Why have I been invited to participate?

In most cases, you have been invited to take part in this study because your recent scan shows that there is a growth in your brain. It is likely that this is a glioma, a type of brain tumour, and that you will be undergoing surgery (or biopsy) to remove at least some of this tumour.

In some cases, you have been asked because you are known to have such a tumour, and are undergoing further surgery, or samples are available that can be used from your previous operations.

### Do I have to take part?

No, you do not have to take part. It is up to you to decide whether to join the study. A member of the local research team will describe the study and go through this information sheet with you. If you decide to take part, you will be asked to sign a consent form to show you have agreed to participate. You are free to withdraw at any time, without giving a reason. A decision not to take part or to withdraw later will not affect the standard of care you receive thereafter.

## 2. What will happen to me if I take part?

### What will happen if I decide to take part?

If you decide to take part in this study, you will be given this information sheet to keep, and you (and your study doctor) will be asked to sign a consent form to show that you have agreed to take part in this study. You will be given a copy of the signed consent form to take home with you. We will then need to confirm that you meet all the requirements and that you are fit enough to take part in the study. This is called screening. If you do not meet the inclusion criteria for this study you will not be able to participate. Decisions about your treatment are independent to your participation in the BRAIN MATRIX Platform Study and will continue to be made in discussion with you and your doctor.

For peer review only


For peer review only - http://bmjopen.bmj.com/site/about/guidelines.xhtml

**The diagram below shows a summary of the Tessa Jowell BRAIN MATRIX Programme:**

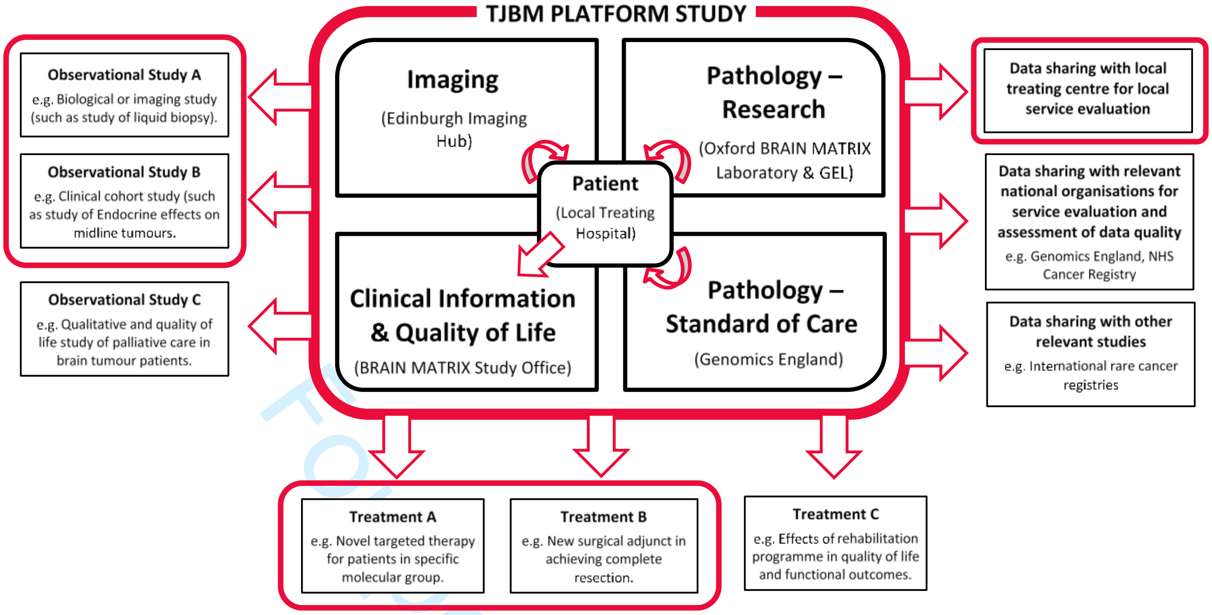


## Study Participation

If you are eligible for this study you will either have had, or be about to have, surgery for your tumour. As part of this study, tumour removed during your operation will be analysed to look for specific molecular changes. No additional surgery or extra tumour will be taken as a result of participation in this study. As with normal standard care, your tumour will be analysed by your local pathologist. A small part will be sent for review by experts and advanced molecular analysis will be undertaken (in most cases analyses called whole genome sequencing and epigenetic classification) to get a detailed understanding of the DNA/molecular changes within your tumour. These results will be fed back to the doctor treating you. It is intended that this will occur within 28 days; however, it may be longer while the study becomes fully operational. If samples are available from previous surgery to your tumours, we may also analyse these. Similarly, if available, other relevant samples, such as cerebrospinal fluid, collected as part of your care, may also be analysed. In addition, as technologies and analyses improve our understanding of brain tumours, we may find important results at a later date. These will be fed back to your doctor.

To perform these analyses you will be asked to give a blood sample, which will also be analysed to look at the molecular features, including of your DNA. This is required to identify what 'new' changes have occurred in your tumour.

Following your surgery you will continue with other treatment(s) as directed by your doctor. Treatment generally involves radiotherapy and chemotherapy and your doctor will discuss this with you. As is standard practice, you will be closely monitored for signs of disease progression and the effects of the treatment given.

As part of this study, information on your treatments and disease will be collected. This will include details about, your current health (e.g. as assessed by your doctor's assessment and examination, some blood test results), treatments you are taking/receiving (e.g. names of drugs, doses). Images from brain scans you undergo, along with relevant clinical information, will also be sent to and stored by the University of Edinburgh, and where appropriate, undergo expert review by a panel of

For peer review only


For peer review only - http://bmjopen.bmj.com/site/about/guidelines.xhtml

radiologists with expertise in brain tumours. You will not have any additional scans as part of this study. If you have further surgery, some of the tissue removed may also be analysed. As above, no additional surgery will be performed or extra-tumour will be removed as a result of participation in this study. You may also be asked to provide additional optional biofluid samples at the start or end of any specific treatments (e.g. at the start and end of radiotherapy).

We know that brain tumours can impact many areas of your life and we want to understand this better. To do this you will be asked to complete a 'Quality of Life' booklet that consists of four short questionnaires, initially when you join the study, then at key points in your treatment and when you are being monitored after treatment. These will generally be when you are attending the hospital to have your brain scans. A carer may assist you with completing the questionnaire, but the answers should be your own.

Patients joining BRAIN MATRIX will also be able to support a study, called BRIAN (the Brain tumouR Information and Analysis Network), run by the The Brain Tumour Charity.

BRIAN allows people to record their experiences of having a brain tumour using a mobile device or web app. This is to help doctors and researchers get a better understanding of how having a brain tumour affects the quality of life of patients who are living with a brain tumour or caring for someone who has a brain tumour. Your doctor will talk to you about participating in this additional study. In the future it is hoped that by analysing information collected by BRIAN together with all the other data collected from patients participating in BRAIN MATRIX, it will be possible to provide better overall care and support to patients and their families.

You can find out more about BRIAN and how to take part at the Brain Tumour Charity's website: https://www.thebraintumourcharity.org/living-with-a-brain-tumour/brian/.

You are welcome to take part in BRIAN even if you do not wish to take part in this study. Similarly, you can still participate in BRAIN MATRIX if you do not wish to join BRIAN.

## How long will I be in the study?

We would like to continue to collect data on you for up to five years where possible.

## What will I have to do?

If you decide to take part in this study, you will be required to:

- ✓ Sign a consent form to enter the study.
- ✓ Keep all scheduled medical and imaging appointments. (There are no additional appointments or scans as part of this study).
- ✓ At routine clinic visits your weight, neurological examination and performance status (measure of your general health and how your disease affects your daily routine) will be taken.
- ✓ Tell the study doctor about any medication that you are taking, even if it is medicine you buy without a prescription or is a natural or herbal remedy.
- ✓ Tell the doctor about any side effect, injury, symptom or complaint you experience, including any unplanned hospital admissions.
- ✓ Provide blood samples, and further optional biofluid samples, at the start and end of a treatment, and/or at tumour progression.
- ✓ Complete Quality of Life questionnaires when you join the study and when requested (usually at the start of a treatment, and/or at tumour progression, and otherwise every 3-6 months).



For peer review only - http://bmjopen.bmj.com/site/about/guidelines.xhtml

### What are the alternatives for treatment?

If you choose not to take part in this study you will receive the same treatments as if you were on the study.

## 3. What are the possible benefits and risks of taking part?

### What are the possible benefits of taking part?

We cannot promise that you will benefit directly from participating in this study. All the information that we get from this study will help improve the treatment of patients with glioma in the future.

By participating in this study, it will allow us to identify certain molecular characteristics of your tumour. These analyses can help refine and, in some cases, change the diagnosis. Your doctor will then be able to adapt and discuss the best treatment options for you.

This study may identify molecular changes in your tumour that suggest you could benefit from new-targeted treatments, which could be made available to you in the future or make you eligible for another clinical study/trial. Your doctor will discuss your options with you.

**Please note that identification of a target does not guarantee benefit from, or access to, a targeted treatment.**

### What are the possible disadvantages and risks of taking part?

We want to try and improve the outcome for patients with glioma and believe that providing this standardised platform may improve outcomes in, and options for, patients. However, it is possible that this may not show any benefit over the current UK standard practice.

### What about genetic results?

In addition to identifying changes in your tumour, it is possible that the genetic analysis might discover an unexpected genetic change in your normal cells. Genetic changes in normal cells may tell your doctors that you could be at risk of another disease. This might be a cancer or another disease that has nothing to do with your cancer. Changes like this could be present in other members of your family or passed on to your children. It is important to emphasise that the aim of this study is not to identify genes that may be inherited and cause cancer in families.

In the unlikely event that studies on your blood or tumour reveal genetic information that may affect you or other family members, we need to know whether you would wish to be informed. If you wish to be informed about these genetic changes, please initial the corresponding box on the informed consent form. We will then tell the doctor treating you at your local centre so that they can discuss the finding with you and arrange for any genetic counselling you may need. Please note that these unexpected genetic findings will not affect how your brain tumour is treated and will not prevent you from participating in this study.

You may choose to opt in or opt out of receiving information about genetic changes in normal cells on the informed consent form.

### What are the side effects of the treatment?

There are no specific treatments within this study. Your doctor will discuss the side effects of your recommended treatment.



For peer review only - http://bmjopen.bmj.com/site/about/guidelines.xhtml

## 4. What happens at the end of the study?

The study team will continue to collect data about your health and further treatment for up to 5 years where possible. This data will be collected at routine clinic appointments.

## 5. Will anybody get paid if I take part?

You will not receive any money or study expense reimbursement for taking part in this clinical study.

## 6. What if there is a problem?

If you have any concerns about your care during this study or any possible harm you may suffer, you should inform your study doctor immediately. More detailed information is given in Part 2.

## 7. Will my taking part in this study be kept confidential?

Yes. We will follow ethical and legal practice and all information about your participation in this study will be kept confidential. The details of this are in Part 2 of this information sheet.

For peer review only

**This completes Part 1 of the Information Sheet**

If the information in Part 1 has interested you and you are considering participation, please read the additional information in Part 2 before making any decision.


For peer review only - http://bmjopen.bmj.com/site/about/guidelines.xhtml

# Part 2:
# What else do I need to know about taking part in this clinical study?

## 8. More information about taking part

### What if relevant new information becomes available?

Sometimes new information about the conditions being studied becomes available during the course of a research study. If this happens, your study doctor will tell you about it and discuss this with you. Your treatment is not affected by inclusion in this study, and therefore it is unlikely to influence whether you should continue in the study. If you decide not to carry on, your study doctor will make arrangements for your care to continue as normal. If you decide to continue with the study then you may be asked to sign an updated consent form. If the study is stopped for any other reason, your doctor will tell you and arrange your continuing care.

### What if I decide that I don't want to carry on with the study?

You are free to withdraw from this study at any time. You do not have to give a reason for your decision and your future treatment will not be affected. Any data already collected prior to withdrawal may still be used for study purposes.

### What if there is a problem?

In the event that something does go wrong and you are harmed because of taking part in the trial, and this is due to someone's negilence, then you may have grounds for claiming compensation from the Sponsor of the trial (the University of Birmingham) or the NHS Trust who treated you but you may have to pay your legal costs.

NHS Trust and non-Trust Hospitals have a duty of care to patients treated, whether or not the patient is taking part in a clinical trial, and the normal NHS complaints mechanism will still be available to you (if appropriate).

### What will happen to any samples I give?

Collection of diagnostic biopsy samples, taken as part of your standard care, and blood samples at study entry are mandatory and are required for the genomic analysis to be performed. In addition, we will ask that pathology reports on your tissue samples are sent to the BRAIN MATRIX Study Office and to University of Oxford. Any samples remaining at the end of this analysis will be stored in a biobank and may be used in future ethically approved research, which may involve genetic analysis, animal or *in vitro* models, commercial or private institutions, and which may take place in the UK or overseas. If you change your mind about the use of your samples in the study, we will not collect any more blood samples and, if you request it, we will destroy all that remains of samples already taken.

### Will my taking part in this study be kept confidential?

Yes. All information collected about you for this study will be subject to the General Data Protection Regulation (GDPR) and the Data Protection Act 2018 and will be kept strictly confidential. University of Birmingham is the Sponsor for this study based in the UK. We will be using information from your medical records in order to undertake this study and will act as the data controller for this study. This means that we are responsible for looking after your information and using it properly. University of Birmingham and the NHS will keep identifiable information about you for at least 25 years after the study has finished, allowing the results of the study to be verified if needed.


For peer review only - http://bmjopen.bmj.com/site/about/guidelines.xhtml

All information collected by the Sponsor will be securely stored at CRCTU at the University of Birmingham (BRAIN MATRIX Study Office) on paper and electronically and will only be accessible by authorised personnel. The only people at the University of Birmingham who will have access to information that identifies you will be people who manage the study or audit the data collection process. With your permission, your study doctor will provide your initials, date of birth, NHS/CHI number and hospital number when they enter you into the study and they will notify your GP that you intend to participate. They will also send a copy of your signed Informed Consent Form in the post to the BRAIN MATRIX Study Office.

The NHS will use your name and contact details to contact you about the research study, and make sure that relevant information about the study is recorded for your care, and to oversee the quality of the study. Your study doctor or research nurse may also need to send a copy of your Informed Consent Form to other healthcare professionals (e.g. your GP or NHS pathologist) to prove that you have given consent to take part in the study before they will provide information or tumour samples.

In the BRAIN MATRIX Study Office you will be identified by a unique study number. In routine communication between your hospital and the BRAIN MATRIX Study Office you will only be identified by your study number, initials and date of birth. We may need to record, and occasionally refer to you using your hospital, Genomics England (GEL) Referral ID number and histopathology numbers during analysis of your tumour samples and radiology numbers during analysis of your MRI scans. In addition, your study number, hospital, NHS, histopathology and radiology numbers may be included on samples sent to University of Oxford, University of Edinburgh and Genomics England for central review to help specialist pathologists and radiologists identify the tumour samples and MRI data.

All information will be treated as strictly confidential and nothing that might identify you will be revealed to any third party other than those involved in the treatment or organisation of tumour sample and imaging collection and transfer (e.g. staff at University of Birmingham, University of Oxford, University of Edinburgh and Genomics England). It may be necessary to send information about you such as study number and date of birth to the BRIAN databank (IRAS ID: 237931) run by The Brain Tumour Charity. This is for your, and others', protection to track the safety of the treatments used. We will also provide anonymised linked data to QMENTA, a neuroimaging storage platform, for research purposes. All third parties have the same duty of confidentiality to you as all other research study personnel.

By taking part in the study you will be agreeing to allow research staff at your hospital, and from the BRAIN MATRIX Study Office, to look at the study records, and this includes your medical records. It may be necessary to allow authorised personnel from the University of Birmingham and/or NHS bodies to have access to your medical and research records. This is to ensure that the study is being conducted to the highest possible standards. We may also collect different aspects of your health data from the NHS and other Department of Health organisations, in addition to your medical records, for the purposes of long term followup.

From time to time we may be asked to share the study information (data) we have collected with researchers running other studies in this organisation and in other organisations, so that they can perform analysis on the data to answer other important questions about brain tumours. This may also reveal clinical trials or other research that you might benefit from, which your doctor or another member of your clinical team may discuss with you if appropriate. These organisations may be universities, NHS organisations or companies involved in health research and may be in this country or


For peer review only - http://bmjopen.bmj.com/site/about/guidelines.xhtml

abroad. Any such request is carefully considered by the study researchers and will only be granted if the necessary procedures and approvals are in place. This information will not identify you and will not be combined with other information in a way that could identify you. The information will only be used for the purpose of health research, and cannot be used to contact you or to affect your care. It will not be used to make decisions about future services available to you, such as insurance. Under no circumstances will you be identified in any way in any report, presentation or publication arising from this or any other study.

All individuals who have access to your information have a duty of confidentiality to you.

If you choose to withdraw from the study treatment, we would still like to collect relevant information about your health, as this will be invaluable to our research. If you have any objection to this, please let your study doctor know.

You can withdraw your consent to our processing of your data at any time. Your rights to access, change, or move your information are limited, as we need to manage your information in specific ways in order for the research to be reliable and accurate. If you withdraw from the study, we will keep the information about you that we have already obtained. To safeguard your rights, we will use the minimum personally-identifiable information possible. Under the provisions of the General Data Protection Regulation you have the right to know what information the BRAIN MATRIX Study Office have recorded about you. If you wish to view this information or find more about how we use this information, please contact Legal Services at the address below.

Legal Services
University of Birmingham
Edgbaston
BIRMINGHAM
B15 2TT

More information about how your data will be handled can be found in the CRCTU Privacy Policy available on our website: www.birmingham.ac.uk/crctu.

## Where can you find out more about how your information is used?

You can find out more about how we use your information in the GDPR Guidance document provided to you with this information sheet.

## What will happen to the results of the study?

Results from the study will be published in medical journals but no individual patients will be identified. You will be able to get a copy of the published results by asking your doctor at the end of the study.

## Who is organising and funding the research?

This research study is being carried out by a network of doctors across the UK. The study is sponsored by the University of Birmingham and co-ordinated by the Cancer Research UK Clinical Trials Unit at the University of Birmingham. The study is being funded by The Brain Tumour Charity, and some aspects of the genomic testing will be supported by Genomics England, part of Department of Health & Social Care. Your study doctor will not be paid extra for including you in this study.


For peer review only - http://bmjopen.bmj.com/site/about/guidelines.xhtml

### Who has reviewed the study?

All research in the NHS is looked at by an independent group of people, called a Research Ethics Committee (REC) to protect your safety, rights, well-being and dignity. This study has been reviewed and given favourable ethical opinion by the West Midlands – Edgbaston Research Ethics Committee. It has also been approved by the local Trust Research and Development department at your hospital.

### Involvement of the General Practitioner/Family Doctor (GP)

It is important that your GP is informed that you are taking part in this study. In addition, we may ask him/her to provide information on your progress. If we need to contact your GP for any follow-up information, we will need to use your full name in our correspondence.

## 9. Who should I contact for further information?

### Further information and contact details

If you have any questions or concerns about your disease or this clinical study, please discuss them with your doctor.

For more generic information about treating cancer, you may also find it helpful to contact '**About Cancer**', an information service about cancer and cancer research studies by Cancer Research UK:

**Freephone:** 0808 800 40 40

**Website:** www.cancerresearchuk.org/about-cancer/brain-tumours

For more information and support about brain tumours, you may find it helpful to contact **The Brain Tumour Charity**:

**Freephone:** 0808 800 0004

**Email:** support@thebraintumourcharity.org

**Website:** www.thebraintumourcharity.org/

### Your telephone contact numbers:

**Local Investigator:** ............................................ **Contact Number:** ............................................

**Research Nurse:** ............................................ **Contact Number:** ............................................

**Emergency (24 hours) Contact Number:** ........................................

**Thank you for taking time to read this Patient Information Sheet and considering taking part in this study.**



For peer review only - http://bmjopen.bmj.com/site/about/guidelines.xhtml

# Summary of Assessments

| Assessment / Activity | Screening | Platform Entry | Day 1 -28 | Start of concomitant/ adjuvant treatment | Further Surgery *(if applicable)* | Follow-up – up to 5 years |
|---|---|---|---|---|---|---|
| Informed Consent | ✓ | | | | | |
| Confirm eligibility | ✓ | | | | | |
| Registration | | ✓ | | | | |
| Baseline Clinical Data Collection | | ✓ | | | | |
| Diagnostic Surgery | | ✓ | | | ✓ | |
| Collection of blood sample (mandatory) | | ✓ | | | | |
| Collection of blood samples (optional) | | ✓ | | ✓ | ✓ | ✓ |
| Neurological Examination | | ✓ | | ✓ | ✓ | ✓ |
| Performance Status | | ✓ | | ✓ | ✓ | ✓ |
| Weight | | ✓ | | ✓ | ✓ | ✓ |
| Quality of Life Questionnaires | | ✓ | | ✓ | ✓ | ✓ |
| Blood and biopsy samples sent to Oxford | | ✓ | | | ✓ | |
| Imaging data sent to Edinburgh | | ✓ | | ✓ | ✓ | ✓ |
| Local pathology report sent to BRAIN MATRIX Study Office and Oxford | | | ✓ | | ✓ | |
| Molecular testing and feedback | | | ✓ | | ✓ | |
| Reporting of relapse, further treatment | | | ✓ | ✓ | ✓ | ✓ |

For peer review only



For peer review only - http://bmjopen.bmj.com/site/about/guidelines.xhtml

## Supplementary Appendix 6: The Tessa Jowell BRAIN MATRIX Platform study schedule of events

| Activity* | Screening | Platform Entry | Day 1-28 | Initiation of concomitant therapy | Initiation of adjuvant therapy | Further surgery (if applicable) | Follow-up – up to 5 years[#] | Death |
|---|---|---|---|---|---|---|---|---|
| Informed consent[1] | X | | | | | | | |
| Confirm eligibility | X | | | | | | | |
| Registration | | X | | | | | | |
| Baseline Clinical Data Collection[2] | | X | | | | | | |
| Collection of blood for germline DNA | | X | | | | | | |
| Collection of Liquid biopsies – blood[3] | | X | | X | X | X | X | |
| Diagnostic surgery (biopsy or craniotomy) | | X | | | | X | | |
| Shipment of matched tumour and blood sample to Oxford BRAIN MATRIX Laboratory for molecular diagnosis[4] | | X | | | | X | | |
| Transfer of pseudo-anonymised imaging data to Edinburgh Imaging Hub[5] | | X | | X | X | X | X | |
| Clinician Global Impression of Change[6] | | | | X | X | X | X | |
| NIH Stroke Score[7] | | X | | X | X | X | X | |
| Weight[8] | | X | | X | X | X | X | |
| WHO Performance Status[9] | | X | | X | X | X | X | |
| Quality of Life questionnaires[10] | | X | | X | X | X | X | |
| Local pathology report sent to BRAIN MATRIX Study Office[11] | | | X | | | X | | |



For peer review only - http://bmjopen.bmj.com/site/about/guidelines.xhtml

| Activity* | Screening | Platform Entry | Day 1-28 | Initiation of concomitant therapy | Initiation of adjuvant therapy | Further surgery (if applicable) | Follow-up – up to 5 years# | Death |
|---|---|---|---|---|---|---|---|---|
| Reporting of relapse, further treatment and death | | | X | X | X | X | X | X |
| Post-mortem report and submit tissue specimens to CRUK PEACE study[12] | | | | | | | | X |

**Notes**

* Where applicable and acceptable in accordance to local practices, visits/assessments may be performed by telephone or video call.

# Follow-up visits to occur every 6 months and at clinic visit that coincides with the time point.

1. Written informed consent must be obtained within 28 days of Platform Entry and before any study-specific screening procedures.
2. Baseline Clinical Data Collection to include height.
3. Further optional blood samples should be collected whenever possible for future analysis of cell-free circulating tumour DNA (cfT-DNA) at the following time points: At first post-operative MRI; Initiation of adjuvant treatment (if applicable); the end of adjuvant treatment (if applicable); the time of objectively measured progression; during the palliative phase if a post-mortem has been agreed. Cerebrospinal fluid, if available for the patient, should also be submitted.
4. Local sites will register their samples (blood and frozen tissue) after the patient has been registered to the study and send them to the Oxford BRAIN MATRIX Laboratory for biobanking, biofluid analysis and complementary omics. For those cases that are recruited through the NHS Genomic Medicine Service (GMS) WGS pathway for Whole Genome Sequencing (WGS) in England;, a (small) tumour sample and one EDTA blood sample will be sent directly to the respective Genomics Laboratory Hub (GLH), once standard of care paired tumour blood WGS analysis is established. Patients must be consented to both the NHS GMS WGS pathway and BRAIN MATRIX. A copy of the Sample Form must also be sent to the BRAIN MATRIX Study Office. Please refer to the BRAIN MATRIX Platform Laboratory Manual for further details. Clinical data to also be included on the Sample Form.
5. Refer to the Imaging Manual for further details. Clinical data to also be included on Imaging Form. Neurosurgical navigation and radiotherapy planning imaging to be submitted in addition to MRI.
6. **Error! Reference source not found.**To be collected at the start and end of concomitant therapy and start and end of adjuvant treatment. For Further Surgery time point, to be collected at the patient's post-operative review. The Clinician Global Impression of Change score should also be collected around the time of imaging and submitted with the imaging to facilitate RANO assessment. The Clinician Global Impression of Change can be completed by Investigators or Research Nurses.
7. **Error! Reference source not found.**To be collected at the start and end of concomitant therapy and start and end of adjuvant treatment. For Further Surgery time point, to be collected at the patient's post-operative review. The NIH Stroke Score can be completed by Investigators or suitably trained Research Nurses.



For peer review only - http://bmjopen.bmj.com/site/about/guidelines.xhtml

8. Weight to be collected at the start and end of concomitant therapy and start and end of adjuvant treatment. For Further Surgery time point, to be collected at the patient's post-operative review.
9. **Error! Reference source not found.**To be collected at the start and end of concomitant therapy and start and end of adjuvant treatment. For Further Surgery time point, to be collected at the patient's post-operative review.
10. QoL Questionnaires to be completed at follow-up visits that coincide with imaging appointments. **Error! Reference source not found.**
11. Pseudonymised copy of local pathology report for each sample must be sent to the BRAIN MATRIX Study Office as soon as it is available. Once received, it will be shared with the Oxford BRAIN MATRIX Laboratory for Genomic Tumour Advisory Board discussion. Submission of this report is the responsibility of the recruiting site.
12. Separate consent to the CRUK PEACE study (or equivalent) to be obtained.

For peer review only



For peer review only - http://bmjopen.bmj.com/site/about/guidelines.xhtml

# Supplementary Appendix 7: Definition of adverse events

## Adverse Event (AE)

Any untoward medical occurrence in a patient or clinical trial subject participating in the study which does not necessarily have a causal relationship with the treatment received.

Comment:

An AE can therefore be any unfavourable and unintended sign (including abnormal laboratory findings), symptom or disease temporally associated with the use of a medicinal product, whether or not related to the medicinal product.

## Related Event

An event which resulted from the administration of any of the research procedures.

## Serious Adverse Event (SAE)

An untoward occurrence that:

- Results in death
- Is life-threatening*
- Requires hospitalisation** or prolongation of existing hospitalisation
- Results in persistent or significant disability or incapacity
- Consists of a congenital anomaly/ birth defect
- Or is otherwise considered medically significant by the Investigator***

Comments:

The term severe is often used to describe the intensity (severity) of a specific event. This is not the same as serious, which is based on patients/event outcome or action criteria.

* Life threatening in the definition of an SAE refers to an event in which the patient was at risk of death at the time of the event; it does not refer to an event that hypothetically might have caused death if it were more severe.

** Hospitalisation is defined as an unplanned, formal inpatient admission, even if the hospitalisation is a precautionary measure for continued observation. Thus, hospitalisation for protocol treatment (e.g., line insertion), elective procedures (unless brought forward because of worsening symptoms) or for social reasons (e.g., respite care) are not regarded as an SAE.

For peer review only


For peer review only - http://bmjopen.bmj.com/site/about/guidelines.xhtml

*** Medical judgment should be exercised in deciding whether an AE is serious in other situations. Important AEs that are not immediately life threatening or do not result in death or hospitalisation but may jeopardise the subject or may require intervention to prevent one of the other outcomes listed in the definition above, should be considered serious.

### Unexpected and Related Event

An event which meets the definition of both an Unexpected Event and a Related Event.

### Unexpected Event

The type of event that is not listed in the protocol as an expected occurrence.

For peer review only


For peer review only - http://bmjopen.bmj.com/site/about/guidelines.xhtml

BMJ Open

# Protocol for the Tessa Jowell BRAIN MATRIX Platform Study

| | |
|---|---|
| Journal: | *BMJ Open* |
| Manuscript ID | bmjopen-2022-067123.R1 |
| Article Type: | Protocol |
| Date Submitted by the Author: | 17-Aug-2022 |
| Complete List of Authors: | Watts, Colin; University of Birmingham College of Medical and Dental Sciences; University Hospitals Birmingham NHS Foundation Trust<br>Savage, Joshua; University of Birmingham College of Medical and Dental Sciences<br>Patel, Amit; University of Birmingham College of Medical and Dental Sciences<br>Mant, Rhys; University of Birmingham College of Medical and Dental Sciences<br>Wykes, Victoria; University of Birmingham College of Medical and Dental Sciences; University Hospitals Birmingham NHS Foundation Trust<br>Pohl, Ute; University Hospitals Birmingham NHS Foundation Trust<br>Bulbeck, Helen; Brainstrust<br>Apps, John; University of Birmingham College of Medical and Dental Sciences<br>Sharpe, Rowena; University of Birmingham College of Medical and Dental Sciences<br>Thompson, Gerard; University of Edinburgh Brain Research Imaging Centre<br>Waldman, Adam; University of Edinburgh Brain Research Imaging Centre<br>Ansorge, Olaf; Oxford University<br>Billingham, Lucinda; University of Birmingham College of Medical and Dental Sciences |
| <b>Primary Subject Heading</b>: | Neurology |
| Secondary Subject Heading: | Surgery |
| Keywords: | NEUROSURGERY, Neurological oncology < ONCOLOGY, Clinical trials < THERAPEUTICS |
| | |

SCHOLARONE™
Manuscripts

# Protocol for the Tessa Jowell BRAIN MATRIX Platform Study

Colin Watts[1†,2], Joshua Savage[3], Amit Patel[3], Rhys Mant[3], Victoria Wykes[1,2], Ute Pohl[2], Helen Bulbeck[4], John Apps[3], Rowena Sharpe[3], Gerard Thompson[5], Adam Waldman[5], Olaf Ansorge[6], Lucinda Billingham[3] and the TJBM Investigators*

[†] Corresponding author contact details:

Professor Colin Watts. Institute of Cancer and Genomic Sciences, University of Birmingham, Edgbaston, Birmingham. UK. Email: C.Watts.2@bham.ac.uk

*Additional Investigators of the BRAIN MATRIX study are listed in Supplementary Appendix 1.

[1] Institute of Cancer and Genomic Sciences, University of Birmingham, Edgbaston, Birmingham. UK

[2] University Hospitals Birmingham NHS Foundation Trust, Birmingham. UK

[3] Cancer Research UK Clinical Trials Unit (CRCTU), University of Birmingham, Edgbaston, Birmingham. UK

[4] brainstrust, Cowes, Isle of Wight. UK

[5] Centre for Clinical Brain Sciences, University of Edinburgh, Edinburgh. UK

[6] Nuffield Department of Clinical Neurosciences, University of Oxford, Oxford. UK

For peer review only

# Abstract

## Introduction

Gliomas are the most common primary tumour of the central nervous system (CNS), with an estimated annual incidence of 6.6 per 100,000 individuals in the USA and around 14 deaths per day from brain tumours in the UK. The genomic and biological landscape of brain tumours has been increasingly defined and, since 2016, the WHO classification of tumours of the CNS incorporates molecular data, along with morphology, to define tumour subtypes more accurately. The Tessa Jowell BRAIN MATRIX Platform (TJBM) study aims to create a transformative clinical research infrastructure that leverages UK NHS resources to support research that is patient centric and attractive to both academic and commercial investors.

## Methods and analysis

The TJBM study is a programme of work with the principal purpose to improve the knowledge of glioma and treatment for glioma patients. The programme includes a platform study and subsequent interventional clinical trials (as separate protocols). The platform study described here is the backbone data-repository of disease, treatment and outcome data from clinical, imaging and pathology data being collected in glioma patients from secondary care hospitals. The primary outcome measure of the platform is time from biopsy to integrated histological-molecular diagnosis using whole genome sequencing and epigenomic classification. Secondary outcome measures include those that are process-centred, patient-centred and framework-based. Target recruitment for the study is 1000 patients with interim analyses at 100 and 500 patients.

## Ethics and dissemination

The protocol was approved by West Midlands - Edgbaston Research Ethics Committee. Participants will be required to provide written informed consent. The results of this study will be disseminated through national and international presentations and peer-reviewed publications.

## Trial registration number

ClinicalTrials.gov Identifier: NCT04274283; 18-Feb-2020

ISRCTN 14218060; 03-Feb-2020


# Strengths and limitations of this study

- The TJBM study is a programme of work, which will improve the knowledge of glioma, and treatment for glioma patients.
- This platform study is a backbone data-repository of disease, treatment and outcome data from clinical, imaging and pathology data
- The TJBM study will aim to provide rapid and accurate molecular diagnosis, a network of clinical hubs with robust protocols for the collection, processing, analysis and storage of tissue, images, and clinical and quality of life data
- Gliomas occur at all ages and their specific subtype is difficult to predict preoperatively, therefore, the patient population eligible for the study is broad but currently excludes <16-year-old patients

For peer review only

## Introduction

Gliomas are the most common primary tumour of the central nervous system (CNS), with an estimated annual incidence of 6.6 per 100,000 individuals in the USA (1), which is predicted to rise to 22/100,000 by 2035 (2). In 2016, there were 5,250 deaths from brain tumours in the UK i.e., 14 deaths per day (2). Malignant CNS tumours hold the poorest prognosis, and are responsible for the highest estimated number of years of potential life lost (mean 20 years) amongst all cancers (3) and survival trends have remained generally static in comparison to other cancers (4).

Gliomas have traditionally been divided into low grade glioma (LGG; WHO I-II) and high-grade glioma (HGG; WHO III-IV) based on integrated classic histological features and molecular biomarkers (5). LGG have an indolent course with patients commonly surviving a decade after diagnosis (6). However, the natural history of many WHO Grade II LGG is progression to HGG. Approximately half of all newly diagnosed gliomas are classified as glioblastoma (GB; WHO IV), the most malignant type of brain cancer. Currently, the GB annual incidence is 3.2 per 100,000 population in the USA. This tumour occurs more frequently with advancing age; ranging from 0.4 per 100,000 population aged 20–34 years, to over 15 per 100,000 population aged 75-84 years (1). It is widely recognized that elderly populations are rapidly increasing globally and this will have a significant impact on the burden of GB disease. Despite this, most studies still focus on patients younger than 65 years.

The current gold standard treatment for newly diagnosed GB is surgical gross total resection (GTR), followed by radiotherapy with concomitant and adjuvant temozolomide. The aim of treatment is to delay tumour progression and extend overall survival (7). Despite decades of refinement, this approach results in a median survival time of only 12-14 months.

Over the last 20 years the genomic and biological landscape of brain tumours has been increasingly defined, refining previous classification systems, unravelling intra- and inter-tumoural heterogeneity and progression, identifying drug targets and potential therapeutic strategies, and better characterising challenges to therapies, such as the blood brain barrier (BBB) and mutational escape. In 2016, the WHO published its classification of tumours of the CNS (5), which for the first time used molecular data, along with morphology, to define tumour subtypes more accurately. This stratification integrated a combination of specific genetic and epigenetic biological characteristics that are changing rapidly as our knowledge evolves (8). In recognition of this rapid evolution of knowledge, 'cIMPACT-NOW' (Consortium to Inform Molecular and Practical Approaches to CNS Tumor Taxonomy – Not Official WHO) (9) was created, with the new WHO 2021 classification representing a consensus opinion based on new insights into the molecular definition of brain tumours from this collaboration (10). As analytical technologies evolve and become more affordable, genome-wide analyses combined with other so-called 'omics' data will lead to further refinement of tumour classification with meaningful impacts on prognosis and choice of therapy. This is the motivation for the TJBM study, which will adopt whole-genome sequencing (WGS) and epigenomic analysis of tumour samples.

The TJBM study aims to create a transformative clinical research infrastructure that leverages NHS resources to support research that is patient centric and attractive to both academic and commercial investors. Our approach will be holistic and flexible so that it can rapidly adapt to scientific advances and rigorously evaluate new drugs and technologies. To achieve this, it will establish a research-active network of clinicians and scientists who are well supported by an innovative trial infrastructure to deliver the following:

For peer review only

- Rapid and accurate molecular diagnosis ensuring precise classification of tumours and identifying subsets of patients suitable for targeted therapy, by building on the legacy of the 100,000 Genomes Project (11).
- A network of clinical hubs resourced to maximise patient recruitment and collect tissue and data.
- Robust protocols for the collection, processing, analysis and storage of tissue, images and clinical and quality of life (QoL) data, building on existing infrastructure such as UK Biobank (12), Medical Research Council (MRC) Brain Banks Network (13), and the CRUK PEACE study (14).
- High-quality biological samples that are fully clinically and radiologically annotated facilitating further biological and radiological research.
- Links to national and international clinical and scientific infrastructure and networks (15); e.g., Genomics England (GEL), European Network for Rare adult solid Cancer (EURACAN) (16), SIOP-E Brain Tumour Group, and National Cancer Research Institute (NCRI) Groups.
- Access to novel and repurposed drugs and technologies through collaborative partnerships with industry and early phase trials hubs and the structural genomics consortium (www.thesgc.org), to develop novel trials, within and outside, the TJBM infrastructure for testing of therapeutic strategies, including novel agents. As such therapeutic clinical trials will be developed using this infrastructure to maximise efficiency in introducing and rigorously evaluating novel interventions. These will be separate protocols to the TJBM study.
- Long-term sustainability through delivery of clinically and scientifically meaningful outcomes, leveraged investigator-led research grants and an established cost-recovery model for biobanking (17).

# Methods and analysis

## Study design

The TJBM study is a programme of work the principal purpose of which is to improve the knowledge of glioma and treatment for glioma patients. The programme will include a platform study and subsequent interventional clinical trials (as separate protocols); Figure 1. This platform study, is the backbone data-repository of disease, treatment and outcome data from clinical, imaging and pathology data to be collected in glioma patients from secondary care hospitals. Figure 2 shows an overview of the platform's study schema.

The study aims to recruit 1000 patients within the UK over 5 years with participants followed up for up to 5 years. An initial 10 UK centres were opened to the TJBM study, as listed in Supplementary Appendix 1, although further centre expansion is planned. The Standard Protocol Items: Recommendations for Intervention Trials (SPIRIT) checklist is provided as Supplementary Appendix 2 (18). The World Health Organization (WHO) Trial Registration Data Set is provided in Supplementary Appendix 3.

## Patient and public involvement

Patient and public involvement and engagement (PPIE) has been integral to this study from its inception. Our patient and public advisors, Helen Bulbeck and Peter Buckle, co-developed the TJBM study by reviewing and refining the protocol and the participant-facing documents. They have provided input into the patient-reported outcome measures and have guided messaging about the study for the community. As members of the Study Management Group (SMG; Supplementary Appendix 1) they continue to assess study conduct, and will contribute to the interpretation and dissemination of study findings through the

For peer review only - http://bmjopen.bmj.com/site/about/guidelines.xhtml

PPIE dissemination strategy. This will include presentation of the study findings to identified audiences, the best channel for reach, when to communicate, messaging and sensitivities.

## Patient selection

Patients aged ≥16 years with newly diagnosed suspected WHO Grade II-IV glioma, (as evidenced radiologically) suitable for a diagnostic or therapeutic surgical procedure resulting in a tumour sample matched to a blood sample or with progression with known WHO Grade II-IV glioma will be eligible.

Patients with primary spinal cord tumours, who are receiving active treatment of other malignancy, have contraindications to magnetic resonance imaging (MRI) and/or without standard of care imaging available, will be excluded.

Newly diagnosed patients with suspected WHO Grade II-IV glioma who are subsequently found to have a WHO Grade I tumour or non-brain tumour are expected to be a rare occurrence. If this event occurs:

- If it is confirmed as a Grade I tumour, then the patient will remain eligible for the study and will continue to be followed up in accordance with the protocol.
- If it is confirmed as a non-brain tumour, then the patient will not remain on the study and no further follow-up data will be collected.

All patients must be able to provide written informed consent for the study.

## Consent

Supplementary Appendix 4 contains exemplar informed consent forms (ICF), with Supplementary Appendix 5 the patient information sheets (PIS) and lay summary for the study. The investigator or an appropriately trained delegate must obtain written informed consent for each patient prior to performing any study related procedure. Remote Consent for platform entry is permitted by telephone or video consultation instead of face-to-face consultations.

In addition, to consenting to join TJBM, patients will be required to provide agreement for:

- The collection and analysis of biological samples (e.g., tumour, blood), including access to existing and future samples.
- The collection of relevant clinical information, including imaging and pathology.
- The return of clinically relevant results back to the referring clinician.
- The use of, and sharing of data, for research, teaching, commercial and scientific purposes, including data sharing through The Brain Tumour Charity's BRIAN (the Brain tumouR Information and Analysis Network) database (19).
- The collection of different aspects of health data from the NHS and other Department of Health organisations (in addition to medical records) for longitudinal analysis and follow-up.
- The use of clinical data to identify potential clinical trials or other research that they may benefit from.
- The sharing of samples for other ethically approved research projects.

In addition, and in line with GEL processes and studies such as the 100,000 Genomes Project, optional consent will be taken as to whether the patient would like to receive feedback about the evidence of inherited diseases (both underlying the cause of the cancer and non-cancer causes).

For peer review only - http://bmjopen.bmj.com/site/about/guidelines.xhtml

For centres submitting tumour tissue for WGS through the standard of care (SoC) NHS Genomic Medicine Service (GMS) pathway in England, an additional GEL consent step is currently required to confirm consent at the point of referral for the patient's clinical indication. This additional GEL consent step is performed electronically or on paper and can be completed over the phone.

## Platform assessments and schedule of events

Platform entry requires baseline clinical data, NIH Stroke Scale (20), weight, and WHO Performance Status to be recorded, and then performed again at start and end of concomitant therapy, start and end of adjuvant therapy, further surgery and during the five-year follow-up period.

In addition, QoL questionnaires will be completed at the initiation of concomitant therapy, initiation of adjuvant therapy, further surgery and during follow-up. The QoL booklet includes EQ-5D-5L (21), EORTC-QLQ-c30 (22), Patient Concerns Inventory (PCI) (23), Patient Global Impression of Change (PGIC) (24), and Clinician Global Impression of Change (25).

The schedule of events in included in Supplementary Appendix 6.

## Platform study outcome measures

The primary outcome measure of the platform is time from biopsy to integrated histological–molecular diagnosis (TTMD) as defined as the difference (in days) between date of biopsy and date of WGS and epigenomic classification.

Secondary outcome measures include those that are process-centred, patient-centred, and framework-based.

Process-centred secondaries include: time to completion of each node of tissue and imaging pathway; tumour and biological sample(s) quality control (QC) status; imaging QC status, and; inter-rater agreement of response assessment in neuro-oncology (RANO) (26).

Patient-centred secondaries include: extent of surgical resection; overall survival time (OS); intracranial progression-free survival time (PFS); QoL scores; type of interventions received; type of complications from treatments (standard of care) received, and; concordance between initial local radiological diagnosis, local pathological diagnosis, and integrated histological-molecular diagnosis.

Research framework-based secondaries include: samples and images centrally stored; targetable mutation(s) identified; post-mortem sampling consent status and sample collection confirmation, and; number of applications to, and outputs resulting from data repository (including trial proposals both within and outside of the TJBM network).

## Statistical analysis plan

The target sample size for the study is 1000 patients. The primary remit of TJBM study is to establish a central data repository that will support the development and delivery of precision medicine for all glioma patients in the UK. As such, there is no statistical basis behind the choice of target sample size, but this number will allow robust assessment of feasibility and subgroup analyses. Sample size for any clinical trials that are subsequently linked to the platform will be based on statistical justification.

A formal interim analysis of the primary outcome measure (TTMD) and any relevant secondaries will be performed after registration and diagnosis of the first 100 patients and after 500 patients. There are no

For peer review only - http://bmjopen.bmj.com/site/about/guidelines.xhtml

formal stopping rules. Formal analyses of all study outcome measures will be performed once the study has completed recruitment (target 1000 patients) and completed the follow up for all registered patients.

The analysis of TTMD will essentially be descriptive. The median TTMD will be reported overall and for each centre, together with the proportion of patients achieving TTMD within 28 days. Graphs of the change in both these summary statistics over time will be used to explore if TTMD changes during the course of the study. All estimates will be accompanied by 95% confidence intervals (CI). The time to completion of each node of tissue and imaging pathway will be reported as medians together with 95% CI, both overall and for each centre. Swimmer plots will be used to depict overall and node level timings for each patient.

For each type of tumour and biological sample, the proportion of sample passing QC and successfully undergoing WGS and EC will be reported with 95% CI and similarly for the imaging QC outcome measures. Inter-rater agreement of RANO will be assessed through Kendall's coefficient of concordance.

Extent of surgical resection is evaluated from the post-operative MRI scan and is categorised as either; closed biopsy, open biopsy, debulking <50%, subtotal resection 50-90%, near total resection 90-<100%, gross total resection 100%. The extent of surgical resection will be reported as the proportion falling into each category together with 95% CI.

OS is defined as the time from date of diagnosis to the date of death with patients who are alive at the time of analysis censored at the date last seen in clinic. Intracranial PFS time is defined as the time from date of registration to the earliest of date of intracranial progressive disease or death from disease. The date of an event is defined as the earliest confirmation of progression by radiological assessment, clinical symptoms or multidisciplinary team (MDT). Patients without progression at the time of analysis will be censored at the date last seen in clinic.

OS and intracranial PFS will be analysed and plotted using the Kaplan-Meier method. Median times with corresponding 95% CI will be reported together with rates at 1, 2 and 5 years. Multivariable survival regression modelling will be used to explore prognostic factors, including, but not limited to, WHO 2021 classification, age, tumour volume, stage, methylation and mutation status.

Longitudinal measures of QoL will be generated from the QoL questionnaire according to the questionnaire-specific algorithms for scoring. The analysis of longitudinal QoL scores will essentially be descriptive. For each of the multiple QoL scores generated from the different questionnaires, the means, medians or proportions (as appropriate) will be plotted over time together with 95% CI. These repeated measures over time may be modelled, if appropriate, with a linear or more flexible mixed model that takes account of the within-subject correlation and will allow exploration of factors associated with the outcome.

Details of the type of intervention received and complications (e.g., surgical wound infection) relating to standard of care treatments received will be monitored and recorded throughout the follow-up period and reported descriptively as frequencies and associated percentages. In relation to initial local logical diagnosis, local pathological diagnosis and integrated histological-molecular diagnosis, any difference between the tiers of diagnoses will be highlighted and categorised as: discordant; agreed; refined and reported descriptively as frequencies and associated percentages.

Confirmation of central storage of images and material, relevant targetable mutations identified by WGS and epigenomic classification, and receipt of post-mortem consent forms with confirmed central storage of samples will be recorded and reported descriptively.

For peer review only

Planned subgroup analyses of outcome measures include: IDH mutated and wildtype tumours; residual enhancing disease (RED; none, operable, inoperable); methylated and un-methylated tumours; age groups; types of diagnosis (WHO 2021 criteria only, epigenetic classification only, WGS analysis only, integrated diagnosis comprising all three); performance status; sex, and; biomarkers that emerge during the study (either discovered in the TJBM study or reported in the literature) that are deemed relevant after review by the Scientific Advisory Board (SAB).

## Biological samples

### Sample collection

Biological samples will be collected at each participating site following agreed protocols and guidance from the central biospecimen coordination centre (Oxford BRAIN MATRIX Laboratory). The aim is to build as much as possible on existing GEL infrastructure and pathways. The funded pathway is represented in Figure 3.

Fresh tissue for TJBM must be frozen in liquid nitrogen and stored until shipment to the Oxford BRAIN MATRIX Laboratory. Matched 'germline' DNA from white blood cells is required for the detection of somatic variants for paired blood/tumour WGS. This should be collected as per standard GEL protocols. Ideally, it should be collected prior to or at the time of first surgery. The blood sample(s) must be shipped together with the frozen tissue of the patient to the Oxford BRAIN MATRIX Laboratory.

For patients who are not undergoing surgery and have available tumour samples from previous tumour surgery, blood should be collected and sent to the Oxford BRAIN MATRIX Laboratory along with their tumour samples.

For participants unable to donate a blood sample, a saliva sample is an acceptable alternative.

Where possible, it is intended to collect blood samples for liquid biopsies of the tumour (e.g., tracking circulating tumour cell free (cft)DNA within the blood). Samples should ideally be collected at the time of operation, before the neurosurgeon performs any incision, ideally in theatres or the anaesthetics room just before any biopsy. Where feasible further blood samples should be collected at the following key treatment milestones:

1. At first post-operative MRI
2. Initiation of concomitant therapy (if applicable).
3. The end of concomitant therapy (if applicable).
4. Initiation of adjuvant therapy (if applicable).
5. The end of adjuvant therapy (if applicable).
6. The time of objectively measured progression.
7. During the palliative phase if a post-mortem has been agreed.

Where cerebrospinal fluid (CSF) is available for the patient, this should also be submitted.

Participating sites should follow the CRUK PEACE study protocol for post-mortem neurological tissue donation. The aim of post-mortem tissue donation is to enable research into:

1. The clonal evolution of the glioma after emergence of therapy resistance.
2. Tumour host-interaction at the whole brain level.
3. The effect of radio-chemotherapy on normal brain.

For peer review only

4. The interaction between the glioma and non-CNS organs (e.g., immune system in the cervical lymph nodes).

**Sample analyses**

Matched tumour/blood samples collected at first surgery are the most important samples of the TJBM study as these determine the initial integrated histological-molecular diagnosis. Following histological QC, the Oxford BRAIN MATRIX Laboratory will perform DNA extraction and QC; an aliquot will be sent to the Illumina Centre in Cambridge for WGS, and one used for EPIC methylation array at the Wellcome Trust Centre for Human Genetics in Oxford. Remaining DNA and unused tissues will be stored at the BRAIN MATRIX Biorepository.

Raw WGS data will be maintained in the GEL Data Research Environment, where it can be accessed and re-analysed by data scientists at the submitting NHS Genomic Laboratory Hub or any qualifying TJBM approved researcher. Sample data not generated by GEL will be consolidated in established bioinformatics hubs of the University of Oxford (Big Data Institute/Weatherall Institute for Molecular Medicine).

Data files on the Illumina 850k EPIC BeadChip analysis will be uploaded to the 'Heidelberg Classifier' hub at the German Cancer Centre in Heidelberg (27). This will generate an automated classifier report which will be stored at the Oxford BRAIN MATRIX Lab. Raw array data will be made available via the Oxford BRAIN MATRIX Laboratory.

Matched histological sections will be digitised at the Oxford BRAIN MATRIX Laboratory resulting in linked genomic data, which will initially be stored in Oxford. As the study evolves, we will aim to capture digital histological data from the material kept at the local neuropathology centre. The Oxford BRAIN MATRIX hub is working with CRUK-established data visualisation platforms, such as those developed for S:CORT (Colorectal Cancer) (28) and based on international open access platforms (such as cBioPortal (29)).

The BRAIN MATRIX neuropathology and genomics team will generate an integrated report (histology, WGS, Heidelberg Classifier) for each case in consultation with the local neuropathology team. The primary BRAIN MATRIX report will comprise the formal routine GEL WGS report (germline and tumour) and Heidelberg Classifier report and integrate this with the histological and molecular report issued by the recruiting site. It is anticipated that a local histological and molecular diagnosis using immunohistochemical surrogate markers and targeted genetic analyses will be available before WGS and Illumina EPIC BeadChip data are returned to the Oxford BRAIN MATRIX Lab. When available, the BRAIN MATRIX neuropathology and molecular genetics team will conduct a virtual MDT with the referring site to ensure all relevant information will be incorporated in the final BRAIN MATRIX diagnostic report. Variant calling and classifier outputs will be determined by the practice at the GEL/Illumina Centre and the version of the Heidelberg Classifier algorithm active at the time of sample analysis.

Where relevant germline data is identified, local sites should facilitate local genetic referral as per other GEL study protocols.

## Imaging data

Pseudo-anonymised longitudinal clinical imaging (MRI) for each patient will be collected and stored at a central imaging hub overseen by the Edinburgh Imaging Hub. Disease response assessment will be performed by practicing UK neuro-radiologists with a neuro-oncology interest via the Edinburgh Imaging Platform and additional analyses undertaken through the University of Edinburgh image analysis laboratory with permitted partners.

For peer review only - http://bmjopen.bmj.com/site/about/guidelines.xhtml

As per patient standard of care, it is expected that imaging will be performed pre- and post-operatively, for radiotherapy planning, following any chemoradiation, and as per follow-up determined by the managing MDT and/or if clinical concerns are raised regarding disease progression.

Pragmatic MRI protocols will be conducted as per standard of care MRI protocols and timing following diagnosis and as such will be informed by the National Institute for health and Care Excellence (NICE) guidelines from 2018 (30), in line with the recent British Society of Neuroradiologists (BSNR) imaging guidance (31), which is itself an implementation of the Brain Tumour Imaging Protocol proposed by Ellingson *et al.* (32). Where additional advanced imaging is performed this is also encouraged to be submitted to the Edinburgh Imaging Hub and will be catalogued to permit any relevant subsequent analysis. Biopsy location imaging should also be submitted, and standard operating protocols for the major neuro-navigation systems followed. Radiotherapy planning imaging will also be submitted.

The RANO assessments will be performed through a secure web portal provided by QMENTA Inc. There will be an ongoing 10% re-read to assess inter-rater agreement. It is anticipated that RANO reports will be provided within 2 weeks (10 working days) of successful transfer of imaging requiring assessment. RANO reports will categorise response where possible into Complete Response (CR), Partial Response (PR), Stable Disease (SD) or Progressive Disease (PD). In cases of diagnostic uncertainty, potential or provisional outcomes may be recorded, allowing progression events to be backdated to the correct time point should subsequent imaging be confirmatory.

## Adverse events reporting

There are no study treatments within the TJBM study. Blood sampling and completion of QoL questionnaires are the only procedures that patients undergo additional to usual care. Therefore, it is not anticipated that there will be adverse events related to participation in this study. Only severe adverse events (SAEs) relating to those additional procedures will be reported as per Common Terminology Criteria for Adverse Events (CTCAE) version 4 (33) and as defined in Supplementary Appendix 7. The reporting period is from the date of informed consent to death.

## Data management

Case report forms will be entered online via a secure web-based portal. Authorised staff at sites will require an individual secure login username and password to access this online data entry system. Paper CRFs will be available for backup only and must be completed, signed/dated and returned to the BRAIN MATRIX Study Office by the investigator or an authorised member of the site research team. Data reported on each CRF should be consistent with the source data or the discrepancies should be explained. If information is not known, this must be clearly indicated on the CRF. All missing and ambiguous data will be queried. All sections are to be completed.

All study records must be archived and securely retained for at least 25 years. No documents will be destroyed without prior approval from the sponsor, via the central Study Office. On-site monitoring will be carried out as required following a risk assessment and as documented in the Quality Management Plan. Any monitoring activities will be reported to the central BRAIN MATRIX Study Office and any issues noted will be followed up to resolution. BRAIN MATRIX will also be centrally monitored, which may trigger additional on-site monitoring. Further information regarding data management is provided in the study protocol.

For peer review only

The CRCTU will hold the final study dataset and will be responsible for the controlled sharing of anonymised data with the wider research community to maximise potential patient benefit while protecting the privacy and confidentiality of study participants. Data anonymised in compliance with the Information Commissioners Office requirements, using a procedure based on guidelines from the Medical Research Council (MRC) Methodology Hubs, will be available for sharing with researchers outside of the trials team within 12 months of the primary publication.

## Trial organisation structure

The University of Birmingham will act as single sponsor to this multi-centre study: Support Group, Aston Webb Building, Room 119, Birmingham, B15 2TT. Email: researchgovernance@contacts.bham.ac.uk). The study is being conducted under the auspices of the CRCTU, University of Birmingham according to their local procedures.

The Chief Investigator, Co-investigators, Trial Management Team Leader, Senior Trial Coordinator, Trial Coordinator, Lead and Trial Statistician, Trial Monitor and patient representatives will form the Study Management Group (SMG; current membership is listed in Supplementary Appendix 1). The SMG will be responsible for the day-to-day conduct of the TJBM study, meeting at regular intervals (e.g., at least every three months), or as required, usually by teleconference. They will be responsible for the set-up, promotion, on-going management of the study, the interpretation of the results and preparation and presentation of relevant publications.

Selected findings of clinical significance will be presented to the Scientific Advisory Board (SAB), which as a minimum will include the Chief Investigator, an oncologist, a pathologist, and a molecular biologist for a combined review of the molecular findings in context. The SAB may suggest amendments that will be incorporated into a SAB Report, which will be sent to the Executive Oversight Committee (EOC); current SAB and EOC membership is listed in Supplementary Appendix 1. Possible re-testing/further testing may be required, as a result of SAB feedback. Guided decision-making tools that review results in the context of the literature and clinical experience will be piloted within the SAB. The SAB will also evaluate research and study proposals, manage data/tissue requests, and will report back to the EOC regarding any proposals or changes which they may suggest.

The overarching remit of the EOC is to mandate, including timeframe and deliverables, the responsibility of the SAB and to take responsibility of horizon scanning to enable the incorporation of new interventional arms. They will oversee the overall study management of both the platform and any standalone interventional trials. They will also liaise with other trial units and pharma stakeholders as well as funders, charities, study sponsors, and policymakers and liaise with the BRIAN team. A quarterly EOC Report will be disseminated to all stakeholders that will demonstrate the performance metrics of each clinical site. In addition, the use of all samples given to external researchers via the SAB will be included in the report.

## Confidentiality

Confidential information collected during the study will be stored in accordance with the General Data Protection Regulation (GDPR) 2018. As specified in the patient information sheet (PIS) and with the patients' consent, patients will be identified using only their date of birth and unique study ID number. Authorised staff may have access to the records for quality assurance and audit purposes. The BRAIN MATRIX Study Office maintains the confidentiality of all patients' data and will not disclose information by which patients may be identified to any third party other than those directly involved in the treatment of

For peer review only

the patient and organisations for which the patient has given explicit consent for data transfer (e.g., laboratory staff).

### Trial status

Recruitment for the study opened in Nov-2020 and recruitment is expected to last for 5 years.

## Ethics and dissemination

The study will be performed in accordance with the recommendations guiding physicians in biomedical research involving human subjects, adopted by the 18th World Medical Association General Assembly, Helsinki, Finland and stated in the respective participating countries laws governing human research, and Good Clinical Practice. The protocol was initially approved on 18-Feb-2020 by West Midlands - Edgbaston Research Ethics Committee; the current protocol (v3.0) was approved on 15-Jun-2022.

A meeting will be held after the end of the study to allow discussion of the main results among the collaborators prior to publication. The results of this study will be disseminated through national and international presentations and peer-reviewed publications.

Manuscripts will be prepared by the SMG and authorship will be determined by mutual agreement.

## Discussion

### Justification for patient population

The main aim of the TJBM study is to test the hypothesis that comprehensive genomic and epigenomic profiling of gliomas is feasible in a timely manner in the UK, and that the results improve stratification of patients for next generation (targeted) therapies, ultimately improving outcomes and QoL.

Gliomas occur at all ages and their specific subtype is difficult to predict preoperatively. Therefore, the patient population eligible for the TJBM study is broad. It includes any patient who on preoperative assessment is suspected to have a diffuse glioma, or where a diffuse glioma remains a credible differential diagnosis, as established by the multidisciplinary team at the recruiting site.

Recruitment is also open to patients with diffuse or atypical gliomas who had a biopsy before the launch of the TJBM study. This approach ensures that:

1. Patients with slow-growing diffuse gliomas that evolve over many years (oligodendrogliomas) may benefit from one or more of the clinical trials developed as part of the TJBM study and sample collection at tumour progression.
2. Patients who potentially benefit most from comprehensive molecular diagnostics are not missed, which often are those with rare or atypical variants of glioma based on current diagnostics (34).

The infrastructure for children with brain tumours in the UK is different to that of adults with epigenomic classification regularly used and with WGS shortly to be made available for all children with cancer. Thus, the challenges and opportunities are different for paediatric patients. Nevertheless, it is intended that children will be included within the TJBM study wherever appropriate. The recruitment of children and adolescents into the TJBM study will be coordinated with the UK's paediatric oncology expert group(s).

For peer review only

## Justification for methodology

Neuroimaging with MRI plays a central role in the initial diagnosis and treatment stratification, surgical and radiotherapy planning and assessment of disease response and progression in glioma.

Potential participants will have undergone MRI as part of a diagnostic work-up and an intracranial mass identified. As per current clinical practice, expert neuroradiological review will indicate glioma as the likely diagnosis, and a plan for treatment will be made by the relevant regional MDT meeting. Those with suspected glioma on MRI who are to undergo surgery and are otherwise eligible for the TJBM study will be approached. A proportion of these lesions that are subsequently diagnosed as types of brain tumours other than glioma will inevitably be included. They will not be included in the main analysis; however, data from these will be stored for additional research into those less common tumours which can be challenging to diagnose from imaging alone.

At the time of surgery, neuro-navigation will be used to capture the location of each biopsy taken for diagnosis and further molecular characterisation within the TJBM study. This will aid with subsequent analysis of tumour molecular markers and heterogeneity in the context of radio-genomics – a recent development in cancer imaging for assessment of disease in the personalised medicine era employing machine learning and artificial intelligence approaches (32).

Early post-operative imaging for suspected HGG, including pre- and post-contrast T1-weighted MRI, is currently recommended within 72 hours of surgery to minimise the presence of non-neoplastic enhancement. This imaging will be captured to permit eventual assessment of extent of resection, or the volume of residual enhancing disease, important prognostic factors for glioma. Subsequent to this, any additional imaging for radiotherapy planning, including computerised tomography (CT), will be captured. Under the most recent response assessment in neuro-oncology (34) recommendations for HGG from 2017, this is recommended as the baseline for assessing treatment response (32).

All imaging will receive primary radiological reports at the local site as per current standard clinical practice. To deliver the imaging outcome requirements for clinical trials, centralised RANO reads will be delivered by a core group of UK Consultant neuro-radiologists with a subspecialty interest in neuro-oncology imaging from TJBM study sites. Radiotherapy planning data in the form of dose distribution maps and associated data will be integrated into the imaging database to permit subsequent analysis of spatiotemporal response patterns of different glioma-treatment combinations in light of received radiation dose.

Local radiological reporting will inform clinical decision making for individual patients. Central image analysis will provide resilient standardised assessment to meet internationally-accepted standards which are suitable for peer-reviewed publication and are accepted by major regulatory approval bodies. All relevant pre-operative and subsequent imaging will be identified for pseudonymisation and uploaded to the BRAIN MATRIX Imaging Platform in Edinburgh. This will be achieved through a dedicated online secure portal, with alternative secure online and physical transmission pathways available for redundancy. The accrued imaging data will form a core resource that will be leveraged for future imaging and clinical radiological research. The platform can also provide the basis for additional imaging studies within non-standard of care imaging and advanced techniques, which would be separately funded and detailed in their respective project documentation.

For peer review only

## Justification for tissue collection

Historical approaches to brain tumour tissue collection in formaldehyde and paraffin are not currently fully compatible with modern genomic technologies, which require frozen tissue. This is why the collection of fresh frozen (FF) material is essential for the TJBM study. Pairing with non-neoplastic, so-called germline DNA, is also essential for confident calling of somatic variants in the tumour. Germline DNA analysis will also provide novel data on genetic risk for glioma predisposition.

Blood plasma, where possible, will be collected to facilitate future analysis of cftDNA. This technology is still in its infancy; however, it is clear that non-invasive, real-time monitoring of tumour evolution will become feasible in the next 5-10 years (35). Similarly, where available for the patient, CSF will be submitted.

Post-mortem tissue banking via the CRUK PEACE and MRC Brain BioLink projects will allow us to study the glioma-brain interface, extent of spatial tumour heterogeneity, genomic signature of the treatment resistant tumour clones, and effect of treatments on normal brain. Systematic post-mortem brain banking for research into adult gliomas does not currently exist in the UK.

## Justification for molecular diagnostics

All TJBM study baseline diagnoses will follow the new WHO classification of tumours of the CNS from 2021 to achieve an integrated histological-molecular diagnosis for diffuse gliomas (10). This is achieved with a combination of immunohistochemical surrogate markers and – in most instances – targeted or panel sequencing for relevant hotspot mutations, or cytogenetics (7). Only in exceptional circumstances unbiased 'omics' approaches are used, such as the epigenomic 'Heidelberg Classifier' (34). Further, any diagnostic approach may differ between centres in the UK, making analyses of cohorts pooled from different sites difficult.

The new WHO 2021 classification represents a consensus opinion based on new insights into the molecular definition of brain tumours from cIMPACT-NOW. cIMPACT-NOW updates are not intended to supplant the existing WHO classification, but to provide possible guidelines for practicing diagnosticians and future WHO classification updates. It is clear from the first iterations of cIMPACT-NOW that any progress is driven by next generation ('omics') molecular analysis, not by standard or targeted analyses. This insight underpins the selection of molecular analytical tools for the TJBM platform, namely, combined paired (blood-tumour) WGS DNA analysis integrated with the epigenomic 'Heidelberg Classifier' (34). To achieve this, the TJBM study will build on the experience and UK infrastructure of the 100,000 genomes project led by GEL, which introduced WGS pathways into clinical practice. Early results from WGS in non-brain cancer patients suggest that virtually all patients could be mapped to existing or potential targeted therapies (36). Prospective large-scale paired WGS sequencing studies in diffuse glioma patients have not been done; however, experience from other brain tumours such as medulloblastoma, suggest that analysis of WGS data will provide new insights into glioma subtype diversity, including alterations in specific non-coding regulatory elements not evident from non-WGS genomic approaches (37). WGS data will capture all currently known relevant variants and uncover novel variants relevant for a better understanding of tumour evolution and response to treatment. WGS and epigenomic analyses are highly complementary genomic approaches (37): WGS will establish all potentially actionable mutations and epigenomic classification will establish an unbiased score for the precise classification of the glioma (34). Neither can be achieved with conventional targeted sequencing approaches. Importantly, the epigenomic classification by novel DNA methylation-based 'Heidelberg Classifier' has been shown to fundamentally alter clinical diagnoses and histological grades in >10% of biopsies, leading to changes in therapy (34). Moreover, the epigenomic array technology provides genomic copy number variant data, which in theory can also be

For peer review only - http://bmjopen.bmj.com/site/about/guidelines.xhtml

inferred from WGS data. However, pipelines for this type of analysis from WGS data are just evolving and it is predicted that comparative analysis of WGS and Illumina's EPIC BeadChip data will be bioinformatically highly valuable. Finally, WGS and the EPIC array raw data will form a unique source for researchers who will be able to access this data together with all clinical, imaging and histological data. Creating a relatively future-proof, quality-controlled research infrastructure is one of the main aims of the TJBM study, in addition to establishing feasibility of timely genomic diagnosis in the NHS setting.

the TJBM study is more than paired tumour/blood WGS and EPIC array analysis. As the former is being rolled out in the NHS in England, the BRAIN MATRIX molecular neuropathology team will work with GEL and other stakeholders (including industry) to explore the next-generation of tissue analytics (such as long-read sequencing and mass spectrometry). This will result in a unique, prospectively acquired dataset that will enable researchers to integrate data analysis across modalities and with outcomes and treatment response.

### Justification for patient outcome and quality of life

Clinical trials require prospective collection of clinical data to Good Clinical Practice (GCP) standards. This platform will develop the infrastructure for the collection of this, enabling streamlined patient recruitment into clinical trials. Evaluation of treatments and associated complications across patients will give an accurate measure of adverse events associated with current standard of care treatment in practice in the UK.

Maximising QoL for patients with diffuse glioma is important particularly given its poor prognosis; therefore, standardised measures of QoL will be collected. In adults these have been selected for their standardisation and ease of use and have been reviewed with input from Patient Public Involvement (PPI) representatives. It is intended that in the future integration with The Brain Tumour Charity's BRIAN project (REC Reference: 18/SC/0283) (19) will allow further understanding of patient reported outcome measures.

## Contributors

CW is the Chief Investigator and first author, responsible for conception and design of the study, drafting and review of paper. JS in the trial management team leader, responsible for study design, drafting and review of paper. AP is the study biostatistician, responsible for study design, drafting and review of paper. RM is the senior trial coordinator, responsible for review of paper. VW is the principal investigator at Birmingham, responsible for review of paper. UP is the neuropathologist at Birmingham, responsible for study design, and review of paper. HB is a study patient and public representative, responsible for study design and review of paper. JA is responsible for study design and review of paper. RS is responsible for design of the study and review of paper. GT is responsible for conception and design of the study, and review of paper. AW is responsible for conception and design of the study, and review of paper. OA is responsible for conception and design of the study, and review of paper. LB is the lead biostatistician, responsible for design of study, drafting and review of paper.

## Funding

This is an Investigator-initiated and Investigator-led study funded by The Brain Tumour Charity (GN-000580) and Genomics England.

For peer review only

## Role of funders and sponsor

Neither the sponsor nor funders had any role in study design, data collection, data analysis, data interpretation or writing of the report. The corresponding author had full access to all the data in the study and had final responsibility for the decision to submit for publication.

## Competing interests

All other authors declare no competing interests.

## Acknowledgements

The investigators and sponsor thank all the patients and their families who participated in this study, as well as the NHS Trusts and staff and the members of the Executive Oversight Committee and Scientific Advisory Board. Special thanks to our PPI representatives, Peter Buckle and Helen Bulbeck, who have ensured that the patient perspective has been incorporated into this study protocol. In addition, we thank staff past and present from the CRCTU, University of Birmingham including Clive Stubbs, Richard Fox, Dr Sarah Bowden, Prof. Christina Yap, Dr Siân Lax and Dr Louisa Jeffery, and Hannah Brooks at the University of Oxford for contributions to the protocol and paper. This study has been independently peer-reviewed and adopted into the National Institute for Health Research Clinical Research Network Portfolio.

Staff at the CRCTU are also supported by core funding grants from Cancer Research UK (C22436/A25354). JA is funded by an NIHR Academic Clinical Lectureship, with previous funded from a Cancer Research UK Clinical Trials Fellowship. This study was supported by Experimental Cancer Medicine Centres (ECMC) funding (Ref. 25127) and by the ECMC Network.

Genomics England is supported by funding from INNOVATE UK.

## References

1. Ostrom QT, Gittleman H, Fulop J, Liu M, Blanda R, Kromer C, et al. CBTRUS Statistical Report: Primary Brain and Central Nervous System Tumors Diagnosed in the United States in 2008-2012. Neuro Oncol. 2015;17 Suppl 4:iv1-iv62.

2. Cancer Research UK. Brain, other CNS and intracranial tumour statistics [Available from: https://www.cancerresearchuk.org/health-professional/cancer-statistics/statistics-by-cancer-type/brain-other-cns-and-intracranial-tumours.

3. Rouse C, Gittleman H, Ostrom QT, Kruchko C, Barnholtz-Sloan JS. Years of potential life lost for brain and CNS tumors relative to other cancers in adults in the United States, 2010. Neuro Oncol. 2016;18(1):70-7.

4. Rachet B, Mitry E, Quinn MJ, Cooper N, Coleman MP. Survival from brain tumours in England and Wales up to 2001. Br J Cancer. 2008;99 Suppl 1:S98-101.

5. Louis DN, Perry A, Reifenberger G, von Deimling A, Figarella-Branger D, Cavenee WK, et al. The 2016 World Health Organization Classification of Tumors of the Central Nervous System: a summary. Acta Neuropathol. 2016;131(6):803-20.

6. Sanai N, Chang S, Berger MS. Low-grade gliomas in adults. J Neurosurg. 2011;115(5):948-65.

7. Stupp R, Mason WP, van den Bent MJ, Weller M, Fisher B, Taphoorn MJB, et al. Radiotherapy plus Concomitant and Adjuvant Temozolomide for Glioblastoma. New England Journal of Medicine. 2005;352(10):987-96.

8. Weller M, Wick W, Aldape K, Brada M, Berger M, Pfister SM, et al. Glioma. Nature Reviews Disease Primers. 2015;1(1):15017.

For peer review only - http://bmjopen.bmj.com/site/about/guidelines.xhtml

9. The International Society of Neuropathology. cIMPACT-NOW 2019 [Available from: http://www.intsocneuropathol.com/2019/03/cimpact-now/.

10. Louis DN, Perry A, Wesseling P, Brat DJ, Cree IA, Figarella-Branger D, et al. The 2021 WHO Classification of Tumors of the Central Nervous System: a summary. Neuro-Oncology. 2021;23(8):1231-51.

11. 100,000 Genomes Pilot on Rare-Disease Diagnosis in Health Care — Preliminary Report. New England Journal of Medicine. 2021;385(20):1868-80.

12. UK Biobank 2022 [Available from: https://www.ukbiobank.ac.uk/.

13. MRC Brain Banks Network [Available from: https://directory.biobankinguk.org/Profile/Biobank/GBR-1-34.

14. The PEACE (Posthumous Evaluation of Advanced Cancer Environment) Study 2016 [Available from: https://clinicaltrials.gov/ct2/show/NCT03004755.

15. Aldape K, Amin SB, Ashley DM, Barnholtz-Sloan JS, Bates AJ, Beroukhim R, et al. Glioma through the looking GLASS : Molecular evolution of diffuse gliomas and the Glioma Longitudinal Analysis Consortium. Neuro-oncology. 2018;20(7):11.

16. EURACAN. European Network for Rare adult solid Cancer [Available from: http://euracan.ern-net.eu/.

17. Medical Research Council. Tariffs for Brain Tissue 2021 [Available from: https://webarchive.nationalarchives.gov.uk/ukgwa/20210903230131/https://mrc.ukri.org/research/facilities-and-resources-for-researchers/brain-banks/tariffs-for-brain-tissue/.

18. Chan A-W, Tetzlaff JM, Gøtzsche PC, Altman DG, Mann H, Berlin JA, et al. SPIRIT 2013 explanation and elaboration: guidance for protocols of clinical trials. BMJ : British Medical Journal. 2013;346:e7586.

19. The Brain Tumour Charity. BRIAN [Available from: https://www.thebraintumourcharity.org/living-with-a-brain-tumour/brian/.

20. Lyden P, Brott T, Tilley B, Welch KM, Mascha EJ, Levine S, et al. Improved reliability of the NIH Stroke Scale using video training. NINDS TPA Stroke Study Group. Stroke. 1994;25(11):2220-6.

21. Group TE. EuroQol - a new facility for the measurement of health-related quality of life. Health Policy. 1990;16(3):199-208.

22. Aaronson NK, Ahmedzai S, Bergman B, Bullinger M, Cull A, Duez NJ, et al. The European Organization for Research and Treatment of Cancer QLQ-C30: A Quality-of-Life Instrument for Use in International Clinical Trials in Oncology. Journal of the National Cancer Institute. 1993;85(5):365-76.

23. Rogers SN, El-Sheikha J, Lowe D. The development of a Patients Concerns Inventory (PCI) to help reveal patients concerns in the head and neck clinic. Oral Oncol. 2009;45(7):555-61.

24. Guy W, National Institute of Mental H, Psychopharmacology Research B, Early Clinical Drug Evaluation P. ECDEU assessment manual for psychopharmacology. Rockville, Md.: U.S. Dept. of Health, Education, and Welfare, Public Health Service, Alcohol, Drug Abuse, and Mental Health Administration, National Institute of Mental Health, Psychopharmacology Research Branch, Division of Extramural Research Programs; 1976.

25. Busner J, Targum SD. The clinical global impressions scale: applying a research tool in clinical practice. Psychiatry (Edgmont). 2007;4(7):28-37.

26. Chukwueke UN, Wen PY. Use of the Response Assessment in Neuro-Oncology (RANO) criteria in clinical trials and clinical practice. CNS Oncol. 2019;8(1):CNS28-CNS.

27. Heidelberg Classifier. The platform for next generation neuropathology [Available from: www.molecularneuropathology.org/mnp.

28. S:CORT: Colorectal Cancer Detection & Diganosis Novel Treatments 2022 [Available from: https://www.cancer.ox.ac.uk/research/networks/scort.

29. cBioPortal for Cancer Genomics [Available from: https://www.cbioportal.org/.

30. National Institute for Health and Care Excellence. Brain tumours (primary) and brain metastases in over 16s 2018 [updated 29-Apr-2022. Available from: https://www.nice.org.uk/guidance/ng99.

For peer review only

31. British Society of Neuroradiologists. Core imaging protocol for brain tumours 2018 [Available from: https://bsnr.org.uk/wp-content/uploads/2021/11/bsnrstandardsbraintumour.pdf.

32. Ellingson BM. Radiogenomics and Imaging Phenotypes in Glioblastoma: Novel Observations and Correlation with Molecular Characteristics. Current Neurology and Neuroscience Reports. 2014;15(1):506.

33. Cancer Therapy Evaluation Program. Common Terminology Criteria for Adverse Events (CTCAE), Version 4.03 2010 [Available from: https://ctep.cancer.gov/protocoldevelopment/electronic_applications/ctc.htm#ctc_40.

34. Capper D, Jones DTW, Sill M, Hovestadt V, Schrimpf D, Sturm D, et al. DNA methylation-based classification of central nervous system tumours. Nature. 2018;555:469.

35. De Rubis G, Rajeev Krishnan S, Bebawy M. Liquid Biopsies in Cancer Diagnosis, Monitoring, and Prognosis. Trends in Pharmacological Sciences. 2019;40(3):172-86.

36. Schuh A, Dreau H, Knight SJL, Ridout K, Mizani T, Vavoulis D, et al. Clinically actionable mutation profiles in patients with cancer identified by whole-genome sequencing. Cold Spring Harb Mol Case Stud. 2018;4(2):a002279.

37. Northcott PA, Buchhalter I, Morrissy AS, Hovestadt V, Weischenfeldt J, Ehrenberger T, et al. The whole-genome landscape of medulloblastoma subtypes. Nature. 2017;547:311.

# Figure Legends

## Figure 1: Overview of the Tessa Jowell BRAIN MATRIX Programme

The Tessa Jowell BRAIN MATRIX Platform (TJBM) study will collect and integrate clinical, pathological, advanced molecular, imaging, quality of life, treatment and outcome data. The platform may provide data directly or support identification of eligible patients to clinical trials, within and outside the TJBM study programme. If eligible, patients may be enrolled in multiple add-on studies. Through consent and with strong governance processes, anonymised or pseudonymised data may be shared with other relevant organisations or studies within and outside the programme.

## Figure 2: The Tessa Jowell BRAIN MATRIX Platform study schema

The study schema for the Tessa Jowell BRAIN MATRIX Platform study.

## Figure 3: The Tessa Jowell BRAIN MATRIX Platform study sample and data flow pathways

The sample and data flow pathways within the Tessa Jowell BRAIN MATRIX Platform (TJBM) study.

*TJBM sites in England are encouraged to route all samples through their local NHS GMS GLH, however, if this pathway is not yet activated or GEL consent cannot be obtained, the TJBM Study Office can facilitate the processing of samples through an alternative NHS GMS GLH or via the GEL Research pathway.

GEL, Genomics England; GLH, Genomic Laboratory Hub; GMS, Genomic Medicine Service; GTAB, Genomics Tumour Advisory Board; NHS, National Health Service; WGS, Whole Genome Sequencing.

For peer review only

## Additional Files

### Supplementary Appendix 1: Tessa Jowell BRAIN MATRIX Platform Study Investigators & Committee Membership

Principal Investigators, Study Management Group (SMG), Executive Oversight Committee (EOC) and Scientific Advisory Board (SAB).

### Supplementary Appendix 2: The Tessa Jowell BRAIN MATRIX Platform study SPIRIT Checklist

A completed Standard Protocol Items: Recommendations for Intervention Trials (SPIRIT) checklist for the Tessa Jowell BRAIN MATRIX Platform study protocol.

### Supplementary Appendix 3: The Tessa Jowell BRAIN MATRIX Platform study World Health Organization Trial Registration Data Set

The World Health Organization (WHO) trial registration data set for the Tessa Jowell BRAIN MATRIX Platform study.

### Supplementary Appendix 4: The Tessa Jowell BRAIN MATRIX Platform study informed consent form

The informed consent form for the Tessa Jowell BRAIN MATRIX Platform study.

### Supplementary Appendix 5: The Tessa Jowell BRAIN MATRIX Platform study lay summary and patient information sheet

The lay summary and patient information sheet for the Tessa Jowell BRAIN MATRIX Platform study.

### Supplementary Appendix 6: The Tessa Jowell BRAIN MATRIX Platform study schedule of events

Patient schedule of events for the Tessa Jowell BRAIN MATRIX Platform study.

For peer review only

727 # Supplementary Appendix 7: Definition of adverse events

728 Definitions of adverse events used for the Tessa Jowell BRAIN MATRIX Platform study.

729

For peer review only

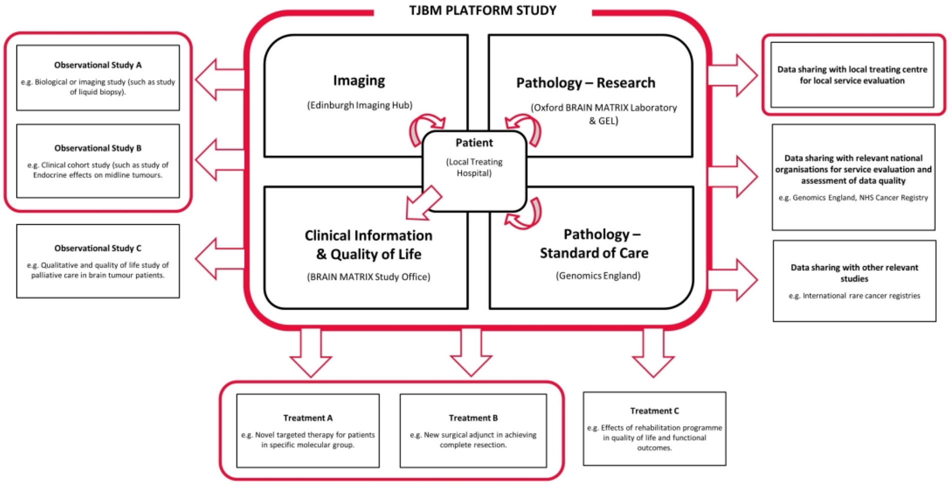


The Tessa Jowell BRAIN MATRIX Platform (TJBM) study will collect and integrate clinical, pathological, advanced molecular, imaging, quality of life, treatment and outcome data. The platform may provide data directly or support identification of eligible patients to clinical trials, within and outside the TJBM study programme. If eligible, patients may be enrolled in multiple add-on studies. Through consent and with strong governance processes, anonymised or pseudonymised data may be shared with other relevant organisations or studies within and outside the programme.

338x190mm (300 x 300 DPI)

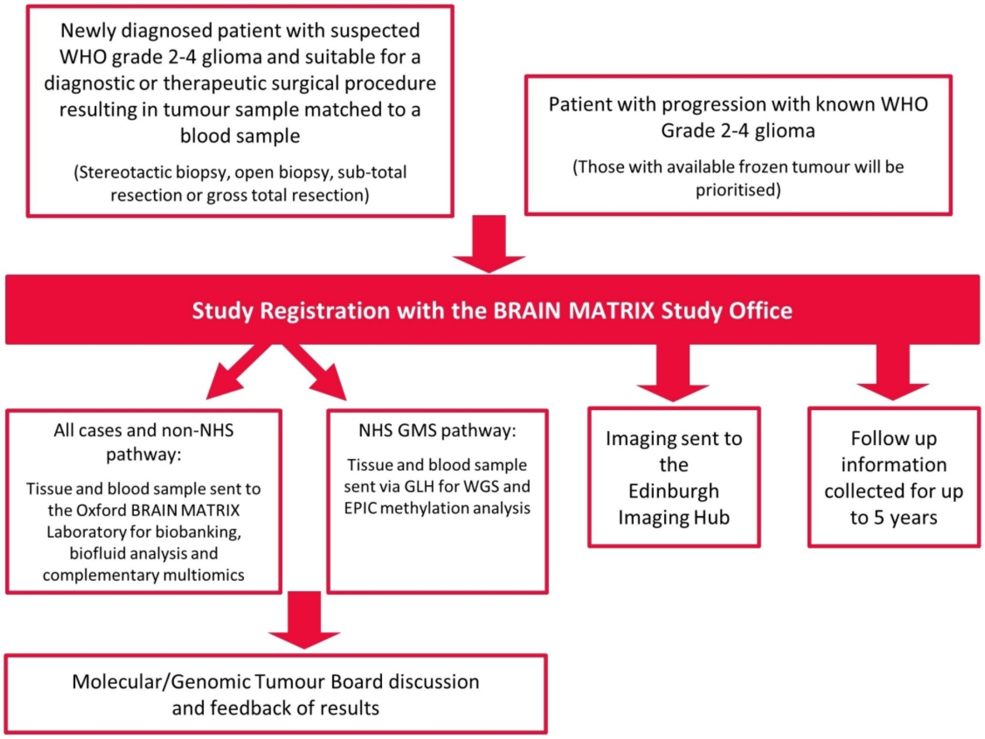


The study schema for the Tessa Jowell BRAIN MATRIX Platform study.

176x133mm (300 x 300 DPI)

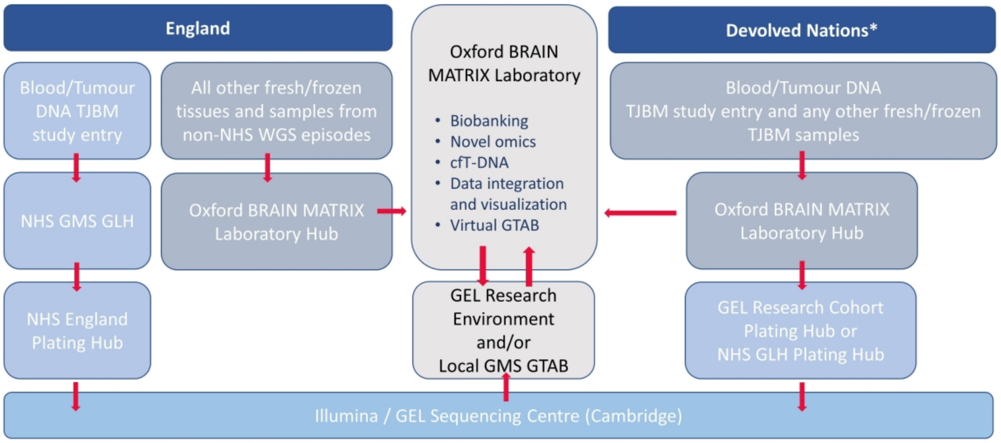


The sample and data flow pathways within the Tessa Jowell BRAIN MATRIX Platform (TJBM) study. *TJBM sites in England are encouraged to route all samples through their local NHS GMS GLH, however, if this pathway is not yet activated or GEL consent cannot be obtained, the TJBM Study Office can facilitate the processing of samples through an alternative NHS GMS GLH or via the GEL Research pathway. GEL, Genomics England; GLH, Genomic Laboratory Hub; GMS, Genomic Medicine Service; GTAB, Genomics Tumour Advisory Board; NHS, National Health Service; WGS, Whole Genome Sequencing.

323x146mm (300 x 300 DPI)

# Supplementary Appendix 1: Tessa Jowell BRAIN MATRIX Platform Study Investigators & Committee Membership

## Principal Investigators

- Dr Victoria Wykes - Queen Elizabeth Hospital, Birmingham
- Prof. Keyoumars Ashkan - King's College Hospital, London
- Prof. Michael Jenkinson - The Walton Centre, Liverpool
- Prof. Susan Short - St James's University Hospital. Leeds
- Dr Paul Brennan - NHS Lothian
- Dr Richard Mair - Addenbrooke's Hospital, Cambridge
- Dr Stuart Smith - Queens Medical Centre, Nottingham
- Prof. Anthony Chalmers - Greater Glasgow and Clyde
- Dr David Coope - Salford Royal Hospital, Manchester
- Dr Catherine McBain - The Christie, Manchester
- Dr Clare Hobbs - John Radcliffe Hospital, Oxford

## Study Management Group (SMG)

- Professor Colin Watts (Chief Investigator) - Institute of Cancer and Genomic Sciences (ICGS), University of Birmingham
- Professor Olaf Ansorge - University of Oxford
- Dr Ute Pohl - University Hospitals Birmingham
- Professor Adam Waldman - University of Edinburgh
- Dr Gerard Thompson - University of Edinburgh
- Dr John Apps - University of Birmingham
- Dr Victoria Wykes - University of Birmingham
- Professor Lucinda Billingham – CRCTU, University of Birmingham
- Dr Rowena Sharpe – CRCTU, University of Birmingham
- Amit Patel - CRCTU, University of Birmingham
- Dr Joshua Savage - CRCTU, University of Birmingham
- Rhys Mant - CRCTU, University of Birmingham
- Dr Louisa Jeffery - CRCTU, University of Birmingham
- Hannah Brooks – University of Oxford
- Peter Buckle (PPI Representative)
- Dr Helen Bulbeck (PPI Representative) - brainstrust

## Executive Oversight Committee (EOC)

- Professor Tim Maughan (Chair)– Professor of Clinical Oncology, CRUK/MRC Institute for Radiation Oncology, University of Oxford



For peer review only - http://bmjopen.bmj.com/site/about/guidelines.xhtml

- Professor David Cameron – Clinical Director of the Cancer Research UK Edinburgh Centre, University of Edinburgh
- Dr Helen Campbell – Portfolio Manager for Department of Health and Social Care Research Networks, Clinical Research Facilities, and Cancer Research, University of Exeter
- Dr Mark Gilbert – Chair of the Neuro-Oncology Branch, Centre for Cancer Research, National Cancer Institute
- Professor Richard Gilbertson – Director of CRUK Cambridge Centre
- Professor Max Parmar – Director of the MRC Clinical Trials Unit, UCL
- Lord James O'Shaughnessy – Member of House of Lords
- Professor Steven Pollard – Group Leader, MRC Centre for Regenerative Medicine and Edinburgh Cancer Research Centre, University of Edinburgh
- Jess Mills – Co-Founder and Special Advisor, Tessa Jowell Brain Cancer Mission

**Scientific Advisory Board (SAB)**

- Professor Ruth Plummer (Chair) - Professor of Experimental Cancer Medicine, Newcastle University
- Professor Neil Carragher - Professor of Drug Discovery, University of Edinburgh
- Professor Anthony Chalmers - Chair of Clinical Oncology, University of Glasgow
- Will Jones - Chief Executive, brainstrust
- Dr Juanita Lopez - Consultant Medical Oncologist, The Royal Marsden NHSFT
- Professor Whitney Pope – Director, Brain Tumour Imaging, UCLA
- Professor Thomas Wurdinger - Professor of Neurosurgery, Amsterdam UMC
- Professor Christina Yap - Professor of Clinical Trials Biostatistics, ICR
- Professor Patrick Wen - Professor of Neurology, Harvard Medical School
- Professor Roel Verhaak - Professor and Associate Director for Computational Biology, The Jackson Laboratory for Genomic Medicine

For peer review only



For peer review only - http://bmjopen.bmj.com/site/about/guidelines.xhtml

# Reporting checklist for protocol of a clinical trial.

Based on the SPIRIT guidelines.

## Instructions to authors

Complete this checklist by entering the page numbers from your manuscript where readers will find each of the items listed below.

Your article may not currently address all the items on the checklist. Please modify your text to include the missing information. If you are certain that an item does not apply, please write "n/a" and provide a short explanation.

Upload your completed checklist as an extra file when you submit to a journal.

In your methods section, say that you used the SPIRITreporting guidelines, and cite them as:

Chan A-W, Tetzlaff JM, Gøtzsche PC, Altman DG, Mann H, Berlin J, Dickersin K, Hróbjartsson A, Schulz KF, Parulekar WR, Krleža-Jerić K, Laupacis A, Moher D. SPIRIT 2013 Explanation and Elaboration: Guidance for protocols of clinical trials. BMJ. 2013;346:e7586

| | | Reporting Item | Page Number |
|---|---|---|---|
| **Administrative information** | | | |
| Title | #1 | Descriptive title identifying the study design, population, interventions, and, if applicable, trial acronym | 1 |
| Trial registration | #2a | Trial identifier and registry name. If not yet registered, name of intended registry | 2 |
| Trial registration: data set | #2b | All items from the World Health Organization Trial Registration Data Set | Appendix 3 |
| Protocol version | #3 | Date and version identifier | 12 |
| Funding | #4 | Sources and types of financial, material, and other support | 16 |

For peer review only

| | | | |
|---|---|---|---|
| Roles and responsibilities: contributorship | #5a | Names, affiliations, and roles of protocol contributors | 1 + 15-16 |
| Roles and responsibilities: sponsor contact information | #5b | Name and contact information for the trial sponsor | 11 |
| Roles and responsibilities: sponsor and funder | #5c | Role of study sponsor and funders, if any, in study design; collection, management, analysis, and interpretation of data; writing of the report; and the decision to submit the report for publication, including whether they will have ultimate authority over any of these activities | 16 |
| Roles and responsibilities: committees | #5d | Composition, roles, and responsibilities of the coordinating centre, steering committee, endpoint adjudication committee, data management team, and other individuals or groups overseeing the trial, if applicable (see Item 21a for data monitoring committee) | 11 |
| **Introduction** | | | |
| Background and rationale | #6a | Description of research question and justification for undertaking the trial, including summary of relevant studies (published and unpublished) examining benefits and harms for each intervention | 3-4 |
| Background and rationale: choice of comparators | #6b | Explanation for choice of comparators | n/a |
| Objectives | #7 | Specific objectives or hypotheses | 3-4 |
| Trial design | #8 | Description of trial design including type of trial (eg, parallel group, crossover, factorial, single group), allocation ratio, and framework (eg, superiority, equivalence, non-inferiority, exploratory) | 4 |
| **Methods: Participants,** | | | |

For peer review only

| | | | |
|---|---|---|---|
| **interventions, and outcomes** | | | |
| Study setting | #9 | Description of study settings (eg, community clinic, academic hospital) and list of countries where data will be collected. Reference to where list of study sites can be obtained | 4 |
| Eligibility criteria | #10 | Inclusion and exclusion criteria for participants. If applicable, eligibility criteria for study centres and individuals who will perform the interventions (eg, surgeons, psychotherapists) | 5 |
| Interventions: description | #11a | Interventions for each group with sufficient detail to allow replication, including how and when they will be administered | n/a |
| Interventions: modifications | #11b | Criteria for discontinuing or modifying allocated interventions for a given trial participant (eg, drug dose change in response to harms, participant request, or improving / worsening disease) | n/a |
| Interventions: adherance | #11c | Strategies to improve adherence to intervention protocols, and any procedures for monitoring adherence (eg, drug tablet return; laboratory tests) | n/a |
| Interventions: concomitant care | #11d | Relevant concomitant care and interventions that are permitted or prohibited during the trial | n/a |
| Outcomes | #12 | Primary, secondary, and other outcomes, including the specific measurement variable (eg, systolic blood pressure), analysis metric (eg, change from baseline, final value, time to event), method of aggregation (eg, median, proportion), and time point for each outcome. Explanation of the clinical relevance of chosen efficacy and harm outcomes is strongly recommended | 6 |
| Participant timeline | #13 | Time schedule of enrolment, interventions (including any run-ins and washouts), assessments, and visits for participants. A schematic diagram is highly recommended (see Figure) | Appendix 6 |

For peer review only

| | | | |
|---|---|---|---|
| Sample size | #14 | Estimated number of participants needed to achieve study objectives and how it was determined, including clinical and statistical assumptions supporting any sample size calculations | 6-7 |
| Recruitment | #15 | Strategies for achieving adequate participant enrolment to reach target sample size | n/a |
| **Methods: Assignment of interventions (for controlled trials)** | | | |
| Allocation: sequence generation | #16a | Method of generating the allocation sequence (eg, computer-generated random numbers), and list of any factors for stratification. To reduce predictability of a random sequence, details of any planned restriction (eg, blocking) should be provided in a separate document that is unavailable to those who enrol participants or assign interventions | n/a |
| Allocation concealment mechanism | #16b | Mechanism of implementing the allocation sequence (eg, central telephone; sequentially numbered, opaque, sealed envelopes), describing any steps to conceal the sequence until interventions are assigned | n/a |
| Allocation: implementation | #16c | Who will generate the allocation sequence, who will enrol participants, and who will assign participants to interventions | 5 |
| Blinding (masking) | #17a | Who will be blinded after assignment to interventions (eg, trial participants, care providers, outcome assessors, data analysts), and how | n/a |
| Blinding (masking): emergency unblinding | #17b | If blinded, circumstances under which unblinding is permissible, and procedure for revealing a participant's allocated intervention during the trial | n/a |
| **Methods: Data collection, management, and analysis** | | | |

For peer review only

| | | | |
|---|---|---|---|
| Data collection plan | #18a | Plans for assessment and collection of outcome, baseline, and other trial data, including any related processes to promote data quality (eg, duplicate measurements, training of assessors) and a description of study instruments (eg, questionnaires, laboratory tests) along with their reliability and validity, if known. Reference to where data collection forms can be found, if not in the protocol | 10 |
| Data collection plan: retention | #18b | Plans to promote participant retention and complete follow-up, including list of any outcome data to be collected for participants who discontinue or deviate from intervention protocols | n/a |
| Data management | #19 | Plans for data entry, coding, security, and storage, including any related processes to promote data quality (eg, double data entry; range checks for data values). Reference to where details of data management procedures can be found, if not in the protocol | 10 |
| Statistics: outcomes | #20a | Statistical methods for analysing primary and secondary outcomes. Reference to where other details of the statistical analysis plan can be found, if not in the protocol | 6-8 |
| Statistics: additional analyses | #20b | Methods for any additional analyses (eg, subgroup and adjusted analyses) | 8 |
| Statistics: analysis population and missing data | #20c | Definition of analysis population relating to protocol non-adherence (eg, as randomised analysis), and any statistical methods to handle missing data (eg, multiple imputation) | n/a |
| **Methods: Monitoring** | | | |
| Data monitoring: formal committee | #21a | Composition of data monitoring committee (DMC); summary of its role and reporting structure; statement of whether it is independent from the sponsor and competing interests; and reference to where further details about its charter can be found, if not in the | n/a |

For peer review only

| | | | |
|---|---|---|---|
| | | protocol. Alternatively, an explanation of why a DMC is not needed | |
| Data monitoring: interim analysis | #21b | Description of any interim analyses and stopping guidelines, including who will have access to these interim results and make the final decision to terminate the trial | 6-7 |
| Harms | #22 | Plans for collecting, assessing, reporting, and managing solicited and spontaneously reported adverse events and other unintended effects of trial interventions or trial conduct | 10 |
| Auditing | #23 | Frequency and procedures for auditing trial conduct, if any, and whether the process will be independent from investigators and the sponsor | n/a |
| **Ethics and dissemination** | | | |
| Research ethics approval | #24 | Plans for seeking research ethics committee / institutional review board (REC / IRB) approval | 12 |
| Protocol amendments | #25 | Plans for communicating important protocol modifications (eg, changes to eligibility criteria, outcomes, analyses) to relevant parties (eg, investigators, REC / IRBs, trial participants, trial registries, journals, regulators) | 12 |
| Consent or assent | #26a | Who will obtain informed consent or assent from potential trial participants or authorised surrogates, and how (see Item 32) | 5 |
| Consent or assent: ancillary studies | #26b | Additional consent provisions for collection and use of participant data and biological specimens in ancillary studies, if applicable | 5-6 |
| Confidentiality | #27 | How personal information about potential and enrolled participants will be collected, shared, and maintained in order to protect confidentiality before, during, and after the trial | 11-12 |
| Declaration of interests | #28 | Financial and other competing interests for principal investigators for the overall trial and each study site | 16 |

For peer review only

| | | | |
|---|---|---|---|
| Data access | #29 | Statement of who will have access to the final trial dataset, and disclosure of contractual agreements that limit such access for investigators | 11 |
| Ancillary and post trial care | #30 | Provisions, if any, for ancillary and post-trial care, and for compensation to those who suffer harm from trial participation | n/a |
| Dissemination policy: trial results | #31a | Plans for investigators and sponsor to communicate trial results to participants, healthcare professionals, the public, and other relevant groups (eg, via publication, reporting in results databases, or other data sharing arrangements), including any publication restrictions | 12 |
| Dissemination policy: authorship | #31b | Authorship eligibility guidelines and any intended use of professional writers | 12 |
| Dissemination policy: reproducible research | #31c | Plans, if any, for granting public access to the full protocol, participant-level dataset, and statistical code | 11 |
| **Appendices** | | | |
| Informed consent materials | #32 | Model consent form and other related documentation given to participants and authorised surrogates | Appendices 4 and 5 |
| Biological specimens | #33 | Plans for collection, laboratory evaluation, and storage of biological specimens for genetic or molecular analysis in the current trial and for future use in ancillary studies, if applicable | 8-9 |

None The SPIRIT Explanation and Elaboration paper is distributed under the terms of the Creative Commons Attribution License CC-BY-NC. This checklist can be completed online using https://www.goodreports.org/, a tool made by the EQUATOR Network in collaboration with Penelope.ai

For peer review only

# Supplementary Appendix 3: The Tessa Jowell BRAIN MATRIX Platform study World Health Organization Trial Registration Data Set

| **Data category** | **Information** |
|---|---|
| Primary registry and trial identifying number | ClinicalTrials.gov<br>NCT04274283 |
| Date of registration in primary registry | 18-Feb-2020 |
| Secondary identifying numbers | ISRCTN 14218060 |
| Source(s) of monetary or material support | The Brain Tumour Charity<br>Genomics England<br>INNOVATE UK |
| Primary sponsor | University of Birmingham |
| Secondary sponsor(s) | n/a |
| Contact for public queries | Dr Joshua Savage: brainmatrix@trials.bham.ac.uk |
| Contact for scientific queries | Prof. Colin Watts: c.watts.2@bham.ac.uk |
| Public title | The Tessa Jowell BRAIN MATRIX Platform Study |
| Scientific title | A British Feasibility Study of Molecular Stratification and Targeted Therapy to Optimize the Clinical Management of Patients With Glioma by Enhancing Clinical Outcomes, Reducing Avoidable Toxicity, Improving |



For peer review only - http://bmjopen.bmj.com/site/about/guidelines.xhtml

| Data category | Information |
|---|---|
| | Management of Post-operative Residual & Recurrent Disease and Improving Survivorship |
| Countries of recruitment | UK |
| Health condition(s) or problem(s) studied | Newly diagnosed suspected, or progressive with known, WHO Grade 2-4 glioma |
| Intervention(s) | None |
| Key inclusion and exclusion criteria: | Ages eligible for study: ≥16 years<br>Sexes eligible for study: both<br>Accepts healthy volunteers: no |
| | Inclusion criteria: Newly diagnosed suspected, or progressive with known, WHO Grade 2-4 glioma |
| | Exclusion criteria: Primary spinal cord tumours, active treatment of other malignancy |
| Study type | Observational |
| | Primary purpose: Feasibility of establishing an integrated histological-molecular diagnosis of glioblastoma |
| Date of first enrolment | 14-Dec-2020 |
| Target sample size | 1000 |
| Recruitment status | Open |
| Primary outcome(s) | • Time (from biopsy) to integrated histological–molecular diagnosis (TTMD) using whole genome sequencing and epigenomic classification |



For peer review only - http://bmjopen.bmj.com/site/about/guidelines.xhtml

| Data category | Information |
|---|---|
| Key secondary outcome(s) | • Time to completion of each node of tissue and imaging pathway. <br> • Tumour and biological sample(s) quality control (QC) status. <br> • Imaging QC status. <br> • Inter-rater agreement of RANO assessments. |

For peer review only



For peer review only - http://bmjopen.bmj.com/site/about/guidelines.xhtml

*To be printed on hospital headed paper*

# TESSA JOWELL BRAIN MATRIX

**A BRitish feasibility study of molecular stratification and targeted therapy to optimize the clinical mAnagement of patIeNts with glioMA by enhancing clinical ouTcomes, Reducing avoIdable toXicity, improving management of post-operative residual & recurrent disease and improving survivorship**

## Platform Study

# Informed Consent Form

| | | | |
|---|---|---|---|
| **Site:** | ____________________ | **Patient's Study Number:** | ☐☐☐☐ |
| **Principal Investigator:** | ____________________ | | |

**Sponsor's Protocol Number: RG_18-258**

**Please initial each box**

1. I confirm that I have read and understand the Patient Information Sheet (version ................... dated.........................) for the above study. I have had the opportunity to consider the information, ask questions and have had these answered satisfactorily. ☐

2. I understand that my participation is voluntary and that I am free to withdraw at any time without giving any reason, without my medical care or legal rights being affected. ☐



For peer review only - http://bmjopen.bmj.com/site/about/guidelines.xhtml

**3.** I give my permission for my initials, date of birth, hospital number, NHS/CHI number, Genomics England Referral ID number, histopathology numbers, and radiology numbers to be given to the BRAIN MATRIX Study Office when I am registered to the study as well as a copy of this consent form. I understand that copies of the consent form may be forwarded to other healthcare professionals to prove that I am taking part in the study. I also give permission for my information to be shared with other hospitals involved in my care and with the National Cancer Registration Service.

**4.** I understand that relevant sections of my medical notes and data collected during the study may be looked at by individuals from the BRAIN MATRIX Study Office, regulatory authorities, Sponsors and/or NHS bodies, where it is relevant to my taking part in this research. I understand that this information will be held in a confidential manner. I give permission for these individuals to have access to my records.

**5.** I agree to the collection of blood samples, or any other biofluid samples, from me, and consent for these samples to be analysed (including genetic analysis) as part of research associated with this study, to be used in biobanking and for future ethically approved studies.

**6.** I agree to the collection of tissue from any of my previous biopsies and future tumour surgeries (including "surgical access tissue") and consent for these samples to be analysed (including genetic analysis) as part of research associated with this study, to be used in biobanking and for future ethically approved studies.

**7.** I understand that identifiable data from the study may be shared with other institutions involved in BRAIN MATRIX (University of Edinburgh, University of Oxford, Genomics England, BRIAN databank run by The Brain Tumour Charity).

**8.** Anonymised linked data may also be provided to other 3rd parties (e.g. other academic institutions, pharmaceutical companies and QMENTA) for research and safety monitoring purposes and may be placed in recognised medical research data repositories, in accordance with best research practice.

**9.** I agree to my GP being informed of my participation in this study and to my GP being sent a copy of this Patient Information Sheet.

**10.** I agree to the collection of different aspects of my health data from the NHS and other Department of Health organisations, in addition to my medical records. The collection and analysis of my health data for research will continue across my entire lifetime and beyond.

**11.** I agree to take part in the above study.



For peer review only - http://bmjopen.bmj.com/site/about/guidelines.xhtml

**The following are <u>optional</u> and will not affect entry into the study, please initial in the relevant box:**

| | No | Yes |
|---|---|---|
| If in the event that an abnormality that might affect other family members is uncovered during genetic testing for the study, I would like my doctor to be informed and to be referred to a genetic counsellor if appropriate. | | |
| I would like to receive a copy of the lay summary results at the end of the study. | | |
| I agree that my doctor, in consultation with the BRAIN MATRIX Study Office, together with Genomics England and other NHS staff, can contact me if the data or samples reveal any clinical trials or other research that I might benefit from. If something is relevant to me, there is a process by which this will be shared with my NHS clinical team. | | |

| | | |
|---|---|---|
| **Name of patient** | **Date** | **Signature** |
| **Name of person taking consent**<br>You must have signed the<br>Site Signature & Delegation Log | **Date** | **Signature** |

For peer review only


For peer review only - http://bmjopen.bmj.com/site/about/guidelines.xhtml

*To be printed on local hospital headed paper*

# TESSA JOWELL BRAIN MATRIX

**A BRitish feasibility study of molecular stratification and targeted therapy to optimize the clinical mAnagement of patIeNts with glioMA by enhancing clinical ouTcomes, Reducing avoIdable toXicity, improving management of post-operative residual & recurrent disease and improving survivorship**

## Platform Study

The **Tessa Jowell BRAIN MATRIX** is a programme of work, the principal purpose of which is to improve the knowledge of, and treatment for, glioma, a type of brain tumour.

Brain tumours arise due to changes in the DNA and other molecules in cells of the brain. Different types of gliomas can have different changes and these can be used to determine a precise 'molecular diagnosis'. The ultimate goal for the Tessa Jowell BRAIN MATRIX is to learn how to use these molecular changes to more precisely determine what exact type of tumour patients have, and to identify, decide and test whether specific 'targeted' treatments could improve the survival and/or quality of life of patients with brain tumours.

The Tessa Jowell BRAIN MATRIX Platform Study forms the backbone of this programme. You have been given this information because you either have an existing brain tumour, or have recently been diagnosed with a brain tumour, likely to be a glioma.

In the platform study we aim to develop the infrastructure to provide rapid and accurate molecular diagnosis and the infrastructure to deliver clinical trials of new therapies in the future.

**The diagram below shows a summary of the Tessa Jowell BRAIN MATRIX Programme:**

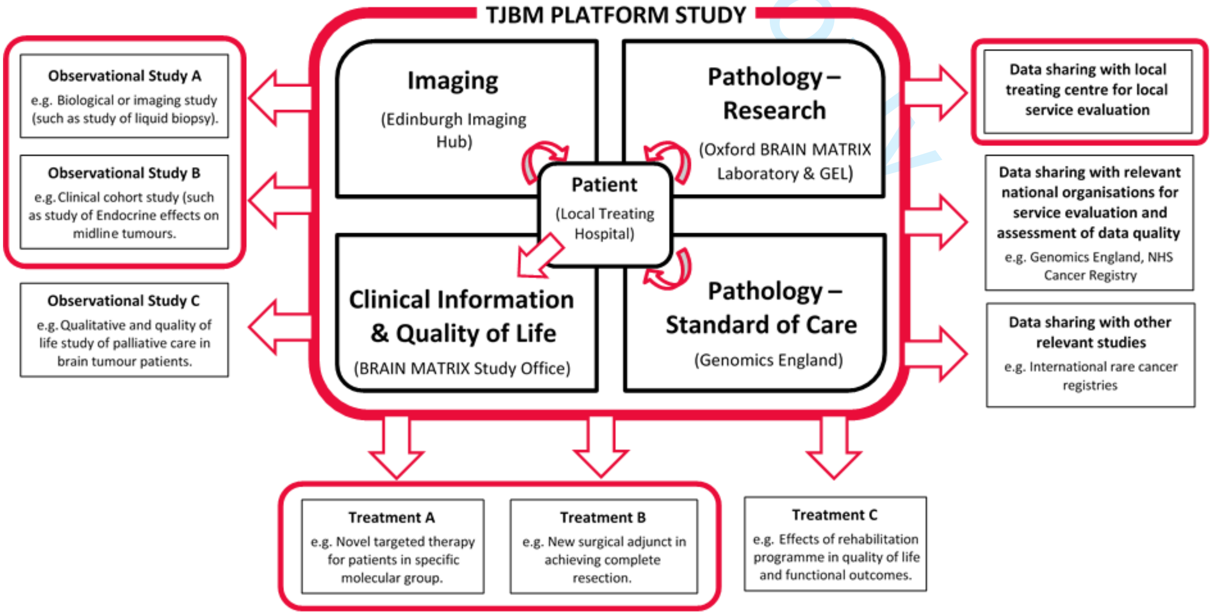


For further information about the Tessa Jowell BRAIN MATRIX Platform Study, please ask your doctor.


For peer review only - http://bmjopen.bmj.com/site/about/guidelines.xhtml

*To be printed on local hospital headed paper*

TESSA JOWELL BRAIN MATRIX

**A BRitish feasibility study of molecular stratification and targeted therapy to optimize the clinical mAnagement of patIeNts with glioMA by enhancing clinical ouTcomes, Reducing avoIdable toXicity, improving management of post-operative residual & recurrent disease and improving survivorship**

## Platform Study

# Patient Information Sheet

*To be used for patients over 16 years of age considering entering the Platform Study*

## We invite you to take part in a clinical study

We would like to invite you to take part in a non-commercial clinical study run by the University of Birmingham, supported by The Brain Tumour Charity.

Before you decide whether you would like to participate in this study, we would like you to understand:

- Why the research is being done.
- What it would involve for you.

A member of the team at your local hospital involved in this study will go through this information sheet with you and give you a copy to take away with you to discuss with friends and/or relatives, if you wish. They will answer any questions that you may have. If there is anything that is not clear within the information sheet, please ask your medical team.

**Take your time to decide whether or not you wish to take part. If you decide not to take part your doctors will continue to treat you in line with standard treatment and it will not affect the quality of your care.**

## Contents

**Part 1: What is this clinical study about?**

1. What is the purpose of this study?
2. What will happen to me if I take part?
3. What are the possible benefits and risks of taking part?
4. What happens at the end of the study?
5. Will anybody get paid if I take part?
6. What if there is a problem?
7. Will my taking part in this study be kept confidential?

**Part 2: What else do I need to know about taking part in this clinical study?**

1. More information about taking part
2. Who should I contact for further information



For peer review only - http://bmjopen.bmj.com/site/about/guidelines.xhtml

## Important things that you need to know

We want to improve the outcome for people like yourself who are living with a brain tumour. Specifically, in this study we are establishing a platform to deliver:

- Rapid and accurate detailed laboratory analysis of the molecular changes in brain tumours. This will enable accurate diagnosis in a timeframe useful to clinicians.
- To develop robust ways of collecting, processing and storing brain tumour tissue, radiology images and clinical data.
- An enhanced network of UK brain tumour clinical centres.
- The introduction of clinical trials of therapies tailored/targeted to the precise molecular and clinical features of individual patients.

## Who is Tessa Jowell?

This study is named after Baroness Tessa Jowell, the ex-politician and minister, who died from a glioma in summer 2018. As a result of her efforts to raise awareness of brain tumours, research funding is now being increased.

## Abbreviation and definitions

- Biofluids — Bodily samples that are liquid (e.g. blood, urine, saliva, tears, or cerebrospinal fluid
- DNA — Deoxyribonucleic acid
- GDPR — General Data Protection Regulation
- GEL — Genomics England
- Glioma — A type of brain tumour
- NHS — National Health Service
- REC — Research Ethics Committee
- TJBM — Tessa Jowell BRAIN MATRIX

We have sought to make this document readable and understandable by patients by involving patient representatives in writing this document. However, due to the nature of the study, and the legal and regulatory requirements to include certain information, we are aware that some of the wording can seem complex. This makes it especially important that you ask your research team about anything that you do not fully understand.

If you have any further questions, you are very welcome to contact the research team.

For peer review only



For peer review only - http://bmjopen.bmj.com/site/about/guidelines.xhtml

# Part 1:
# What is this clinical study about?

## 1. What is the purpose of this study?

### What is the purpose of this study?

The **Tessa Jowell BRAIN MATRIX** is a programme of work, the principal purpose of which is to improve the knowledge of, and treatment for, glioma, a type of brain tumour. The platform study forms the backbone for this.

Brain tumours are a challenging disease to treat. This is due to:

- The tumour's location within the brain, and its tendency to grow into nearby brain tissue, often make it very difficult or not possible to remove the tumour completely with surgery.
- The biology of brain tumours, including:
  - Their lack of response to traditional radiotherapy/chemotherapies.
  - Their ability to spread within the brain.
  - Their ability to change/adapt during/in response to treatments.
- Difficulties in delivering drugs in adequate amounts to the tumour, due to natural defences of the brain (the 'blood-brain barrier').
- A relative lack in research infrastructure for brain tumour patients within the NHS and academia in the UK.

Together this means that progress in treating brain tumours has not matched that of other cancers. In this study, we want to create an underlying treatment and analysis 'platform' to address these challenges of treating gliomas. This platform will bring together many different people including patients like you, brain surgeons, cancer doctors, technicians and scientists.

The standard treatment for most brain tumours is surgery. This is to remove the main bulk of the tumour and is followed by radiotherapy and/or chemotherapy. In the majority of cases, however, it is difficult for all of the tumour cells to be removed by the surgeon because of the way it invades, and the potential damage that can occur to the brain by removing too much tissue. Therefore, some tumour cells can be left behind which can regrow. Whilst radiotherapy and/or chemotherapy can delay this, their effects are often only temporary. There is, therefore, a need to develop and test new treatments.

Brain tumours arise due to changes in the DNA and other molecules in cells of the brain. Different types of gliomas can have different changes and these can be used to determine a precise 'molecular diagnosis'. The ultimate goal for the **Tessa Jowell BRAIN MATRIX** is to learn how to use these molecular changes to more precisely determine what exact type of tumour you have and to identify, decide and test whether specific 'targeted' treatments could improve the survival and/or quality of life of patients with brain tumours.

To achieve this, we need to develop the infrastructure to provide a rapid and accurate molecular diagnosis. A large network of clinical hubs across the UK, with expertise in managing patients with brain tumours, will be developed. This will enable:

- Collection of high-quality clinical data, including information about your quality of life, in a secure manner, maintaining patient confidentiality.
- Robust processes and safeguards for the collection, processing, analysis and storage of tumour tissue, including feedback to your treating doctors of a precise molecular diagnosis.
- Collection and expert review of images to assess responses of tumours to treatment in a standardised manner and in a time frame relevant to a patient's treatment.


For peer review only - http://bmjopen.bmj.com/site/about/guidelines.xhtml

Once established this infrastructure will facilitate the rapid introduction of clinical trials testing targeted therapies tailored to the genetic changes of an individual's tumour. In addition, collection of this data, images and samples, will provide an extremely valuable resource to help us to understand better the clinical behaviour, impact on patients, molecular pathology and imaging characteristics of brain tumours. This will enable clinicians and scientists to improve the quality of treatments available and the quality of lives of patients with brain tumours. This study will actively engage with partners within the NHS, universities, pharmaceutical industry and charitable sectors to translate the advances into benefits for patients.

### Why have I been invited to participate?

In most cases, you have been invited to take part in this study because your recent scan shows that there is a growth in your brain. It is likely that this is a glioma, a type of brain tumour, and that you will be undergoing surgery (or biopsy) to remove at least some of this tumour.

In some cases, you have been asked because you are known to have such a tumour, and are undergoing further surgery, or samples are available that can be used from your previous operations.

### Do I have to take part?

No, you do not have to take part. It is up to you to decide whether to join the study. A member of the local research team will describe the study and go through this information sheet with you. If you decide to take part, you will be asked to sign a consent form to show you have agreed to participate. You are free to withdraw at any time, without giving a reason. A decision not to take part or to withdraw later will not affect the standard of care you receive thereafter.

## 2. What will happen to me if I take part?

### What will happen if I decide to take part?

If you decide to take part in this study, you will be given this information sheet to keep, and you (and your study doctor) will be asked to sign a consent form to show that you have agreed to take part in this study. You will be given a copy of the signed consent form to take home with you. We will then need to confirm that you meet all the requirements and that you are fit enough to take part in the study. This is called screening. If you do not meet the inclusion criteria for this study you will not be able to participate. Decisions about your treatment are independent to your participation in the BRAIN MATRIX Platform Study and will continue to be made in discussion with you and your doctor.

For peer review only



For peer review only - http://bmjopen.bmj.com/site/about/guidelines.xhtml

**The diagram below shows a summary of the Tessa Jowell BRAIN MATRIX Programme:**

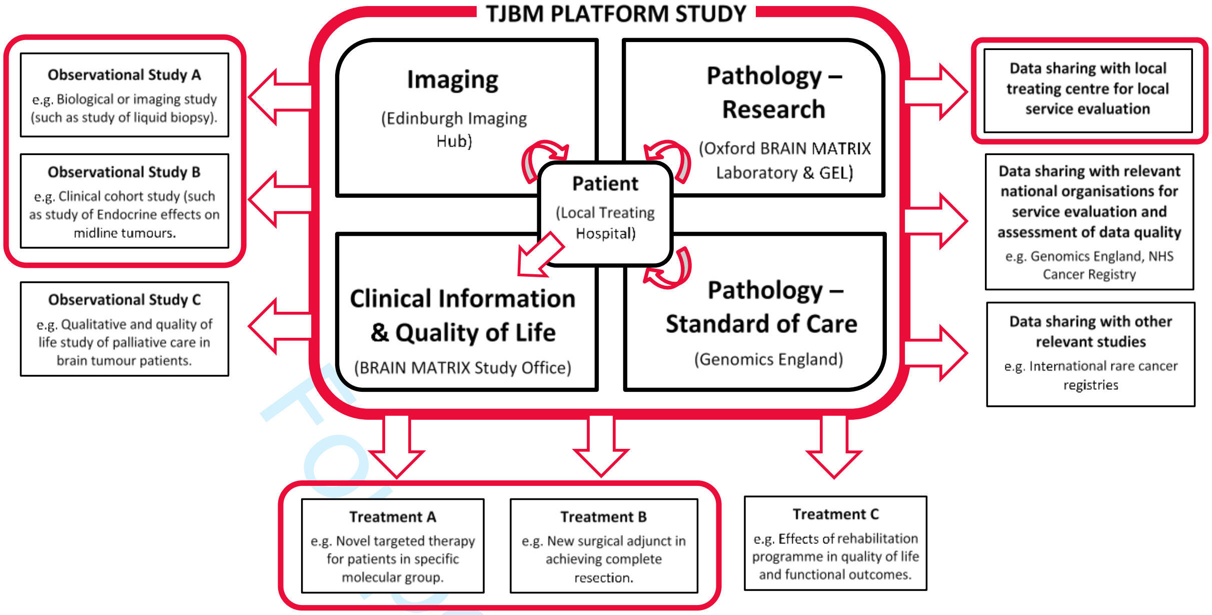


## Study Participation

If you are eligible for this study you will either have had, or be about to have, surgery for your tumour. As part of this study, tumour removed during your operation will be analysed to look for specific molecular changes. No additional surgery or extra tumour will be taken as a result of participation in this study. As with normal standard care, your tumour will be analysed by your local pathologist. A small part will be sent for review by experts and advanced molecular analysis will be undertaken (in most cases analyses called whole genome sequencing and epigenetic classification) to get a detailed understanding of the DNA/molecular changes within your tumour. These results will be fed back to the doctor treating you. It is intended that this will occur within 28 days; however, it may be longer while the study becomes fully operational. If samples are available from previous surgery to your tumours, we may also analyse these. Similarly, if available, other relevant samples, such as cerebrospinal fluid, collected as part of your care, may also be analysed. In addition, as technologies and analyses improve our understanding of brain tumours, we may find important results at a later date. These will be fed back to your doctor.

To perform these analyses you will be asked to give a blood sample, which will also be analysed to look at the molecular features, including of your DNA. This is required to identify what ‘new’ changes have occurred in your tumour.

Following your surgery you will continue with other treatment(s) as directed by your doctor. Treatment generally involves radiotherapy and chemotherapy and your doctor will discuss this with you. As is standard practice, you will be closely monitored for signs of disease progression and the effects of the treatment given.

As part of this study, information on your treatments and disease will be collected. This will include details about, your current health (e.g. as assessed by your doctor's assessment and examination, some blood test results), treatments you are taking/receiving (e.g. names of drugs, doses). Images from brain scans you undergo, along with relevant clinical information, will also be sent to and stored by the University of Edinburgh, and where appropriate, undergo expert review by a panel of



For peer review only - http://bmjopen.bmj.com/site/about/guidelines.xhtml

radiologists with expertise in brain tumours. You will not have any additional scans as part of this study. If you have further surgery, some of the tissue removed may also be analysed. As above, no additional surgery will be performed or extra-tumour will be removed as a result of participation in this study. You may also be asked to provide additional optional biofluid samples at the start or end of any specific treatments (e.g. at the start and end of radiotherapy).

We know that brain tumours can impact many areas of your life and we want to understand this better. To do this you will be asked to complete a 'Quality of Life' booklet that consists of four short questionnaires, initially when you join the study, then at key points in your treatment and when you are being monitored after treatment. These will generally be when you are attending the hospital to have your brain scans. A carer may assist you with completing the questionnaire, but the answers should be your own.

For peer review only

Patients joining BRAIN MATRIX will also be able to support a study, called BRIAN (the Brain tumouR Information and Analysis Network), run by the The Brain Tumour Charity.

BRIAN allows people to record their experiences of having a brain tumour using a mobile device or web app. This is to help doctors and researchers get a better understanding of how having a brain tumour affects the quality of life of patients who are living with a brain tumour or caring for someone who has a brain tumour. Your doctor will talk to you about participating in this additional study. In the future it is hoped that by analysing information collected by BRIAN together with all the other data collected from patients participating in BRAIN MATRIX, it will be possible to provide better overall care and support to patients and their families.

You can find out more about BRIAN and how to take part at the Brain Tumour Charity's website: https://www.thebraintumourcharity.org/living-with-a-brain-tumour/brian/.

You are welcome to take part in BRIAN even if you do not wish to take part in this study. Similarly, you can still participate in BRAIN MATRIX if you do not wish to join BRIAN.

## How long will I be in the study?

We would like to continue to collect data on you for up to five years where possible.

## What will I have to do?

If you decide to take part in this study, you will be required to:

- ✓ Sign a consent form to enter the study.
- ✓ Keep all scheduled medical and imaging appointments. (There are no additional appointments or scans as part of this study).
- ✓ At routine clinic visits your weight, neurological examination and performance status (measure of your general health and how your disease affects your daily routine) will be taken.
- ✓ Tell the study doctor about any medication that you are taking, even if it is medicine you buy without a prescription or is a natural or herbal remedy.
- ✓ Tell the doctor about any side effect, injury, symptom or complaint you experience, including any unplanned hospital admissions.
- ✓ Provide blood samples, and further optional biofluid samples, at the start and end of a treatment, and/or at tumour progression.
- ✓ Complete Quality of Life questionnaires when you join the study and when requested (usually at the start of a treatment, and/or at tumour progression, and otherwise every 3-6 months).


For peer review only - http://bmjopen.bmj.com/site/about/guidelines.xhtml

### What are the alternatives for treatment?

If you choose not to take part in this study you will receive the same treatments as if you were on the study.

## 3. What are the possible benefits and risks of taking part?

### What are the possible benefits of taking part?

We cannot promise that you will benefit directly from participating in this study. All the information that we get from this study will help improve the treatment of patients with glioma in the future.

By participating in this study, it will allow us to identify certain molecular characteristics of your tumour. These analyses can help refine and, in some cases, change the diagnosis. Your doctor will then be able to adapt and discuss the best treatment options for you.

This study may identify molecular changes in your tumour that suggest you could benefit from new-targeted treatments, which could be made available to you in the future or make you eligible for another clinical study/trial. Your doctor will discuss your options with you.

**Please note that identification of a target does not guarantee benefit from, or access to, a targeted treatment.**

### What are the possible disadvantages and risks of taking part?

We want to try and improve the outcome for patients with glioma and believe that providing this standardised platform may improve outcomes in, and options for, patients. However, it is possible that this may not show any benefit over the current UK standard practice.

### What about genetic results?

In addition to identifying changes in your tumour, it is possible that the genetic analysis might discover an unexpected genetic change in your normal cells. Genetic changes in normal cells may tell your doctors that you could be at risk of another disease. This might be a cancer or another disease that has nothing to do with your cancer. Changes like this could be present in other members of your family or passed on to your children. It is important to emphasise that the aim of this study is not to identify genes that may be inherited and cause cancer in families.

In the unlikely event that studies on your blood or tumour reveal genetic information that may affect you or other family members, we need to know whether you would wish to be informed. If you wish to be informed about these genetic changes, please initial the corresponding box on the informed consent form. We will then tell the doctor treating you at your local centre so that they can discuss the finding with you and arrange for any genetic counselling you may need. Please note that these unexpected genetic findings will not affect how your brain tumour is treated and will not prevent you from participating in this study.

You may choose to opt in or opt out of receiving information about genetic changes in normal cells on the informed consent form.

### What are the side effects of the treatment?

There are no specific treatments within this study. Your doctor will discuss the side effects of your recommended treatment.


For peer review only - http://bmjopen.bmj.com/site/about/guidelines.xhtml

## 4. What happens at the end of the study?

The study team will continue to collect data about your health and further treatment for up to 5 years where possible. This data will be collected at routine clinic appointments.

## 5. Will anybody get paid if I take part?

You will not receive any money or study expense reimbursement for taking part in this clinical study.

## 6. What if there is a problem?

If you have any concerns about your care during this study or any possible harm you may suffer, you should inform your study doctor immediately. More detailed information is given in Part 2.

## 7. Will my taking part in this study be kept confidential?

Yes. We will follow ethical and legal practice and all information about your participation in this study will be kept confidential. The details of this are in Part 2 of this information sheet.

For peer review only

**This completes Part 1 of the Information Sheet**

If the information in Part 1 has interested you and you are considering participation, please read the additional information in Part 2 before making any decision.


For peer review only - http://bmjopen.bmj.com/site/about/guidelines.xhtml

# Part 2:
# What else do I need to know about taking part in this clinical study?

## 8. More information about taking part

### What if relevant new information becomes available?

Sometimes new information about the conditions being studied becomes available during the course of a research study. If this happens, your study doctor will tell you about it and discuss this with you. Your treatment is not affected by inclusion in this study, and therefore it is unlikely to influence whether you should continue in the study. If you decide not to carry on, your study doctor will make arrangements for your care to continue as normal. If you decide to continue with the study then you may be asked to sign an updated consent form. If the study is stopped for any other reason, your doctor will tell you and arrange your continuing care.

### What if I decide that I don't want to carry on with the study?

You are free to withdraw from this study at any time. You do not have to give a reason for your decision and your future treatment will not be affected. Any data already collected prior to withdrawal may still be used for study purposes.

### What if there is a problem?

In the event that something does go wrong and you are harmed because of taking part in the trial, and this is due to someone's negilence, then you may have grounds for claiming compensation from the Sponsor of the trial (the University of Birmingham) or the NHS Trust who treated you but you may have to pay your legal costs.

NHS Trust and non-Trust Hospitals have a duty of care to patients treated, whether or not the patient is taking part in a clinical trial, and the normal NHS complaints mechanism will still be available to you (if appropriate).

### What will happen to any samples I give?

Collection of diagnostic biopsy samples, taken as part of your standard care, and blood samples at study entry are mandatory and are required for the genomic analysis to be performed. In addition, we will ask that pathology reports on your tissue samples are sent to the BRAIN MATRIX Study Office and to University of Oxford. Any samples remaining at the end of this analysis will be stored in a biobank and may be used in future ethically approved research, which may involve genetic analysis, animal or *in vitro* models, commercial or private institutions, and which may take place in the UK or overseas. If you change your mind about the use of your samples in the study, we will not collect any more blood samples and, if you request it, we will destroy all that remains of samples already taken.

### Will my taking part in this study be kept confidential?

Yes. All information collected about you for this study will be subject to the General Data Protection Regulation (GDPR) and the Data Protection Act 2018 and will be kept strictly confidential. University of Birmingham is the Sponsor for this study based in the UK. We will be using information from your medical records in order to undertake this study and will act as the data controller for this study. This means that we are responsible for looking after your information and using it properly. University of Birmingham and the NHS will keep identifiable information about you for at least 25 years after the study has finished, allowing the results of the study to be verified if needed.



For peer review only - http://bmjopen.bmj.com/site/about/guidelines.xhtml

All information collected by the Sponsor will be securely stored at CRCTU at the University of Birmingham (BRAIN MATRIX Study Office) on paper and electronically and will only be accessible by authorised personnel. The only people at the University of Birmingham who will have access to information that identifies you will be people who manage the study or audit the data collection process. With your permission, your study doctor will provide your initials, date of birth, NHS/CHI number and hospital number when they enter you into the study and they will notify your GP that you intend to participate. They will also send a copy of your signed Informed Consent Form in the post to the BRAIN MATRIX Study Office.

The NHS will use your name and contact details to contact you about the research study, and make sure that relevant information about the study is recorded for your care, and to oversee the quality of the study. Your study doctor or research nurse may also need to send a copy of your Informed Consent Form to other healthcare professionals (e.g. your GP or NHS pathologist) to prove that you have given consent to take part in the study before they will provide information or tumour samples.

In the BRAIN MATRIX Study Office you will be identified by a unique study number. In routine communication between your hospital and the BRAIN MATRIX Study Office you will only be identified by your study number, initials and date of birth. We may need to record, and occasionally refer to you using your hospital, Genomics England (GEL) Referral ID number and histopathology numbers during analysis of your tumour samples and radiology numbers during analysis of your MRI scans. In addition, your study number, hospital, NHS, histopathology and radiology numbers may be included on samples sent to University of Oxford, University of Edinburgh and Genomics England for central review to help specialist pathologists and radiologists identify the tumour samples and MRI data.

All information will be treated as strictly confidential and nothing that might identify you will be revealed to any third party other than those involved in the treatment or organisation of tumour sample and imaging collection and transfer (e.g. staff at University of Birmingham, University of Oxford, University of Edinburgh and Genomics England). It may be necessary to send information about you such as study number and date of birth to the BRIAN databank (IRAS ID: 237931) run by The Brain Tumour Charity. This is for your, and others', protection to track the safety of the treatments used. We will also provide anonymised linked data to QMENTA, a neuroimaging storage platform, for research purposes. All third parties have the same duty of confidentiality to you as all other research study personnel.

By taking part in the study you will be agreeing to allow research staff at your hospital, and from the BRAIN MATRIX Study Office, to look at the study records, and this includes your medical records. It may be necessary to allow authorised personnel from the University of Birmingham and/or NHS bodies to have access to your medical and research records. This is to ensure that the study is being conducted to the highest possible standards. We may also collect different aspects of your health data from the NHS and other Department of Health organisations, in addition to your medical records, for the purposes of long term followup.

From time to time we may be asked to share the study information (data) we have collected with researchers running other studies in this organisation and in other organisations, so that they can perform analysis on the data to answer other important questions about brain tumours. This may also reveal clinical trials or other research that you might benefit from, which your doctor or another member of your clinical team may discuss with you if appropriate. These organisations may be universities, NHS organisations or companies involved in health research and may be in this country or


For peer review only - http://bmjopen.bmj.com/site/about/guidelines.xhtml

abroad. Any such request is carefully considered by the study researchers and will only be granted if the necessary procedures and approvals are in place. This information will not identify you and will not be combined with other information in a way that could identify you. The information will only be used for the purpose of health research, and cannot be used to contact you or to affect your care. It will not be used to make decisions about future services available to you, such as insurance. Under no circumstances will you be identified in any way in any report, presentation or publication arising from this or any other study.

All individuals who have access to your information have a duty of confidentiality to you.

If you choose to withdraw from the study treatment, we would still like to collect relevant information about your health, as this will be invaluable to our research. If you have any objection to this, please let your study doctor know.

You can withdraw your consent to our processing of your data at any time. Your rights to access, change, or move your information are limited, as we need to manage your information in specific ways in order for the research to be reliable and accurate. If you withdraw from the study, we will keep the information about you that we have already obtained. To safeguard your rights, we will use the minimum personally-identifiable information possible. Under the provisions of the General Data Protection Regulation you have the right to know what information the BRAIN MATRIX Study Office have recorded about you. If you wish to view this information or find more about how we use this information, please contact Legal Services at the address below.

Legal Services
University of Birmingham
Edgbaston
BIRMINGHAM
B15 2TT

More information about how your data will be handled can be found in the CRCTU Privacy Policy available on our website: www.birmingham.ac.uk/crctu.

## Where can you find out more about how your information is used?

You can find out more about how we use your information in the GDPR Guidance document provided to you with this information sheet.

## What will happen to the results of the study?

Results from the study will be published in medical journals but no individual patients will be identified. You will be able to get a copy of the published results by asking your doctor at the end of the study.

## Who is organising and funding the research?

This research study is being carried out by a network of doctors across the UK. The study is sponsored by the University of Birmingham and co-ordinated by the Cancer Research UK Clinical Trials Unit at the University of Birmingham. The study is being funded by The Brain Tumour Charity, and some aspects of the genomic testing will be supported by Genomics England, part of Department of Health & Social Care. Your study doctor will not be paid extra for including you in this study.


For peer review only - http://bmjopen.bmj.com/site/about/guidelines.xhtml

## Who has reviewed the study?

All research in the NHS is looked at by an independent group of people, called a Research Ethics Committee (REC) to protect your safety, rights, well-being and dignity. This study has been reviewed and given favourable ethical opinion by the West Midlands – Edgbaston Research Ethics Committee. It has also been approved by the local Trust Research and Development department at your hospital.

## Involvement of the General Practitioner/Family Doctor (GP)

It is important that your GP is informed that you are taking part in this study. In addition, we may ask him/her to provide information on your progress. If we need to contact your GP for any follow-up information, we will need to use your full name in our correspondence.

# 9. Who should I contact for further information?

## Further information and contact details

If you have any questions or concerns about your disease or this clinical study, please discuss them with your doctor.

For more generic information about treating cancer, you may also find it helpful to contact '**About Cancer**', an information service about cancer and cancer research studies by Cancer Research UK:

**Freephone:** 0808 800 40 40

**Website:** www.cancerresearchuk.org/about-cancer/brain-tumours

For more information and support about brain tumours, you may find it helpful to contact **The Brain Tumour Charity**:

**Freephone:** 0808 800 0004

**Email:** support@thebraintumourcharity.org

**Website:** www.thebraintumourcharity.org/

## Your telephone contact numbers:

**Local Investigator:** ............................................ **Contact Number:** ............................................

**Research Nurse:** ............................................ **Contact Number:** ............................................

**Emergency (24 hours) Contact Number:** ........................................

**Thank you for taking time to read this Patient Information Sheet and considering taking part in this study.**



For peer review only - http://bmjopen.bmj.com/site/about/guidelines.xhtml

# Summary of Assessments

| Assessment / Activity | Screening | Platform Entry | Day 1 -28 | Start of concomitant/ adjuvant treatment | Further Surgery *(if applicable)* | Follow-up – up to 5 years |
|---|---|---|---|---|---|---|
| Informed Consent | ✓ | | | | | |
| Confirm eligibility | ✓ | | | | | |
| Registration | | ✓ | | | | |
| Baseline Clinical Data Collection | | ✓ | | | | |
| Diagnostic Surgery | | ✓ | | | ✓ | |
| Collection of blood sample (mandatory) | | ✓ | | | | |
| Collection of blood samples (optional) | | ✓ | | ✓ | ✓ | ✓ |
| Neurological Examination | | ✓ | | ✓ | ✓ | ✓ |
| Performance Status | | ✓ | | ✓ | ✓ | ✓ |
| Weight | | ✓ | | ✓ | ✓ | ✓ |
| Quality of Life Questionnaires | | ✓ | | ✓ | ✓ | ✓ |
| Blood and biopsy samples sent to Oxford | | ✓ | | | ✓ | |
| Imaging data sent to Edinburgh | | ✓ | | ✓ | ✓ | ✓ |
| Local pathology report sent to BRAIN MATRIX Study Office and Oxford | | | ✓ | | ✓ | |
| Molecular testing and feedback | | | ✓ | | ✓ | |
| Reporting of relapse, further treatment | | | ✓ | ✓ | ✓ | ✓ |

For peer review only



For peer review only - http://bmjopen.bmj.com/site/about/guidelines.xhtml

## Supplementary Appendix 6: The Tessa Jowell BRAIN MATRIX Platform study schedule of events

| Activity* | Screening | Platform Entry | Day 1-28 | Initiation of concomitant therapy | Initiation of adjuvant therapy | Further surgery (if applicable) | Follow-up – up to 5 years# | Death |
|---|---|---|---|---|---|---|---|---|
| Informed consent[1] | X | | | | | | | |
| Confirm eligibility | X | | | | | | | |
| Registration | | X | | | | | | |
| Baseline Clinical Data Collection[2] | | X | | | | | | |
| Collection of blood for germline DNA | | X | | | | | | |
| Collection of Liquid biopsies – blood[3] | | X | | X | X | X | X | |
| Diagnostic surgery (biopsy or craniotomy) | | X | | | | X | | |
| Shipment of matched tumour and blood sample to Oxford BRAIN MATRIX Laboratory for molecular diagnosis[4] | | X | | | | X | | |
| Transfer of pseudo-anonymised imaging data to Edinburgh Imaging Hub[5] | | X | | X | X | X | X | |
| Clinician Global Impression of Change[6] | | | | X | X | X | X | |
| NIH Stroke Score[7] | | X | | X | X | X | X | |
| Weight[8] | | X | | X | X | X | X | |
| WHO Performance Status[9] | | X | | X | X | X | X | |
| Quality of Life questionnaires[10] | | X | | X | X | X | X | |
| Local pathology report sent to BRAIN MATRIX Study Office[11] | | | X | | | X | | |



For peer review only

For peer review only - http://bmjopen.bmj.com/site/about/guidelines.xhtml

| Activity* | Screening | Platform Entry | Day 1-28 | Initiation of concomitant therapy | Initiation of adjuvant therapy | Further surgery (if applicable) | Follow-up – up to 5 years# | Death |
|---|---|---|---|---|---|---|---|---|
| Reporting of relapse, further treatment and death | | | X | X | X | X | X | X |
| Post-mortem report and submit tissue specimens to CRUK PEACE study[12] | | | | | | | | X |

**Notes**

* Where applicable and acceptable in accordance to local practices, visits/assessments may be performed by telephone or video call.

\# Follow-up visits to occur every 6 months and at clinic visit that coincides with the time point.

1. Written informed consent must be obtained within 28 days of Platform Entry and before any study-specific screening procedures.
2. Baseline Clinical Data Collection to include height.
3. Further optional blood samples should be collected whenever possible for future analysis of cell-free circulating tumour DNA (cfT-DNA) at the following time points: At first post-operative MRI; Initiation of adjuvant treatment (if applicable); the end of adjuvant treatment (if applicable); the time of objectively measured progression; during the palliative phase if a post-mortem has been agreed. Cerebrospinal fluid, if available for the patient, should also be submitted.
4. Local sites will register their samples (blood and frozen tissue) after the patient has been registered to the study and send them to the Oxford BRAIN MATRIX Laboratory for biobanking, biofluid analysis and complementary omics. For those cases that are recruited through the NHS Genomic Medicine Service (GMS) WGS pathway for Whole Genome Sequencing (WGS) in England;, a (small) tumour sample and one EDTA blood sample will be sent directly to the respective Genomics Laboratory Hub (GLH), once standard of care paired tumour blood WGS analysis is established. Patients must be consented to both the NHS GMS WGS pathway and BRAIN MATRIX. A copy of the Sample Form must also be sent to the BRAIN MATRIX Study Office. Please refer to the BRAIN MATRIX Platform Laboratory Manual for further details. Clinical data to also be included on the Sample Form.
5. Refer to the Imaging Manual for further details. Clinical data to also be included on Imaging Form. Neurosurgical navigation and radiotherapy planning imaging to be submitted in addition to MRI.
6. **Error! Reference source not found.**To be collected at the start and end of concomitant therapy and start and end of adjuvant treatment. For Further Surgery time point, to be collected at the patient's post-operative review. The Clinician Global Impression of Change score should also be collected around the time of imaging and submitted with the imaging to facilitate RANO assessment. The Clinician Global Impression of Change can be completed by Investigators or Research Nurses.
7. **Error! Reference source not found.**To be collected at the start and end of concomitant therapy and start and end of adjuvant treatment. For Further Surgery time point, to be collected at the patient's post-operative review. The NIH Stroke Score can be completed by Investigators or suitably trained Research Nurses.



For peer review only - http://bmjopen.bmj.com/site/about/guidelines.xhtml

8. Weight to be collected at the start and end of concomitant therapy and start and end of adjuvant treatment. For Further Surgery time point, to be collected at the patient's post-operative review.
9. **Error! Reference source not found.**To be collected at the start and end of concomitant therapy and start and end of adjuvant treatment. For Further Surgery time point, to be collected at the patient's post-operative review.
10. QoL Questionnaires to be completed at follow-up visits that coincide with imaging appointments. **Error! Reference source not found.**
11. Pseudonymised copy of local pathology report for each sample must be sent to the BRAIN MATRIX Study Office as soon as it is available. Once received, it will be shared with the Oxford BRAIN MATRIX Laboratory for Genomic Tumour Advisory Board discussion. Submission of this report is the responsibility of the recruiting site.
12. Separate consent to the CRUK PEACE study (or equivalent) to be obtained.

For peer review only



For peer review only - http://bmjopen.bmj.com/site/about/guidelines.xhtml

# Supplementary Appendix 7: Definition of adverse events

## Adverse Event (AE)

Any untoward medical occurrence in a patient or clinical trial subject participating in the study which does not necessarily have a causal relationship with the treatment received.

Comment:

An AE can therefore be any unfavourable and unintended sign (including abnormal laboratory findings), symptom or disease temporally associated with the use of a medicinal product, whether or not related to the medicinal product.

## Related Event

An event which resulted from the administration of any of the research procedures.

## Serious Adverse Event (SAE)

An untoward occurrence that:

- Results in death
- Is life-threatening*
- Requires hospitalisation** or prolongation of existing hospitalisation
- Results in persistent or significant disability or incapacity
- Consists of a congenital anomaly/ birth defect
- Or is otherwise considered medically significant by the Investigator***

Comments:

The term severe is often used to describe the intensity (severity) of a specific event. This is not the same as serious, which is based on patients/event outcome or action criteria.

* Life threatening in the definition of an SAE refers to an event in which the patient was at risk of death at the time of the event; it does not refer to an event that hypothetically might have caused death if it were more severe.

** Hospitalisation is defined as an unplanned, formal inpatient admission, even if the hospitalisation is a precautionary measure for continued observation. Thus, hospitalisation for protocol treatment (e.g., line insertion), elective procedures (unless brought forward because of worsening symptoms) or for social reasons (e.g., respite care) are not regarded as an SAE.

For peer review only



For peer review only - http://bmjopen.bmj.com/site/about/guidelines.xhtml

*** Medical judgment should be exercised in deciding whether an AE is serious in other situations. Important AEs that are not immediately life threatening or do not result in death or hospitalisation but may jeopardise the subject or may require intervention to prevent one of the other outcomes listed in the definition above, should be considered serious.

## Unexpected and Related Event

An event which meets the definition of both an Unexpected Event and a Related Event.

## Unexpected Event

The type of event that is not listed in the protocol as an expected occurrence.

For peer review only


For peer review only - http://bmjopen.bmj.com/site/about/guidelines.xhtml
