# Supplementary material for: Protocol for the Tessa Jowell BRAIN MATRIX Platform Study
Source: BMJ Open. 2022 Sep 8;12(9):e067123. doi: 10.1136/bmjopen-2022-067123 (PMC9462095; doi:10.1136/bmjopen-2022-067123)
Supplement: Reviewer comments [file bmjopen-2022-067123.reviewer_comments.pdf]

PEER REVIEW HISTORY

BMJ Open publishes all reviews undertaken for accepted manuscripts. Reviewers are asked to complete a checklist review form (**http://bmjopen.bmj.com/site/about/resources/checklist.pdf**) and are provided with free text boxes to elaborate on their assessment. These free text comments are reproduced below.

ARTICLE DETAILS

**TITLE (PROVISIONAL)**	Protocol for the Tessa Jowell BRAIN MATRIX Platform Study
**AUTHORS**	Watts, Colin; Savage, Joshua; Patel, Amit; Mant, Rhys; Wykes, Victoria; Pohl, Ute; Bulbeck, Helen; Apps, John; Sharpe, Rowena; Thompson, Gerard; Waldman, Adam; Ansorge, Olaf; Billingham, Lucinda

This article was not externally reviewed at BMJ Open. Protocols that have been independently assessed prior to submission to BMJ Open are usually fast-tracked to publication on the grounds that further substantial changes will not be possible. This independent assessment will usually be external, independent review for both a substantial grant award from a non-commercial or government funder and ethics approval.
